# Decrypting the Nonadiabatic Photoinduced Electron Transfer Mechanism in Light-Sensing Cryptochrome

**DOI:** 10.1021/acscentsci.5c00376

**Published:** 2025-05-30

**Authors:** Gustavo J. Costa, Ruibin Liang

**Affiliations:** Department of Chemistry and Biochemistry, 6177 Texas Tech University, Lubbock, Texas 79409, United States

## Abstract

Cryptochromes are blue light photoreceptors in organisms from plants to animals that are essential for circadian rhythms, phototropism, and magnetoreception. In light-sensing cryptochromes, the photoexcitation of the flavin adenine dinucleotide (FAD) cofactor triggers a cascade of electron transfer (ET) events via a tryptophan chain, eventually generating a radical pair crucial for signaling. Despite extensive studies, the initial photoinduced ET from a neighboring tryptophan residue to FAD remains unclear due to the complexity of simulating all-atom dynamics in excited states, particularly regarding the roles of nonadiabatic pathways and protein environment on the reaction kinetics and quantum efficiency of the ET. To address this gap, we performed extensive nonadiabatic and adiabatic dynamics simulations with on-the-fly multireference *ab initio* electronic structure calculations of *Arabidopsis thaliana* cryptochrome 1 (*At*CRY1). Our results reveal a novel mechanism in which rapid nonradiative decay from higher-lying singlet states leads to charge separation, complementing the slower adiabatic ET on the S_1_ state hindered by a newly identified low-energy S_1_ local excitation minimum. Furthermore, the protein environment stabilizes tryptophan orientations, facilitating subsequent ET steps. These insights significantly enhance our understanding of photoinduced ET in cryptochromes and the structure–function relationships in photoreceptors.

## Graphical abstract

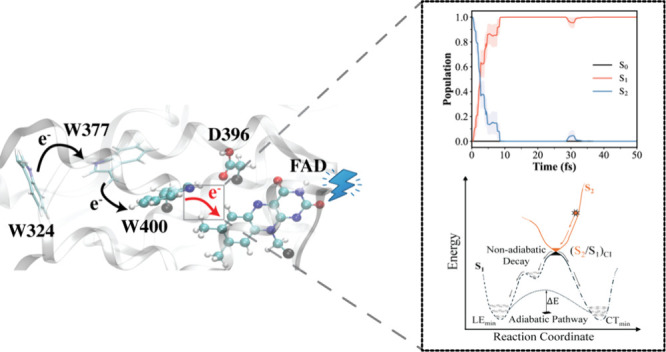

## Introduction

Cryptochromes are blue-light photoreceptors in plants and animals.^1–10^ They play essential roles in many biological processes, such as circadian rhythms,^2,5,11–13^ photomorphogenesis, and phototropism in plants,^1,3,14,15^ and the sensing of magnetic fields in migratory animals.^16–19^ Light-sensing cryptochromes bind the flavin adenine dinucleotide (FAD)^20^ as the cofactor, which absorbs blue light and induce long-range electron transfer (ET) across a chain of tryptophan residues. In its dark-adapted state, the isoalloxazine ring of FAD is fully oxidized.^21,22^ After the FAD is photoexcited, the nearest tryptophan residue donates an electron to the isoalloxazine ring of FAD. The rate of this first photoinduced ET step depends on the type of cryptochrome, ranging from ∼1 ps in animal cryptochromes such as CRY4 ^21,23,24^ and subpicosecond time scale in plant cryptochromes such as *At*CRY1.^19,25^ The subsequent ET steps through the chain of tryptophan residues eventually create a coupled radical pair separated by a long distance (>15 Å). This radical pair creates the signaling state of the cryptochrome.

A comprehensive understanding of the initial ET from the closest trptophan residue to the FAD is essential for elucidating how the radical pair is created and propagated to create the signaling state of cryptochromes. Despite numerous previous studies,^6,19,26–36^ fundamental mechanistic questions remain unresolved regarding this essential ET step. For example, does the ET occur adiabatically on a single excited state, or does it involve nonadiabatic relaxation from higher-lying electronic states? What is the role of the protein environment in ET kinetics? Addressing these fundamental questions could offer a valuable perspective for interpreting time-resolved spectroscopy experiments^25,37^ and advancing the field of photobiology in general. Simulations that quantify the thermodynamics and dynamics of ET are necessary for answering these questions. Previous studies on cryptochromes using well-established ET models such as Marcus theory^26,28,38–40^ and optimized excited-states structures^41^ are undoubtedly successful at understanding the ET mechanism in cryptochromes, but they also have limitations. For example, the assumption of equilibrium statistical mechanics is often questionable in the regime of ultrafast ET in biomolecules where nonergodic effects are prominent,^42–46^ such as in the case of cryptochromes. Moreover, these models lack the atomic-level details of real-time ET dynamics in proteins. In this regard, all-atom dynamics simulations are indispensable to complement traditional ET models because they directly propagate the coupled motions of nuclei and electrons without introducing assumptions such as ergodicity and (non)­adiabaticity.^38–40^

However, it is very challenging to perform all-atom direct dynamics simulations of the ET process in the excited-states manifold of proteins. This is mainly due to the high cost of dynamics simulations with on-the-fly potential energy surface (PES) evaluations using accurate excited-state electronic structure methods. It is even more challenging to accurately simulate the nonadiabatic ET events involving transitions among multiple adiabatic electronic states, which necessitate the correct treatment of the coupled motions of the electronic and nuclear degrees of freedom of the biomolecular system. Although a coarse-grained semiempirical method has been applied to model the ET dynamics in cryptochrome,^27,30,47^ these studies were not focused on the first ET step from TRP to FAD, and the accuracy of the semiempirical method may need further improvements for nonadiabatic ET processes involving multiple excited states on the FAD.

To address the above-mentioned challenges, in this work, we extensively characterize the first step of the photoinduced ET mechanism in *Arabidopsis thaliana* cryptochrome 1 (*At*CRY1), employing nonadiabatic and adiabatic dynamics simulations in the quantum mechanics/molecular mechanics (QM/MM) setting, with on-the-fly multireference *ab initio* electronic structure calculations. Due to its structural availability, the *At*CRY1 has long served as a model system for studying the functional mechanism of cryptochromes.^26,48^ Our nonadiabatic dynamics simulations employed the *ab initio* multiple spawning (AIMS) algorithm^49–52^ to efficiently and accurately propagate the coupled nuclear and electronic wave functions among the singlet excited-state manifold according to the time-dependent Schrödinger’s equations. Extensive multireference electronic structure calculations were performed: the Complete-Active Space Self-Consistent Field (CASSCF)^53^ method was employed in the AIMS simulation and optimizations of critical points and reaction pathways on the excited states. The Extended Multistate Complete Active Space Second-Order Perturbation Theory (XMS-CASPT2),^54^ a highly accurate multireference *ab initio* method incorporating both static and dynamic electron correlation, was employed to characterize the energies, characters and ordering of excited states at the Franck–Condon (FC) region. The results were further corroborated by extensive excited-state QM/MM adiabatic dynamics simulations using the CASSCF method. The combination of these state-of-the-art simulation methods leads to several major new findings: (1) the nonadiabatic ET is a viable pathway to induce the ultrafast ET between FAD and W400, leading to a stable S_1_-state minimum with charge transfer (CT) character, (2) there are two distinct S_1_-state minima of local excitation (LE) character, the lower of which slows down the adiabatic ET dynamics, and (3) the protein facilitates the subsequent ET steps among tryptophan residues by stabilizing their side chains.

The discussion is organized as follows: (1) characters and ordering of excited states in the FC region; (2) nonadiabatic ET induced by S_2_→S_1_ nonradiative decay, and the discovery and characterization of the S_1_-state LE and CT minima; (3) adiabatic ET on the S_1_ state following photoexcitation in the FC region, and (4) the role of the protein environment in the ET kinetics.

## Results

### Low-Lying Singlet Excited States in the Franck–Condon Region

After photoexcitation of FAD, an electron is transferred from the W400 residue to FAD (Figure 1). This reaction occurs on the excited state of the FAD-W400 dimer. The photoexcitation initiates a π→π* transition localized on the fully oxidized FAD, i.e., [FAD*-W400]. The excited-state electronic wave function of the FAD-W400 complex is thus dominated by an intramolecular local excitation at the FAD moiety, referred to as “LE character” below. After the ET finishes, a radical pair is formed, i.e., [FAD^•‑^-W400^•+^], and the excited-state wave function is dominated by an intermolecular charge-transfer excitation, referred to as “CT character” below. Considering that the FAD forms hydrogen bonds with the W400 and the protonated D396 residues^26,41^ (Figure 1C), together they are referred to as the “FWD complex” below. The FWD complex was treated in the QM region in all QM/MM simulations.

**Figure 1:**
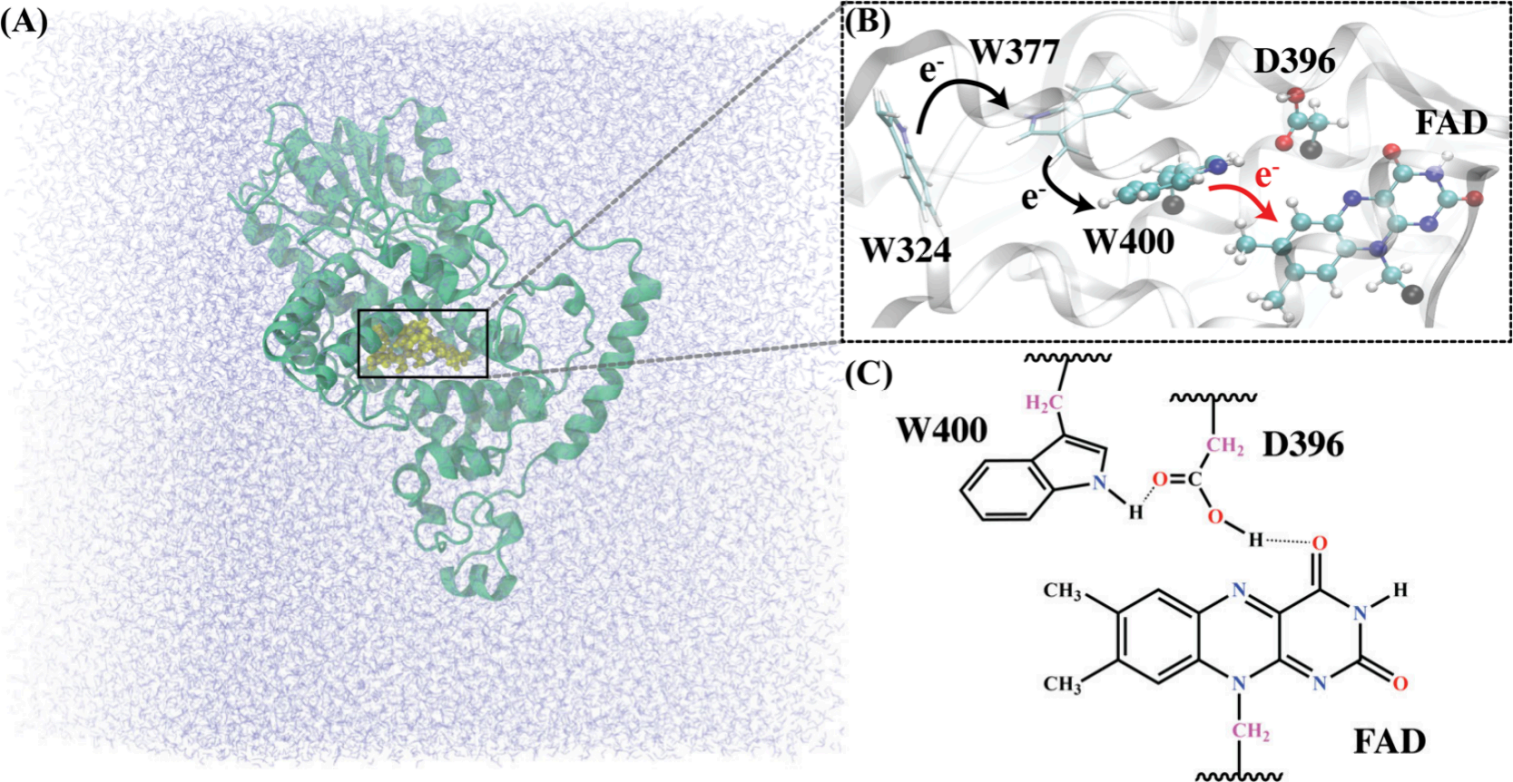
(A) Overview of the simulation box of classical MD simulations, illustrating the *At*CRY1 protein (green) solvated in water (blue). The protein backbone is shown in a ribbon representation, and the ET complex, consisting of the isoalloxazine ring of FAD, the W400, and the protonated D396 residues, i.e., the “FWD” complex, is depicted in yellow. (B) The chain of tryptophan residues (W400, W377, W324) and the FAD molecule participating in the cascade of ET events in *At*CRY1. The QM region (isoalloxazine ring of FAD, W400, and D396), which is essential for describing the initial photoinduced ET (red arrow), is depicted in a ball-and-stick representation. (C) Chemical structures of the QM region. Carbon and hydrogen atoms are shown in black, oxygen in red, and nitrogen in blue. The QM carbon atoms at the QM/MM covalent boundaries are shown in purple.

Since the energetics, characters, and ordering of the low-lying singlet states (S_1_–S_3_) can play a crucial role in the light absorption and subsequent ET dynamics, it is critical to examine them at the Franck-Condon (FC) region. We employed classical MD, ground-state QM/MM MD simulations for ground-state conformational sampling at the FC region, followed by multireference *ab initio* calculations using the XMS-CASPT2 method to characterize the excited states. This multiscale approach included the effects of the environment and the electron correlation on the excited-state properties, which are essential for accurately calculating the absorption spectra (Figure 2). The SI provides an extensive benchmark data set validating the multireference electronic structure method (Tables S2, S3, S4, S6, S7, S8) used in this approach.

**Figure 2:**
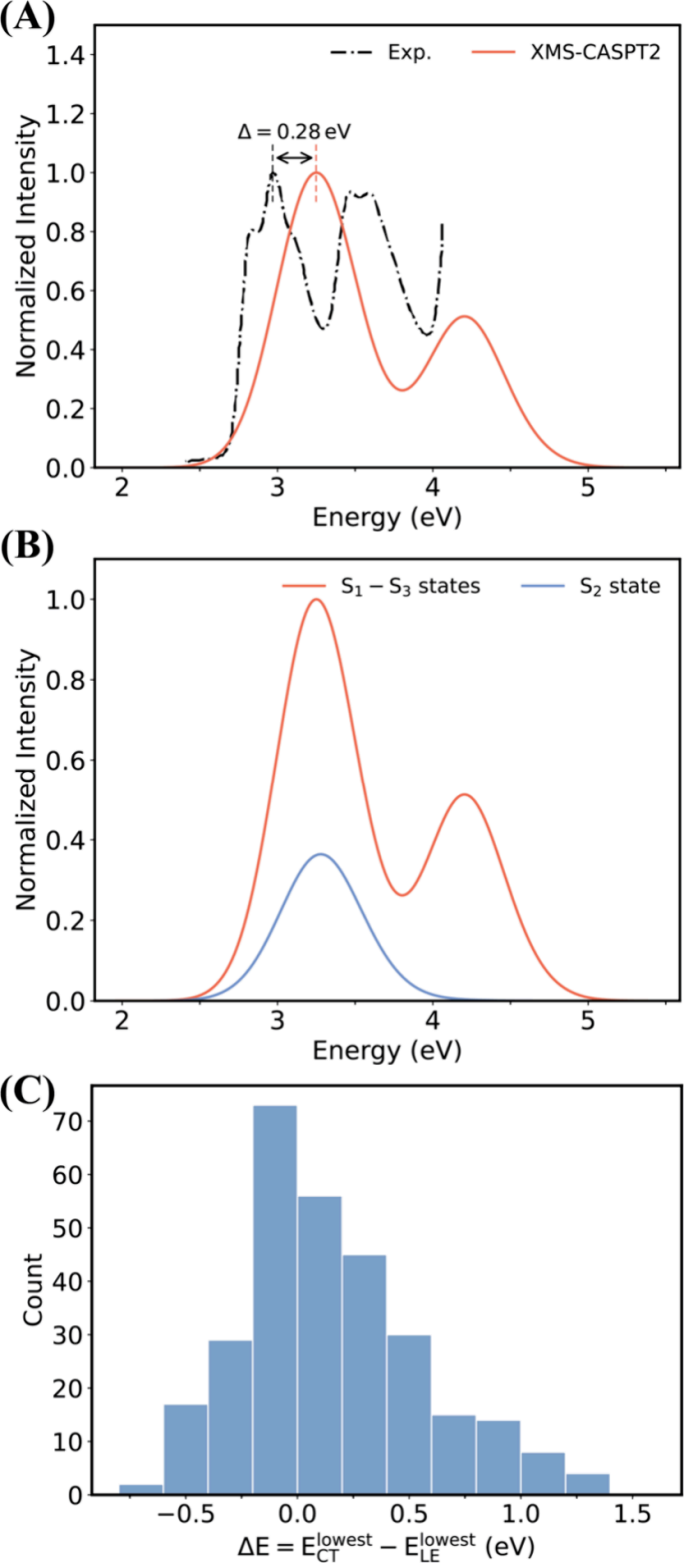
Absorption spectra and excited-state order of the FWD complex in the *At*CRY1 calculated at the XMS-CASPT2//SA-4-CASSCF­(6,6)/6–31G*/MM level of theory and compared with experiment. (A) Comparison between calculated spectrum including all excitation from to the lowest-lying excites states (S_0_→S_1_–S_3_) averaged over 300 initial conditions (ICs) sampled on the ground state in the FC region (red curve) with experimental absorption spectrum^24^ (black curve). (B) Comparison between the spectrum derived from a subset of 104 ICs (approximately 35%) whose S_2_ state has LE character and S_0_→S_2_ transition has higher oscillator strength than S_0_→S_1_ (blue curve) and all ICs (red curve). (C) Energy gap distribution between the lowest-lying singlet adiabatic excited states with the CT and LE characters in *At*CRY1, i.e., Δ*E* = *E*_*CT*_^*lowest*^–*E*_*LE*_^*lowest*^. The energy gaps were calculated for the 300 ICs in the FC region using the XMS-CASPT2//SA-4-CASSCF­(6,6)/6–31G*/MM method. Energy gaps approaching zero correspond to ICs near the conical intersections between the lowest LE and CT adiabatic states, which is critical for mediating nonadiabatic transitions between them. Negative energy gaps indicate the possibility of photoexcitation to bright LE adiabatic states higher than the CT states, potentially inducing nonadiabatic ET events.

The experimental absorption spectrum of FAD in *At*CRY1 (Figure 2A, black curve) exhibits two prominent peaks in the range of 2.5 to 4 eV.^21,23,24^ The maximum absorption wavelength was attributed to local π→π* electronic transitions at ∼2.97 eV. Our QM/MM vertical excitation calculations at the XMS-CASPT2//SA-4-CASSCF­(6,6)/6–31G*/MM level of theory reproduced the main spectral features reasonably well, yielding a maximum absorption at 3.25 eV. There is a systematic blue shift of ∼0.28 eV compared to the experimental results. This blue shift with respect to the experimental spectra is on par with earlier computational studies using TD-DFT.^26,48,55^ Figure S2 illustrates the correlation between the S_0_-S_1_ energy gap, S_0_→S_1_ oscillator strength (*f*), and S_1_-state dipole moment (Debye) for the FWD complex embedded in the *At*CRY1. The S_1_ states with LE character on the FAD exhibit high oscillator strength and low dipole moments (0–20 D), whereas those with CT character involve intermolecular ET from W400 to the FAD moiety, and they exhibit near-zero oscillator strength and high dipole moments (>25 D).

In a previous computational study by Cailliez et al.,^26^ similar blue shifts in the absorption wavelength with respect to the experiment were observed using TD-DFT with the ωB97X-D functional. We anticipate that using a more extended basis set and employing polarizable embedding in the XMS-CASPT2/MM calculations would likely lead to a red shift in the excitation energies in better agreement with the experiment.^55^ The effects of enlarging the basis set on the XMS-CASPT2 results are benchmarked in Table S8. Importantly, this previous study^26^ predicted that the lowest CT state can have lower energy than the lowest LE state in the FC region. This study^26^ thus suggested that singlet states higher than S_1_, such as S_2_ and S_3_, can be initially populated by photoexcitation to initiate ET.

We test this possibility with an accurate multireference *ab initio* method. Specifically, we analyzed the character and ordering of the lowest-lying singlet states calculated by the XMS-CASPT2 method. Approximately 35% of the total 300 sampled ground-state conformations (Figure 2B) feature an S_2_ state that is dominated by the LE character and has a larger oscillator strength for S_0_→S_2_ than that of S_0_→S_1_ (see SI Method for the definitions of excited-state characters). Importantly, the excitation energies of these ICs are mostly in the range of the lower energy peak of the spectrum (Figure 2B, blue). This supports the possible scenario that a non-negligible portion of the initial ET originates from photoexciting the FAD to the S_2_ state, followed by nonadiabatic relaxation to the S_1_ state.

The possibility of this scenario is further corroborated by the distribution of the energy gaps between the lowest-lying adiabatic singlet excited states with dominant CT and LE characters, defined as Δ*E* = *E*_*CT*_^*lowest*^ – *E*_*LE*_^*lowest*^ (Figure 2C). The fluctuation in the sign of Δ*E* emphasizes that the order of the lowest excited states with CT and LE characters is sensitive to the ground-state geometry of the FWD complex. The distribution of Δ*E* features non-negligible frequency at near-zero values, indicating energy degeneracy between the lowest-lying adiabatic states with LE and CT characters. The negative Δ*E* values indicate a non-negligible probability that the lowest excited state has a CT character and lies below a singlet state with the LE character. Many ICs in this subset have a dominating CT character in the S_1_ state. Due to the near-zero S_0_→S_1_ oscillator strength, higher-lying bright singlet states with LE character are more likely to be populated by photoexcitation. Previous work by Barbatti et.al.^56^ has reported that incorporation of zero point energy (ZPE) broadens the distribution in the geometries and excitation energies in the absorption spectra compared to sampling according to the Boltzmann distribution at 300 K. We expect the same effects of including ZPE in our simulation system. Since the state ordering is sensitive to the geometries in the FC region, we expect that including ZPE will at least maintain, if not increase, the sampling probability of the ICs with S_1_ and S_2_ states adopting CT and LE characters, respectively.

Based on these observations, we hypothesize that starting from an S_2_ state of LE character, the system may quickly access the S_2_/S_1_ conical intersection (CI) seam, followed by nonradiative decay to the S_1_ state, with some probability of ending up in an S_1_ minimum with CT character, completing the first ET step through a nonadiabatic pathway. Below, we explicitly test this hypothesis through nonadiabatic dynamics simulations.

### Nonadiabatic ET through S_2_→S_1_ Relaxation

To simulate the initial ET step in *At*CRY1 associated with nonadiabatic S_2_→S_1_ relaxation, the Stochastic−Selection AIMS (SSAIMS) simulations were initiated from 15 initial conditions (ICs) whose S_2_ and S_1_ states were dominated by the LE and CT characters, respectively. The SSAIMS simulations were propagated with on-the-fly QM/MM evaluations of PESs of the S_0_-S_3_ states using the SA-4-CASSCF­(6,6)/6–31G*/MM method. Figure 3A illustrates the time evolutions of the populations of the adiabatic S_1_ and S_2_ states. It is evident that the S_2_→S_1_ nonradiative decay is ultrafast and mostly completed within 10 fs. The predicted S_2_ lifetime is approximately 3.54 ± 0.54 fs within the protein environment. The S_2_→S_1_ decay is mediated by the S_2_/S_1_ CI seam. The minimal energy conical intersection (MECI) of the S_2_ and S_1_ states is ∼0.2–0.6 eV lower energy than the S_2_ state energy on the S_0_-state optimized FC points, based on the five ICs we tested. XMS-CASPT2/MM benchmark calculations confirm the exergonicity of this step (Table S5), and the state character changing around the S_2_/S_1_ MECI is characterized in more detail in Figures S10 & S11. The RMSD between the FC and S_2_/S_1_ MECI for the FWD complex is small, in the range of 0.06–0.1 Å. Thus, the ultrafast S_2_→S_1_ decay is facilitated by the energetically and geometrically easy access to the S_2_/S_1_ MECI on the S_2_ state from the FC region.

**Figure 3:**
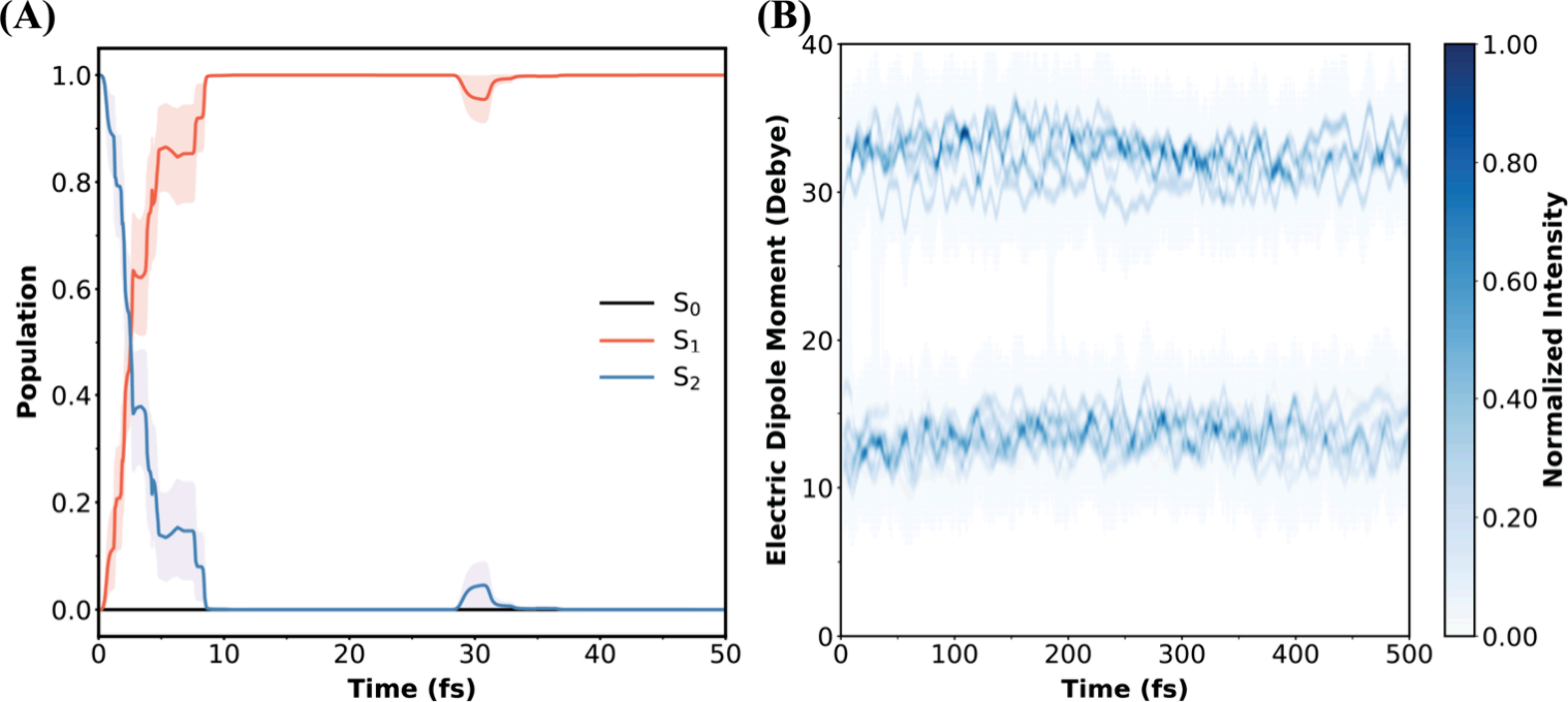
(A) The time evolution of the populations of the S_1_ and S_2_ excited states in *At*CRY1 following photoexcitation to the S_2_ state with bright LE character, extracted from the SSAIMS nonadiabatic dynamics simulations coupled with the SA-4-CASSCF­(6,6)/6–31G*/MM method. The statistical uncertainties of each curve were computed using the bootstrapping analysis with 1000 samples. (B) The time evolution of the distribution of the excited-state dipole moment (in Debye) in the SSAIMS nonadiabatic dynamics simulations. The dipole moments were analyzed from all trajectory basis functions (TBFs) during the SSAIMS dynamics. The time-dependent distribution was generated by convolving the dipole moments using fixed-width 2D Gaussians with time-dependent amplitudes of the TBFs (SI Method).

To analyze the change in the character of excited-state electronic wave functions associated with the S_2_→S_1_ decay, we tracked the distribution of excited-state dipole moments (μ) for the ensemble of trajectory basis functions (TBFs) throughout the SSAIMS simulation. The μ of each TBF residing on each adiabatic state at any given time *t* was recorded, generating a trajectory of μ­(*t*) for each TBF. Each μ­(*t*) was convolved by 2D Gaussians with widths of 1.91 D and 0.75 fs, and a time-dependent amplitude that is the same as the TBF. The convolved μ­(*t*)’s were summed up to generate the time-dependent distribution of μ in Figure 3B. This procedure averages over all independent SSAIMS runs. At any time, electronic wave functions with dipole moment less than ∼20 D are assigned as having a dominant LE character, while those above ∼25 D are assigned as a dominant CT character.

It is evident from Figure 3A that within 10 fs of the SSAIMS simulation, the TBFs with both LE and CT characters had been spawned onto the S_1_ state, and they retained their characters throughout the course of the subsequent adiabatic dynamics on the S_1_ state over a few hundreds of femtoseconds. This indicates that the TBFs are dynamically stabilized in the LE and CT minima on the S_1_ state. At the end of the SSAIMS simulation, among the 75 S_1_ TBFs, 54 TBFs were classified as LE character and 21 as CT character, leading to a quantum yield of 28% of the nonadiabatic ET event.

It is worth noting that all the 21 S_1_ TBFs having a CT character in the S_1_ state were generated by nonadiabatic transitions without any contributions from adiabatic transitions following the S_2_→S_1_ decay. Thus, the ∼28% quantum yield of ET is solely contributed by S_2_→S_1_ nonradiative decay. This is a significant new finding since it, for the first time, explicitly demonstrates the feasibility of nonadiabatic ET events in cryptochrome. Nonadiabatic relaxations from higher-lying bright states than S_2_ (not simulated here) may further increase the quantum efficiency of ET.

### Adiabatic Dynamics on S_1_ State Following S_2_→S_1_ Nonradiative Decay

The above-mentioned SSAIMS results not only elucidated the mechanism of the ultrafast nonadiabatic ET step but also revealed the existence of multiple LE and CT minima on the PES of the S_1_ state. To characterize these minima, we propagated adiabatic QM/MM trajectories on the S_1_ state for another 1 ps in the constant NVE ensemble to continue the dynamics of SSAIMS simulations. These trajectories started from the coordinates and velocities of the centroids of all S_1_ TBFs that survived at the end of the SSAIMS simulations. They carried the excess kinetic energy due to nonradiative decay from the higher-lying S_2_ state. Starting from the snapshots sampled by these adiabatic trajectories, we performed geometry optimizations to locate the LE and CT minima on the S_1_ state.

Figure S3 illustrates the time evolution of the S_1_ state’s characters of the 75 post-AIMS adiabatic S_1_ trajectories. Both the S_1_ dipole moment (in Debye) and the S_0_-S_1_ energy gaps were analyzed. The evolution of S_1_ dipole moments reveals the dynamical stability of the LE and CT minima, consistent with our SSAIMS results in Figure 3B. Dipole moment values below 20 D indicate LE character, and values above 25 D indicate CT character. The trajectories exhibiting LE characters generally exhibit larger S_0_-S_1_ energy gaps compared to those with CT characters (Figure S3). The smallest S_0_-S_1_ energy gap observed for all trajectories was ∼0.6 eV in a CT minimum, which was still too large to trigger significant S_1_→S_0_ decay even if the AIMS simulations had been run. This finding also suggests that the S_1_→S_0_ decay is most probably beyond 1 ps time scale, which will not be further investigated in this study. It is noteworthy that except for one trajectory transitioning from LE to CT character, there is no other transition events between the two characters. Thus, the trajectories largely remain in their S_1_ minima of either LE or CT character throughout the post-AIMS adiabatic dynamics.

Multiple S_1_-state minima with LE and CT characters were discovered by optimizing the snapshots randomly selected from the S_1_ adiabatic trajectories (Table S1). The electronic characters of the S_1_ state minima were assigned based on the dipole moments and the total S_1_-state Mulliken charges on the FAD moiety and W400. Two types of LE minima were observed, one with high S_0_-S_1_ excitation energies and the other with lower ones (Table S1). The CT minima mostly have lower S_0_-S_1_ excitation energy than both types of LE minima. *Importantly, the LE minimum with the lower excitation energy between 2.5–2.8 eV was not previously reported and is a key finding in this study.* Following the S_2_→S_1_ relaxation, some trajectories quickly reached the LE minimum with the higher excitation energy and were temporarily stabilized there. However, eight trajectories were found to escape this minimum during the 1 ps adiabatic dynamics and reached the LE minimum with lower excitation energy and got stabilized there (Figure S4A). Figure S4B compares the distribution of S_0_-S_1_ energy gaps of the S_1_-state TBFs after the SSAIMS simulation was completed and after 1 ps subsequent propagation on the S_1_ state. It is evident that some trajectories with energy gaps near 3.0 eV at the end of SSAIMS simulation eventually evolved into low-energy LE minima with energy gaps below 2.8 eV. Taken together, these results imply that the LE minimum with lower excitation energy is energetically lower and thermodynamically more stable than the one with higher excitation energy. As will be discussed below, this low-energy LE minimum can temporarily slow down the adiabatic ET event following photoexcitation to a bright S_1_ state.

The stability of the CT minima was further examined by initiating 15 adiabatic S_1_-state trajectories for another 0.9 ps from the optimized structures of the CT minima, starting with random velocities and propagated in the constant NVT ensemble (Figure S5). These additional simulations further confirm the dynamic stability of the CT minima, because randomly thermalized velocities ensured a statistically meaningful evaluation by mitigating biases from original ICs. As illustrated in Figure S5, the S_1_ dipole moment remains around 30 D throughout the simulation, indicating that no CT to LE transitions were observed. Furthermore, the S_0_-S_1_ gap remains around 1 eV, which agrees with previous results. These findings suggest that the CT state, once formed, remains stable at the picosecond time scale. The dynamical stability of the CT minima is particularly crucial for the function of cryptochromes because it corresponds to the formation of a stable radical pair [FAD^•**‑**^-W400^•+^]. Our results indicate that once the radical pair is formed on the S_1_ state through the nonadiabatic pathway, it can be stabilized until further ET steps downstream of the tryptophan chain, which further corroborates the viability of the nonadiabatic ET mechanism.

### Adiabatic ET Step on the S_1_ State

In the 1 ps S_1_ state adiabatic dynamics following the SSAIMS simulation, the transitions between the LE and CT minima were a rare event with less than 2% probability (Figure S3). This implies the existence of energy barriers between these minima. To estimate the magnitude of the barriers, the nudged elastic band (NEB) method was employed to optimize the minimum energy paths (MEPs) connecting the LE and CT minima on the S_1_ state at the SA-4-CASSCF­(6,6)/6–31G*/MM level of theory in the *At*CRY1 (see Methods). The PESs along the MEPs are displayed in Figure 4. Starting from the low-energy LE minimum, a large energy barrier (∼6 kcal/mol) needs to be overcome to reach the high-energy LE minimum (Figure 4A). Starting from the high-energy LE minimum, there is a smaller energy barrier of ∼0.6 kcal/mol to be overcome to reach the CT minimum (Figure 4B), and the CT→LE barrier of backward ET reaction is higher than 5 kcal/mol (Figure 4B).

**Figure 4:**
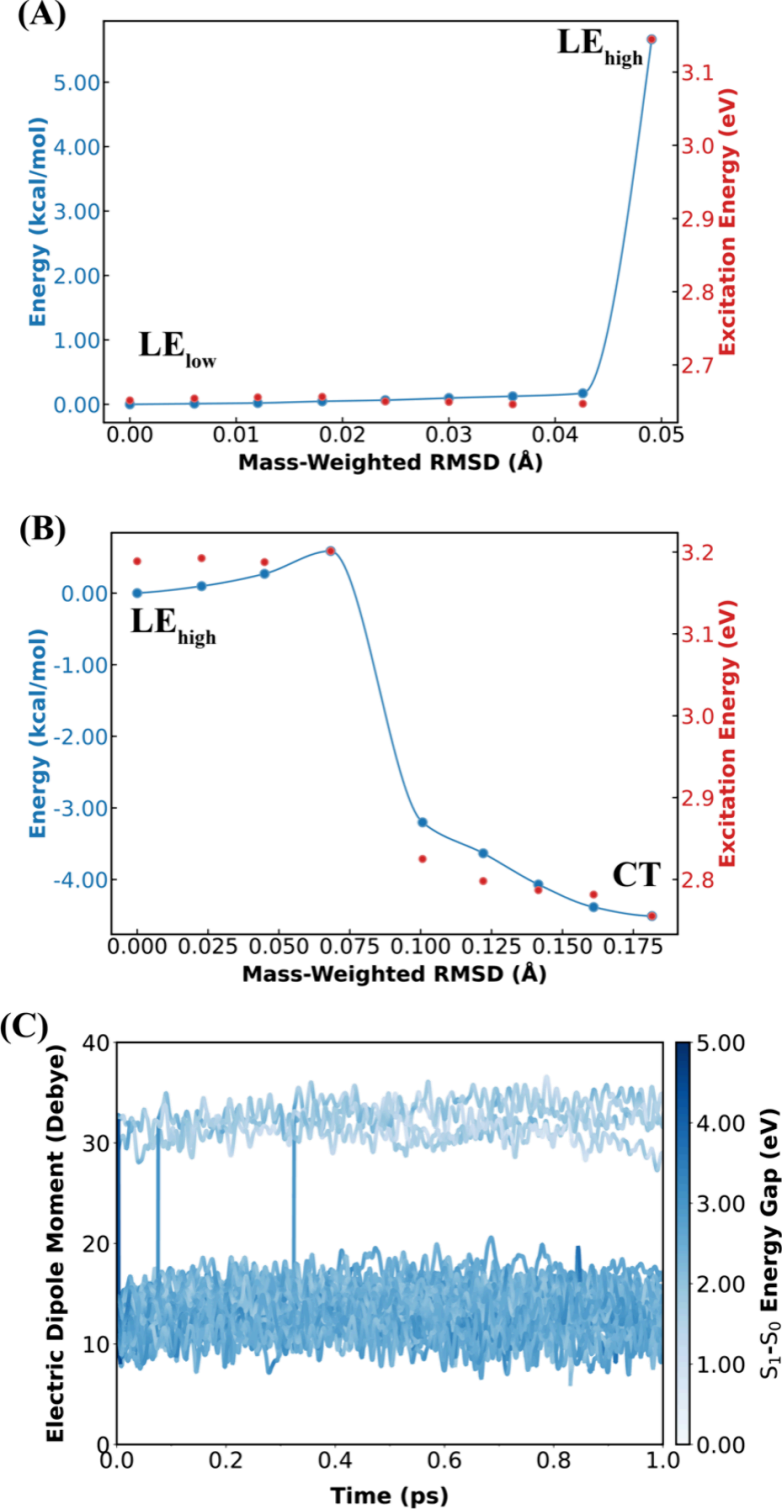
(A) Minimum energy pathways (MEPs) on the S_1_ state connecting the low-energy to the high-energy LE minima (labeled as LE_low_ and LE_high_, respectively). (B) MEP on the S_1_ state connecting the high-energy LE minimum to the CT minimum. The MEPs are shown as blue curves and dots. The S_1_–S_0_ energy gaps of each image along the MEPs are shown as red dots. The MEPs were optimized using the NEB method at SA-4-CASSCF­(6,6)/6–31G*/MM level of theory. (C) Time evolution of the S_1_ dipole moment of 50 S_1_ adiabatic trajectories starting from the ICs sampled in the FC region featuring a bright S_1_ state with LE character. The adiabatic dynamics were propagated in the constant NVE ensemble. The shade of the color represents the S_1_–S_0_ energy gap. The majority of the trajectories (∼92%) are stabilized in the S_1_-state LE minima visited soon after the photoexcitation to the S_1_ state. The S_1_–S_0_ energy gaps oscillate between 0.66 eV and a maximum value of 4.80 eV throughout the simulation. All on-the-fly QM/MM calculations were carried out at SA-4-CASSCF­(6,6)/6–31G*/MM level of theory.

These results confirm our above-mentioned finding that the LE minima with the lower S_0_-S_1_ gap also have a lower energy on the S_1_-state PES. The relative stability of the low- and high- energy LE minima are consistent with the observation that the post-AIMS S_1_ adiabatic trajectories exhibit transitions from the high-energy to the low-energy LE minima.

The total estimated barrier for the entire adiabatic transformation from the low-energy LE minima to the CT minima is ∼6 kcal/mol. This barrier, after single-point energy corrections at XMS-CASPT2/SA-4-CASSCF­(8,8) level of theory, is reduced to ∼3.5 kcal/mol **(** Figure S8 & S9 **)**. Considering nuclear quantum effects such as ZPE and tunneling, as well as further possible corrections to the PES by more accurately incorporating dynamic electron correlation, this adiabatic barrier from the low-energy LE to CT minima is consistent with an experimental rate of 0.4 ps^37^, which is faster than the second ET steps from the W377 to the W300, which occurs in the range of 4–15 ps^37^.

The structures of the FWD complex corresponding to the MEP end points and highest images are shown in Figure S6. Notably, the structural similarities among these structures indicate that the character of the excited states is sensitive to the molecular geometry, particularly at the isoalloxazine ring of the FAD.

The above MEP analysis suggested that adiabatic ET events can be slowed down by the low-energy LE minima if the photoexcitation directly populates the bright S_1_ state with LE character. To test this hypothesis, we initiated adiabatic S_1_-state dynamics from the ICs in the FC region having a bright S_1_ state with LE character, which is dominant in the set of all sampled ICs (Figure 2). Figure 4C illustrates the evolution of the S_1_ dipole moment of 50 ICs. Only four trajectories (∼8% of total trajectories) underwent the adiabatic transition from the LE to the CT minima. The lowest S_0_-S_1_ gap observed among all trajectories is 0.66 eV, which is still sufficiently large to avoid nonadiabatic decay to the S_0_ state even if the AIMS simulations had been performed. Taken together, these observations suggest that many S_1_ state trajectories are temporarily stabilized in the low-energy LE minima, slowing down the access to the CT minima, in agreement with our above-mentioned MEP analysis.

These findings indicate that the initial photoinduced ET can occur adiabatically on the S_1_ state following photoexcitation to the S_1_ state, resulting in the formation of the radical pair. However, the S_1_-state energy barrier may slow this process. We note that including dynamic correlation in the electronic structure method can reduce the adiabatic barrier, and increase the calculated rate of the adiabatic ET events to be in better agreement with the 0.4 ps time constant measured by the experiment.^37^ However, the nonadiabatic ET provides an alternative ultrafast route to complement the adiabatic one, facilitating radical pair formation. This ultrafast nonadiabatic ET dynamics finishing within 10 fs after photoexcitation is beyond the current time resolution of transient absorption spectroscopy. Additionally, thermal fluctuations on the ground state are essential for nonadiabatic ET events by means of changing the state order in the FC region.

### Influence of the Protein Environment

It is well-known that the molecular environment has significant effects in modulating photochemical reactions.^57–66^ In proteins, these effects usually arise from the electrostatic potential created by the hydrophilic residues and the steric restrictions in the binding pocket of the chromophore.^57–59,61,63^ For *At*CRY1, the spatial arrangement of the FAD and the tryptophan triad is particularly important for the successful production of a long-distance separated radical pair through a cascade of ET events since it influences the overlap of molecular orbitals between donors and acceptors. Also, the electrostatics created by the protein can also play essential roles since it can change the relative stability of LE and CT minima of excited states, as well as the energy barriers connecting them.

To assess the influence of the electrostatics on the characters and relative energies of the S_1_ minima, we performed constrained QM/MM optimization on the S_1_ state, starting from 63 ICs in the sampled in the post-AIMS S_1_-state adiabatic trajectories. Only the active region, i.e., the FWD complex (Figures 1B&C), was allowed to relax, while the rest of the system was fixed. After constrained optimization, the FWD complex was extracted from the protein matrix, and a single-point energy calculation was performed in the vacuum (see SI Method). Figure S7 illustrates the distributions of the S_1_–S_0_ energy gap and the character of the S_1_ state calculated in both environments using identical geometries of the FWD complex. It highlights the substantial differences between these two environments. In the protein environment, the S_0_-S_1_ energy gaps exhibit a bimodal distribution, with peaks at approximately 2.7 and 3.1 eV (Figure S7A **)**. Most of the sampled structures (46 out of 63) have an S_1_ state with LE character. Conversely, in the vacuum, the S_0_-S_1_ energy gap distribution is centered around 2.7 eV but displays a broader spread in the lower-energy range (Figure S7B). This shift correlates with a significant increase in the frequency of the CT character, found in 61 out of 63 geometries, while only two geometries exhibit LE character. The data suggests that protein electrostatics favors local excitation on the FAD. *It is an interesting result since the protein electrostatics usually facilitate the catalyzed reaction, such as in enzymatic catalysis, instead of hindering it.*

Additional new insights were obtained from optimizing the FWD complex in the vacuum on the S_1_ state under different conditions: (1) the atoms previously at the QM/MM boundary were fixed to the corresponding positions in the protein, and (2) full relaxation of all atoms. The results are presented in Figures 5A&B. In total, 28 structures were optimized in both conditions. Optimized structures with positional constraints yielded both LE and CT minima on the S_1_ state. In contrast, the fully relaxed optimizations yielded only CT minima. The removal of constraints allows the FAD and W400 to change their relative orientations with respect to each other, thus lowering the energy of the charge transfer minima. The Figures 5C&D presents the structures of FWD optimized under different conditions, comparing the structures in the protein environment and the vacuum, with and without structural constraints from the protein, respectively.

**Figure 5:**
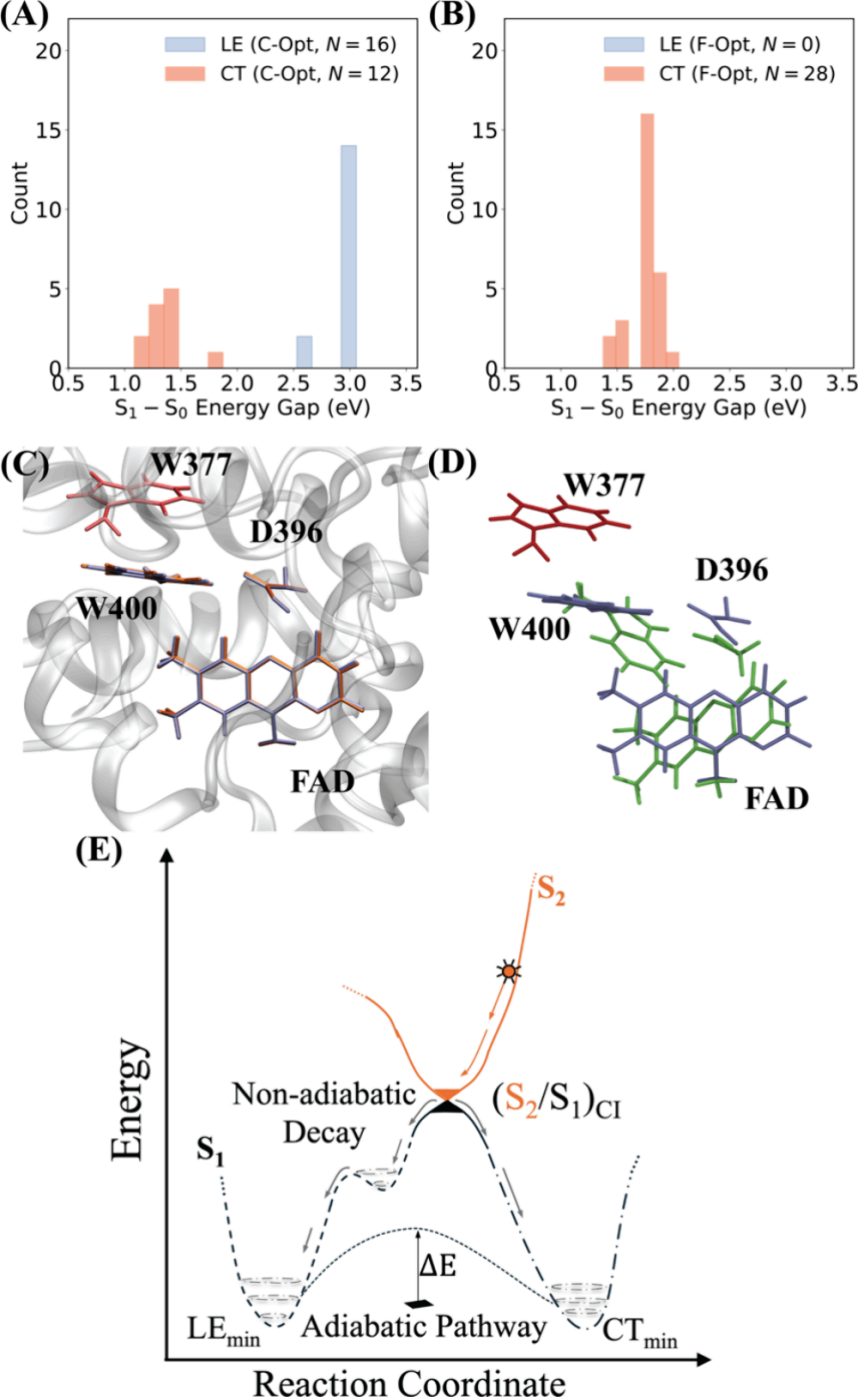
(A) Distributions of S_0_-S_1_ energy gaps and characters of the S_1_ state of the FWD complex after (A) constrained optimization (C-Opt) and (B) free optimization (F-Opt) in the vacuum on the S_1_ state at the SA-4-CASSCF­(6,6)/6–31G* level of theory. The data set comprises 28 distinct optimized structures in the vacuum starting from sampled snapshots in the S_1_-state adiabatic dynamics in protein. (C) Structural representation of the active region in the *At*CRY1 binding pocket. The S_1_-state LE and CT minima are colored in blue and orange, respectively, for the FWD complex. The W377 residue lying downstream in the ET cascade from W400 is colored in red. (D) Comparison between the FWD’s structures in the CT minima optimized in the protein environment (blue) and in the vacuum (green), highlighting the effects of the binding pocket in maintaining the relative orientation between the W400 and W377 residues. The geometries were aligned at the terminal methyl group of the flavin moiety (depicted in Figure 1). (E) Schematic representation of the nonadiabatic and adiabatic ET mechanisms in the *At*CRY1. The nonradiative mechanism mediated via S_2_/S_1_ CI is depicted at the top, with the orange curve representing the S_2_-state PES. The two LE minima on the S_1_-state PES are illustrated by the dashed black curve. The dotted-dashed line represents the CT minimum. The adiabatic ET pathway connecting the LE and CT minima is shown as a fine-dotted curve with the adiabatic energy barrier indicated as Δ*E*.

Overall, our qualitative analysis based on single-point energy calculations and local geometry optimizations does not directly reveal how the protein environment facilitates the transition from the LE minimum to the CT minimum on the S_1_ state. However, we reason that without the geometric constraints imposed by the protein, random fluctuations in the orientation and distance between the donor and acceptor can impede the ET. In this way, the protein environment plays a crucial role in the initial photoinduced ET step by limiting the conformational flexibility of the FAD-W400 pair. Additionally, the nonadiabatic ET pathway, which is sensitive to conformational sampling near the Franck–Condon region (see above), also benefits from these constraints. By restricting thermal fluctuations within a narrowly distributed structural ensemble, the protein effectively positions the structures of the FAD and W400 near the S_2_/S_1_ conical intersection seam, and also allows direct photoexcitation to the S_2_ state with LE character to initiate the nonadiabatic ET dynamics.

The above analysis also raises an interesting question: how can the *At*CRY1 facilitate the propagation of the [FAD^•‑^-W400^•+^] radical pair through the tryptophan triad? We reason that the constraints imposed by the protein environment may ensure the correct relative orientation between W400 and its neighboring W377 that maximizes their overlap in molecular orbitals, resulting in optimal diabatic coupling to facilitate the next step of the ET from W400 to W377. In Figures 5C&D, we illustrate how the protein restricts and stabilizes orientations of the FWD complex with respect to the W377, compared to the optimized structures of FWD in the vacuum. Thus, we hypothesize that the protein environment can speed up the subsequent ET steps between the tryptophan residues. This hypothesis needs to be tested in future work using Marcus’s theory in the vacuum and protein environments for subsequent ET steps.

## Discussions and Conclusions

In this work, the mechanism of the initial step of photoinduced ET in *At*CRY1 was systematically characterized by extensive nonadiabatic and adiabatic dynamics simulations with multireference *ab initio* QM/MM calculations. The key new findings are summarized as follows.

First, ET from the W400 residue to the FAD can proceed through the ultrafast S_2_→S_1_ nonradiative decay within a few tens of femtoseconds, which is complementary to the slower adiabatic ET on the S_1_ state. The nonradiative ET pathway is physically meaningful and relevant due to the noticeable amount of conformations in the FC region that has a bright S_2_ state. Its significance also arises from the large portion of the S_1_ population dynamically stabilized in the CT minima after the S_2_→S_1_ decay. To the best of our knowledge, this new pathway has not been previously investigated using computational modeling as rigorous as this work. A previous experimental study by Immeln et al.^37^ on *At*CRY1 reported an initial photoinduced ET time constant of approximately 0.4 ps, and similar rates (0.5–0.8 ps) have been observed in photolyases^67,68^ and robin cryptochrome 4.^25^ Our simulations predict that the nonadiabatic ET event occurs within ∼10 fs (Figure 3A), which is significantly faster than these experimental time constants. In contrast, our XMS-CASPT2 calculations indicate an S_1_-state barrier of about 3.5 kcal/mol for the adiabatic ET process (Figures S8 & S9), which, after accounting for nuclear quantum effects such as tunneling and zero-point energy as well as further corrections to the PES, can align well with the experimentally observed time scales. Importantly, transient absorption spectroscopic measurements typically resolve dynamics above ∼25 fs, and the ultrafast nonadiabatic ET event is beyond this resolution limit. Moreover, as shown in Figure 2B, due to conformational fluctuations in the Franck–Condon region, the excitation energies for S_0_→S_2_ transitions (associated with nonadiabatic ET) overlap with those for S_0_→S_1_ transitions (leading to adiabatic ET), making it difficult to disentangle the contributions of each pathway at the ∼445 nm excitation wavelength. Therefore, our findings support that the time constants measured experimentally correspond primarily to the adiabatic ET on the S_1_ state, while the rapid nonadiabatic ET remains consistent with, yet unresolved by, current spectroscopic methods.

Second, two types of LE minima on the S_1_ state were discovered following the nonadiabatic relaxation, and the low-energy LE minimum can slow down the adiabatic ET. The high-energy LE minimum was previously identified by geometry optimizations at the CASSCF level of theory with a smaller active space than this study^41^ without dynamics simulation. It was considered the only LE minimum before reaching the CT minimum in the adiabatic pathway. In this work, however, through extensive S_1_-state CASSCF QM/MM adiabatic dynamics with a larger active space, both following the S_2_→S_1_ decay and starting from FC region, we show that the high-energy LE minimum is metastable, and it can quickly relax to the newly identified low-energy LE minimum (Figure S4). The low-energy LE minimum stabilizes the LE character and thus slows down the adiabatic ET event. This new discovery is significant because it deepens our understanding of the key features of PES on the S_1_ state that influence the kinetics of ET.

Third, the CT minima on the S_1_ state visited after the nonradiative decay remains dynamically stable on the picosecond time scale. Even with the excess kinetic energy after the decay, the CT minimum is stable enough to prevent backward transitions to the LE minimum, which would have eliminated the newly generated radical pair. Also, the stable CT minima can better prepare the system for the next ET step from the W400 to the W377. Noticeably, during all trajectory dynamics, the S_1_ and S_0_ states were never close to being degenerate. This suggested that the S_1_→S_0_ decay may take place in a longer time scale. Future studies will be focused on this aspect.

Interestingly, our results indicate that the ultrafast, nonadiabatic ET in cryptochrome can reduce the thermal noise associated with an S_1_-state adiabatic barrier crossing. This is analogous to rhodopsins, where the high activation barrier for thermal *cis*-to-*trans* isomerization inherently minimizes dark noise and ensures that photoisomerization dominates.^69–74^ In other words, the cryptochrome can leverage the higher-lying LE states and reach the CT minima on the S_1_ state through the nonadiabatic pathway, which is faster than through the adiabatic pathway. However, unlike rhodopsins where thermal noises are nearly eliminated, the S_1_ adiabatic ET in cryptochrome is still non-negligible due to the significant population of conformations being photoexcited to the S_1_ state with LE character (Figure 2). Also, our benchmarks (Figures S8 & S9) show that energy corrections via incorporating dynamic correlations at XMS-CASPT2 level of theory slightly lower the adiabatic S_1_-state barrier predicted by the SA-CASSCF approach. This result, together with experimental observations of subpicosecond to picosecond ET rates^25,37,67,68^ of cryptochromes and photolyases, supports a model where ultrafast nonadiabatic ET facilitates charge separation while not entirely suppressing thermal noise. Despite this quantitative difference, the mechanism underlying thermal noise suppression in *At*CRY1 remains qualitatively the same as the one observed in rhodopsins.^73,74^

Last but not least, the electrostatic environment created by the protein stabilize the LE minimum more than the CT minimum on the S_1_ state, seemingly disfavoring the initial ET event. However, the arrangements of the side chains of the tryptophan residues and the FAD in the protein could ensure good overlap in the molecular orbitals between them, thus enabling quick ET events to occur both nonadiabatically and adiabatically. Without the protein’s steric constraints, large reorientation of the tryptophan residues and the FAD can make ET difficult. *Thus, the protein environment facilitates the kinetics of different ET steps by steric constraints to maximize the overall quantum efficiency.* This new interpretation of the role of protein on the ET in cryptochromes deepens our understanding of photoreactions in biomolecules.

In Figure 5E, we schematically summarize our new findings regarding the pathways of nonadiabatic and adiabatic ET in *At*CRY1. In conclusion, through comprehensive and accurate computational characterizations of different reaction pathways, our study complements the existing picture of the ET in light-sensing cryptochromes, deepening the fundamental understanding of the initial ET step in them. Our findings highlight the intricate interplay among molecular geometry, excited-state characters, and ET dynamics. As such, our study contributes to the broader field of photochemistry and photobiology by elucidating molecular mechanisms that govern light-induced charge transfer events in photoreceptors.

## Summary of Computational Methods

Detailed computational methods are provided in the SI.

The system setup was initiated from the crystal structure of *At*CRY1 (PDB code: 1U3C),^75^ with missing terminal residues rebuilt using the MODELLER software package^76^ and protonation states assigned at neutral pH (the D396 was assigned as protonated to favor photoinduced electron transfer^26,41^ ) using the H++ server.^77^ The protein, along with crystallographic water molecules and Mg^2+^ ions, was solvated in a periodic box, modeled with the Amber ff14SB force field and the SPC/Fw water model,^78^ while the FAD chromophore was parametrized via general AMBER force field (GAFF) procedure^79,80^ in its fully oxidized, dark-adapted state. Initial classical MD simulations involved restrained energy minimization, gradual heating and relaxation of restraints, followed by a 10 ns production run in the constant NPT ensemble at 300 K temperature and 1 atm pressure.

Subsequently, ground-state QM/MM MD equilibration was performed on 20 configurations sampled from the classical MD trajectory with 0.5 ns time interval in order to refine the equilibrium structures of the FWD complex in the FC region. In these simulations, the QM region (Figures 1B&C) was treated with DFT using the ωPBEh functional^81,82^ and a 6–31G* basis set,^83,84^ while the MM region maintained the classical force field description. The QM and MM regions were coupled through electrostatic embedding. For excited-state calculations in the FC region, vertical excitation energies and oscillator strengths were calculated for 300 configurations sampled from ground-state QM/MM MD simulation, using the XMS-CASPT2//SA(4)-CASSCF­(6,6)/6–31G*/MM approach.^54,85^ The excited states were assigned as either LE or CT character based on Mulliken charges and dipole moments.

The photoinduced electron transfer process was further examined through nonadiabatic dynamics using the Stochastic-Selection AIMS (SSAIMS) method, initiated from selected bright S_2_ states to probe S_2_→S_1_ decay starting from 15 ICs. The SSAIMS^52,86^ is a variant of the original deterministic *Ab Initio* Multiple Spawning (AIMS) algorithm. It introduces a stochastic selection procedure to reduce the number of TBFs being simultaneously propagated and, consequently, significantly saves computational costs. At each time step, the method evaluates the coupling between two TBFs and identifies groups that have become effectively decoupled based on a predefined decoupling threshold. A stochastic procedure is then used to select, based on the total population of each uncoupled group, one of the uncoupled groups to continue the propagation while the others are terminated. This strategy controls the growth in the number of TBFs while preserving the essential dynamical details described by the reference AIMS simulations.^52,86^ Benchmark studies^52,86^ have shown that defining the decoupling threshold based on TBF overlaps (referred to as OSSAIMS) best agrees with the deterministic reference AIMS dynamics for the smallest average number of TBFs, so this implementation was employed here. Due to stochastic selections of TBFs, multiple independent SSAIMS simulations need to be launched from each IC using different random seeds such that the results statistically converge to the reference AIMS simulations. We performed 5 independent runs for each IC. The SSAIMS simulations, conducted with on-the-fly SA-4-CASSCF­(6,6)/6–31G*/MM calculations, were extended up to 500 fs and averaged over 5 runs for each IC to capture state populations and charge evolutions.

Following the SSAIMS simulations, 75 S_1_-state adiabatic dynamics simulations were launched, each restarting from the coordinates and velocities of an individual S_1_ TBF’s centroid that survived at the end of the SSAIMS simulations. The S_1_-state trajectories were propagated in the constant NVE ensemble to ensure the continuation of the dynamics from SSAIMS simulations. Additionally, 15 S_1_-state dynamics simulations were launched from the optimized CT minima with random velocity and propagated in the constant NVT at 300 K ensemble to test the stability of the CT minima. Furthermore, 50 S_1_-state adiabatic dynamics simulations were launched on the S_1_ state in the FC region, starting from ICs with bright S_1_ state of LE character, in order to simulate the direct dynamics of adiabatic ET following photoexcitation to the S_1_ state. Excited-state geometry and reaction pathway optimizations, including constrained geometry optimizations and nudged elastic band calculations, were performed to delineate the energy landscape connecting S_1_-state LE and CT minima. Geometry optimizations in the vacuum with and without positional constraints were also carried out to elucidate the protein’s effect on the ET mechanism.

All classical MD simulations were executed using the GPU-accelerated version of the AMBER software package (v. 20).^87^ Ground-state QM/MM MD simulations were performed with the TeraChem^88–91^ software package interfaced with the OpenMM^92^ package. QM/MM calculations of the absorption spectra were performed using the OpenMolcas^93^ interfaced with the Tinker software packages.^94^ All SSAIMS were propagated using the FMS90 code interfaced with the TeraChem/OpenMM packages. All excited-state geometry optimizations and MEP optimizations were carried out using the TeraChem package. Detailed benchmark data in the FC region, along the S_1_-state MEP and at the S_2_/S_1_ MECI is included in the SI.

## Supplementary Material





## Notes

Detailed computational method and Figures S1–S11, including the active space of the CASSCF method, the characterization of ICs in terms of oscillator strength, the LE/CT character, and S_0_-S_1_ excitation energies, the analysis of post-AIMS adiabatic trajectories stabilized in the LE and CT minima, the analysis of adiabatic trajectories undergoing transition from LE_high_ to LE_low_ S_1_-state minima, the critical points along the MEP on the S_1_ state, the effects of electrostatics on the distribution of LE and CT characters of the S_1_ state, the benchmark calculations along the S_1_-state MEP using the XMS-CASPT2 method, and S_2_/S_1_ MECI characterization. In addition, Table S1 illustrates the photophysical properties of the representative structures of the three types of S_1_-state minima. Tables S2–S8 include benchmark calculations in the FC region and S_2_/S_1_ MECI (PDF)
Transparent Peer Review report available (PDF)
