# Supplementary material for: Decrypting the Nonadiabatic Photoinduced Electron Transfer Mechanism in Light-Sensing Cryptochrome
Source: ACS Cent Sci. 2025 May 30;11(7):1071–82. doi: 10.1021/acscentsci.5c00376 (PMC12291142; doi:10.1021/acscentsci.5c00376)
Supplement: Supplementary file 1 [file oc5c00376_si_001.pdf]

Supplemental information for

Decrypting the Nonadiabatic Photoinduced Electron Transfer Mechanism in Light-Sensing Cryptochrome

Gustavo J. Costa and Ruibin Liang*

Department of Chemistry and Biochemistry, Texas Tech University, Lubbock, TX, 79409, USA

AUTHOR INFORMATION

Corresponding Author

*Ruibin Liang

Email address: rliang@ttu.edu

Table of contents

Computational method **3**

System setup 3

Classical MD equilibration simulations 3

Ground-state QM/MM MD equilibration simulations 4

Absorption spectra calculation and the analysis of the excited states' ordering 6

Nonadiabatic dynamics simulations 7

Excited-state QM/MM adiabatic dynamics simulation 9

Optimization of critical points and reaction pathways on the excited states 10

Key figures and tables **12**

Table S1 12

Figure S1 13

Figure S2 14

Figure S3 15

Figure S4 16

Figure S5 17

Figure S6 18

Figure S7 19

Benchmark calculations **20**

Ground-state conformations in the Franck-Condon region 20

Table S2. 21

Table S3. 23

Table S4 24

Excited-state conformations from Franck-Condon regions 25

Figure S8 26

Figure S9 26

Table S5. 27

Consistency between ground-state sampling and nonadiabatic dynamics 28

Table S6 29

Table S7 29

Effects of Basis set 30

Table S8 30

Characterization of the S_2/S_1 MECI 31

Figure S10 33

Figure S11 34

Computational method

System setup

The system setup was initiated from the crystal structure of *At*CRY1 (PDB code: 1U3C).[1] The coordinates of the residues at the N- and C-termini of the protein's sequence, which were missing from the PDB, were reconstructed using MODELLER software.[2] Hydrogen atoms were added according to the protonation states of the residues at neutral pH (7.0) predicted by the H++ server[3], except for the D396 residue, which was treated as protonated due to its favorable effects on the photoinduced ET.[4, 5] All crystallographic water molecules and Mg^{2+} ions in the crystal structure were retained, while all other non-essential components were removed.[5] Then, the protein was solvated by a box of water molecules with 150 mM concentration of NaCl. The resulting system has a periodic boundary condition of approximately 104 Å × 112 Å × 102 Å, including 120,924 atoms in total (**Fig. 1A**).

The protein was modeled using the Amber ff14SB force field (FF)[6], while water molecules were represented by the flexible SPC/Fw water model.[7] The FAD chromophore was parameterized using the General Amber Force Field (GAFF) procedure.[8] Since this study centers on the mechanism of the first photoinduced ET step, the FAD was parameterized in its fully oxidized conformation and at the S_0 ground state (dark-adapted state). The system setup was performed using the "Tleap" program from the AmberTools20 software package[9].

Classical MD equilibration simulations

First, geometry optimization using force field was performed to reduce steric clashes and unfavorable interactions within the system. During the optimization, all heavy atoms of protein and FAD were restrained to their crystallographic coordinates using harmonic potentials with force constants of 100 kcal/mol/Å^2. Following minimization, the system underwent force-field-based molecular dynamics (MD)

equilibration in multiple stages, gradually releasing the positional restraints. The system was gradually heated from 100 K to 300 K under constant volume, during which the force constants of the positional restraints were reduced from 100 to 75 kcal/mol/Å². The equilibration continued for 6 ns in the constant NPT ensemble at 300 K temperature and 1 atm pressure, during which the harmonic restraints on both the protein and FAD ligand were gradually reduced from 50 kcal/mol·Å² to 0.5 kcal/mol·Å². After equilibration, production simulation in the constant NPT ensemble at the same temperature and pressure was performed for 10 ns with all positional restraints completely removed. The production simulation was designed to be relatively short to maximally preserve backbone's crystal structure of protein while allowing for relaxation of the chromophores, cofactors, protein side chains, and solvent molecules.

The atom-MD simulations were conducted with a 1 fs time step and a 12 Å cutoff for van der Waals interactions, while the Particle Mesh Ewald (PME) method[10] was used to treat the electrostatic interactions under the periodic boundary condition (PBC). The temperature was maintained using a Langevin thermostat with a collision frequency of 1 ps^{-1}. The pressure was regulated using the Berendsen barostat with a relaxation time constant of 1.0 ps. All MD simulations were executed using the GPU-accelerated version of the AMBER software package (v. 20).[11]

Ground-state QM/MM MD equilibration simulations

Ground-state (the S_0 state) adiabatic simulations at the QM/MM level of theory were conducted starting from the molecular conformations sampled from the production trajectory of classical MD simulation. This QM/MM MD equilibration step was designed to correct for inaccurate descriptions of the geometries of the FAD chromophore and surrounding residues (W400 and D396) that may have arisen from the force-field-based MD simulations. A total of 20 configurations with 0.5 ns time intervals were chosen from the

10 ns classical MD trajectory as the initial conditions (ICs) for ground-state QM/MM MD equilibration. For each configuration, a subsystem with an open boundary condition was built from the PBC system. All molecules with any atoms within a 12 Å radius of any atom of the protein or FAD moiety were selected to construct the subsystem, which included the entire protein, the FAD, all cofactors of the *At*CRY1, and all nearby water molecules, whereas the remaining atoms were discarded. The setup of the open-boundary subsystem was due to the limited capability of the production version of TeraChem we utilized in this work, which at the time did not fully support excited-state QM/MM dynamics simulations under PBC.[12] The subsystem was designed to maintain an accurate representation of the local environment around the reaction center at the FAD molecule.[13]

The QM region included the isoalloxazine ring of the FAD chromophore and the W400 and D396 residues[14] (**Fig. 1B&C**). They are critical players in the initial photoinduced ET.[5] The MM region included the rest of the system. The QM and MM regions were coupled through electrostatic embedding, where the QM atoms' electronic wavefunctions responded to the presence of fixed point charges on the MM atoms. The QM/MM boundary across covalent bonds was treated using the hydrogen link atom approach.[15] The QM region was treated with density functional theory (DFT), employing the range-separated hybrid density functional ωPBEh[16] ($\omega = 0.2$) and the 6-31G* atomic basis set.[17] The remainder of the system, including the protein, water, and ions, was modeled using classical molecular dynamics force field parameters as previously described.

All 20 adiabatic QM/MM MD simulations on the ground state were performed for ~5 ps at 300 K temperature, utilizing an integration time step of 0.5 fs. The initial 1 ps was discarded as equilibration, and the remainder was treated as production trajectories.

All electronic structure calculations and the ground-state QM/MM MD simulations were performed with the TeraChem[12] software package interfaced with the OpenMM[18] package.

Absorption spectra calculation and the analysis of the excited states' ordering

In order to calculate the absorption spectra and elucidate the character and ordering of the excited states at the Franck-Condon (FC) region, vertical electronic excitation energies and oscillator strengths (*f*'s) of the chromophore in the protein were computed based on 300 conformations sampled from ground state QM/MM MD simulations. The absorption spectra were then calculated by convolving the computed excitation energies and their corresponding oscillator strengths (*f*'s) from the S_0 state to the S_1-S_3 states using Gaussian functions with standard deviations of 0.2 eV.

Here, the QM/MM partition and their coupling scheme remained the same as the ground state QM/MM MD simulations. However, the QM region was treated with the XMS-CASPT2 method based on the reference electronic wavefunctions calculated by the State-Averaged Complete Active Space Self-Consistent Field (SA-CASSCF) method. The XMS-CASPT2 method incorporates both static and dynamic electron correlations. The four lowest singlet states (S_0, S_1, S_2, and S_3) were included in the XMS-CASPT2 calculation and the state-averaging in the SA-CASSCF calculations. The active space of the CASSCF calculations consisted of six electrons and six orbitals (**Fig. S1**), including the π and π* orbitals of the FAD and nearby W400 residue, which participates in the main $\pi \rightarrow \pi^*$ electronic transitions of the initial ET step. The XMS-CASPT2 calculations employed an imaginary shift of 0.2 a.u. to mitigate the intruder state issue and the IPEA shift was not employed. The 6-31G* basis set was employed in the calculation. The MM region was treated with the same force field as the ground state QM/MM simulations. The method is thus abbreviated as XMS-CASPT2//SA-4-CASSCF(6,6)/6-31G*/MM.[19] Our choice of a medium-sized active space was designed to maintain consistency with the nonadiabatic dynamics and

excited-state dynamics simulations, which were performed with the SA-4-CASSCF(6,6)/6-31G*/MM method (see below).

To analyze the ordering of the singlet adiabatic excited states (S_1-S_3) in the FC region, the character of each state of every conformation used in the spectra calculation was assigned by Mulliken charge analysis and further confirmed by dipole moments of the state and its oscillator strength from the ground state ($S_0 \rightarrow S_n$, n=1, 2, 3). Each state was categorized as having either a dominant local excitation (LE) character with intramolecular $\pi \rightarrow \pi^*$ electronic transitions on the FAD (i.e., prior to the ET event), or a dominant charge-transfer (CT) character with intermolecular $\pi \rightarrow \pi^*$ transition from the W400 residue to FAD (i.e., after the ET event). A state was categorized as having a CT character when the total Mulliken charge on the W400 residue was above +0.7e and the total charge on the FAD molecule was below –0.7e, indicating significant ET from W400 to FAD, which usually has a dipole moment above 25 Debye. Otherwise, it was characterized as having an LE character, which usually has a dipole moment below 20 Debye. Following the character assignment, the order of characters for all excited states was recorded for every conformation. The analysis of excited-state character and ordering were performed at both SA-4-CASSCF(6,6)/6-31G*/MM and the XMS-CASPT2//SA-4-CASSCF(6,6)/6-31G*/MM levels of theory. All QM/MM calculations in this step were performed using the OpenMolcas[20] interfaced with the Tinker software packages.[21]

Systematic benchmark calculations regarding our choices of the active space, electronic structure method and basis set are provided in the **Benchmark calculations** section below.

Nonadiabatic dynamics simulations

The nonadiabatic ET process, which involves the $S_2 \rightarrow S_1$ non-radiative decay, was investigated using the Stochastic-Selection version of the AIMS (SSAIMS) method.[22] The full multiple spawning algorithm (FMS)[23] represents the nuclear wavefunctions as a linear combination of trajectory basis functions (TBFs)

that are frozen-width Gaussian functions, whose centers move classically on the Born-Oppenheimer PESs and whose amplitudes get updated on-the-fly during the dynamics. When a TBF on one state (state A) travels to a region of PES with high nonadiabatic coupling with another state (state B), new TBFs are spawned and propagated on the other state (state B), thus realizing the nonadiabatic population transfer (A→B). In the AIMS simulation, the required energies, gradients, and couplings were updated on the fly using QM calculations. By construction, the AIMS simulations avoid the typical over-coherence issues typically encountered in the Ehrenfest and surface hopping methods and, thus, the necessity for *ad hoc* decoherence corrections. The SSAIMS[22] reduces the cost of the original AIMS method when frequent state-crossings are encountered in the dynamics. For each initial condition (IC, the set of coordinates and velocities of all atoms), multiple runs were performed, and the decoupled TBFs were terminated in a probabilistic manner in each run. The statistical ensemble of all TBFs generated in all runs effectively reproduces a single simulation using the original AIMS algorithm starting from the same IC. The SSAIMS can thus avoid the computationally costly scenario encountered by the original AIMS algorithm, where many TBFs are spawned and simultaneously propagated during the nonadiabatic dynamics simulation.

To model a physically meaningful nonadiabatic ET step induced by photoexcitation, the SSAIMS simulations were initiated on the S_2 state from selected ICs sampled from the ground-state QM/MM MD simulations. The ICs were selected based on the following criteria: (1) the S_2 state was a bright LE state (2) the S_1 state was a CT state, and (3) the CASSCF and XMS-CASPT2 QM/MM calculations predicted the same state ordering and the CASSCF QM/MM calculations produce consistent results between the TeraChem/OpenMM and OpenMolcas/Tinker software packages, which used slightly different schemes to treat QM/MM electrostatic embedding. Criterion (3) is critical for ensuring a consistent link between spectra calculation and nonadiabatic dynamics performed with different software packages.

In the SSAIMS simulation, the PESs were evaluated on-the-fly using the SA(4)-CASSCF(6,6)/6-31G*/MM method by TeraChem/OpenMM. A total of 15 initial conditions (IC) were selected. For each IC, five independent SSAIMS runs were performed, each using a unique random number generator seed. This resulted in 75 SSAIMS simulations, each extending to a minimum of 200 fs and a maximum of 500 fs. The S_2 state population was depleted at the end of each simulation. A timestep of 20 a.u. was used for the non-coupling region, and 5 a.u. timestep was used in the coupling region. When two groups of TBFs were completely decoupled, one group was stochastically selected to survive and continue its further propagation, while the other group was eliminated. The survival probability was proportional to the total amplitude of all TBFs in each group. The two groups were considered to be completely decoupled when the overlap between every pair of TBFs, each selected from one group, decreased below a threshold value of 2×10^{-5}. The relatively short simulation time was used since we mainly focused on the possibility of $S_2 \rightarrow S_1$ nonadiabatic relaxation associated with the first ET step, and we did not consider the $S_1 \rightarrow S_0$ relaxation, which could have been observed in our simulation had we used a much longer time scale. The threshold of total energy conservation was set to 0.006 a.u. The saddle point approximation was employed to calculate the Hamiltonian matrix elements. All SSAIMS simulations were propagated using the FMS90 code interfaced with the TeraChem/OpenMM packages.

The time evolution of excited-state populations, as well as the molecular charges on the FAD and the W400 residue, were analyzed by averaging over all TBFs in all independent SSAIMS runs of all ICs, correctly taking into account the effects of stochastically terminating decoupled TBFs.[22]

Excited-state QM/MM adiabatic dynamics simulation

After the SSAIMS simulations, all S_2 state TBFs' populations have been depleted (Results), and the surviving TBFs on the S_1 state in each run of all ICs were adiabatically propagated on the S_1 state for 1 ps in the constant NVE ensemble, restarting from the coordinates, velocities and the previously optimized

molecular orbitals that comprise the active space of the last step of SSAIMS simulation. The adiabatic dynamics were integrated using a time step of 0.5 fs, employing the same QM/MM settings as the SSAIMS simulations (i.e., SA(4)-CASSCF(6,6)/6-31G*/MM method). The S_1 state's character (LE or CT), dipole moment (μ), and the S_0-S_1 energy gap were monitored during the adiabatic dynamics.

To simulate adiabatic ET induced by direct photoexcitation to the S_1 state, the S_1-state adiabatic QM/MM dynamics simulations were also performed starting from 50 ICs at the FC region, whose S_1 state has a bright LE character. The same QM/MM setting and integration scheme was applied.

Optimization of critical points and reaction pathways on the excited states

To characterize the adiabatic ET pathway on the S_1 state, the last snapshots of the post-AIMS adiabatic S_1 state trajectories were obtained, which resided in multiple minima with LE and CT characters on the S_1 state. Subsequently, constrained geometry optimizations were performed in the protein environment in the QM/MM setting, using the same QM/MM settings. All atoms in the system were fully relaxed on the S_1 state, identifying minima with LE and CT characters. Then, the Nudged Elastic Band (NEB) method[24] was employed to optimize the minimum energy pathway (MEP) connecting the LE and CT minima on the S_1 state. In the MEP optimization, an active region was chosen during the constrained optimization, which included the FWD complex. The rest of the system was assigned in the inactive region. All atoms in the active region were allowed to fully relax during the MEP optimization, whereas the atoms in the inactive region were kept frozen. The same inactive region was used for optimizing the two endpoints of the MEP, i.e., the LE and CT minima, as well as all images of MEP. This was to ensure that the energy fluctuation along the MEP mainly arose from conformational changes in the active region instead of random fluctuations of all solvent molecules.

Systematic benchmark calculations regarding our choices of the active space, electronic structure method and basis set are provided in the **Benchmark calculations** section below.

Additionally, constrained and unconstrained optimizations on the S_1 state were conducted in the gas phase for the FAD-W400-D396 (FWD) complex (**Fig. 5**) to analyze the effects of the protein on the ET. The FWD complex was isolated from the protein and capped with H atoms to saturate the covalent bonds in the same way as the QM/MM boundary treatment. Both types of gas-phase optimizations started from the FWD complex structure in the LE minima in the protein, which was identified by S_1-state adiabatic dynamics after non-radiative decay (SSAIMS). In these optimizations, only the FWD complex was allowed to relax. In the constrained optimization, the C atoms previously at the QM/MM boundary, i.e., the C_β atoms of the W400 and D396 residues and the C atom in the methyl group connected to the N5 atom in the isoalloxazine ring on FAD (**Fig. 1C**) were fixed to their positions in the protein, and the remaining atoms were allowed to fully relax. In unconstrained optimizations, all atoms were allowed to relax in the gas phase. The same QM method was employed as described above for the QM/MM approach.

Key figures and tables

Table S1. $S_0 \rightarrow S_1$ vertical excitation energies (ΔE, in eV), oscillator strengths (*f*), and S_1-state dipole moments (μ, in Debye) for the LE and CT S_1 minima in *At*CRY1. The LE minima are classified into low-energy (ΔE < 2.8 eV) and higher-energy (ΔE > 3 eV) minima. The geometry optimizations and calculations were performed at the SA-4-CASSCF(6,6)/6-31G*/MM level of theory. For each type of minima, three structures were sampled and optimized from the S_1-state adiabatic trajectories following the SSAIMS simulation.

Type of minimum	Structure 1			Structure 2			Structure 3		
	ΔE	*f*	μ	ΔE	*f*	μ	ΔE	*f*	μ
Low-energy LE_{min}	2.78	0.152	10.33	2.64	0.123	11.39	2.64	0.126	12.65
High-energy LE_{min}	3.22	0.217	10.87	3.12	0.393	11.72	3.02	0.386	12.81
CT_{min}	2.41	0.000	28.92	2.47	0.001	29.02	2.18	0.001	31.65

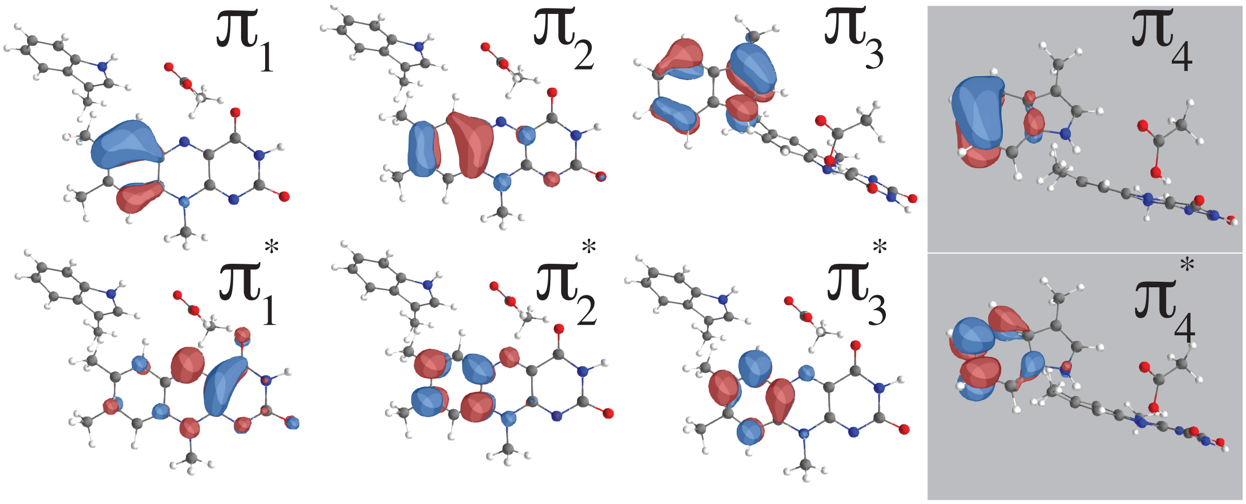

Figure S1. Qualitative representations of molecular orbitals in the active space of the SA-4-CASSCF(6e,6o) and SA-4-CASSCF(8e,8o) calculations. Orbitals with white backgrounds were included in both active spaces, and the additional two orbitals (π_4 and π_4^*) in gray backgrounds were included in the 8e,8o active space. The orbitals were converged from a SA-4-CASSCF(8e,8o)/6-31G*/MM calculation in the protein environment. The molecular structure was obtained from a ground-state QM/MM adiabatic simulation performed at the ωPBEh/6-31G*/MM level of theory. The ground-state (S_0) mainly comprises a closed-shell structure with about 90% weight. In this molecular configuration, the first singlet excited state (S_1) has a primary CT character involving a single-electron excitation from the π_3 orbital localized on the W400 residue to the π_1^* orbital on the FAD molecule ($\pi_3 \rightarrow \pi_1^*$).

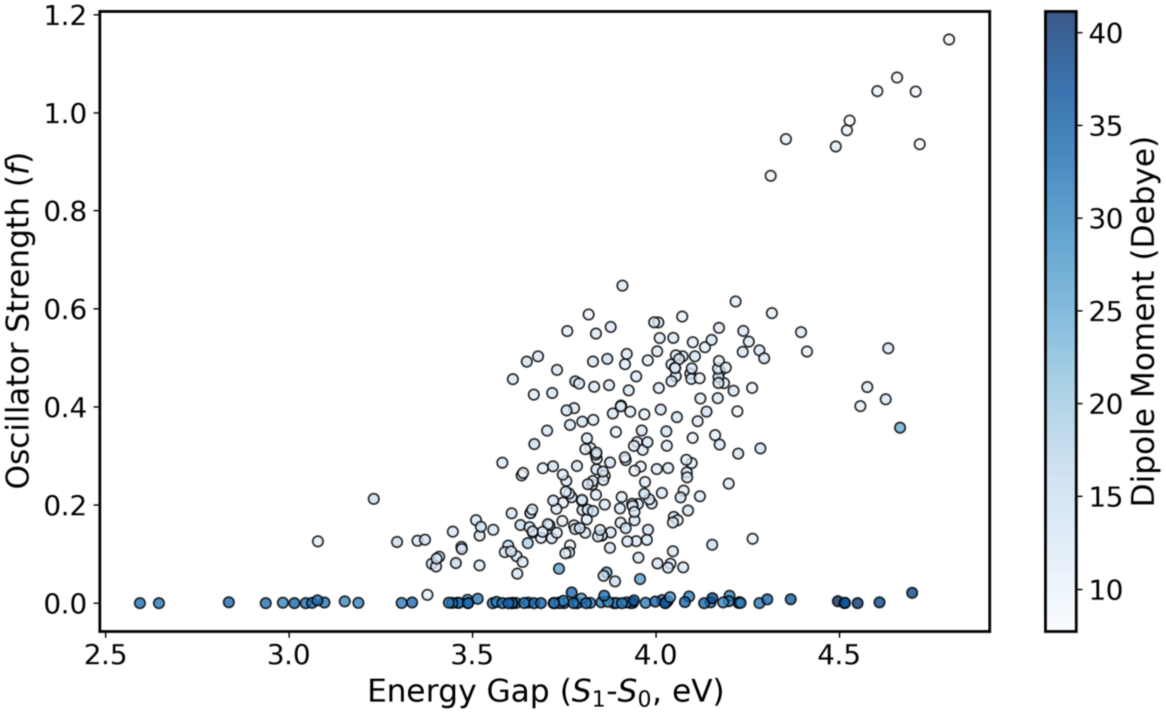

Figure S2. Correlation between the S_0-S_1 energy gap, $S_0 \rightarrow S_1$ oscillator strength (*f*), and S_1-state dipole moment (Debye) for the FWD complex embedded in the *At*CRY1. The S_1 states with LE character on the FAD exhibit high oscillator strength and low dipole moments (0–20 Debye), whereas those with CT character involve intermolecular electron transfer from W400 to the FAD moiety and exhibit near-zero oscillator strength and high dipole moments (> 25 Debye). The analysis was carried out for 300 ICs sampled from the ground-state QM/MM MD equilibration. All calculations were performed at SA-4-CASSCF(6,6)/6-31G*/MM level of theory.

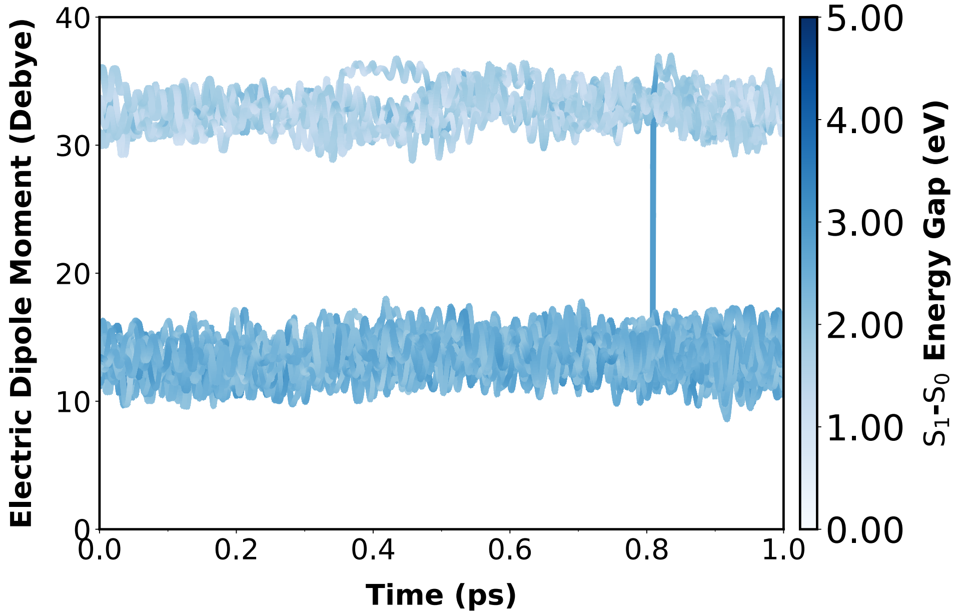

Figure S3. Time evolution of the S_1 dipole moment of the S_1 adiabatic trajectories restarting from the SSAIMS S_1-state TBFs. The shade of the color represents the S_1-S_0 energy gap. Only one LE→CT character transition (at ~0.8 ps) was observed out of 75 trajectories. The trajectories are mostly stabilized in the S_1-state LE and CT minima visited soon after the S_2→S_1 decay. The S_1-S_0 energy gaps oscillate between 0.70 eV and 3.67 eV throughout the simulation. All on-the-fly electronic structure calculations were carried out at SA-4-CASSCF(6,6)/6-31G*/MM level of theory.

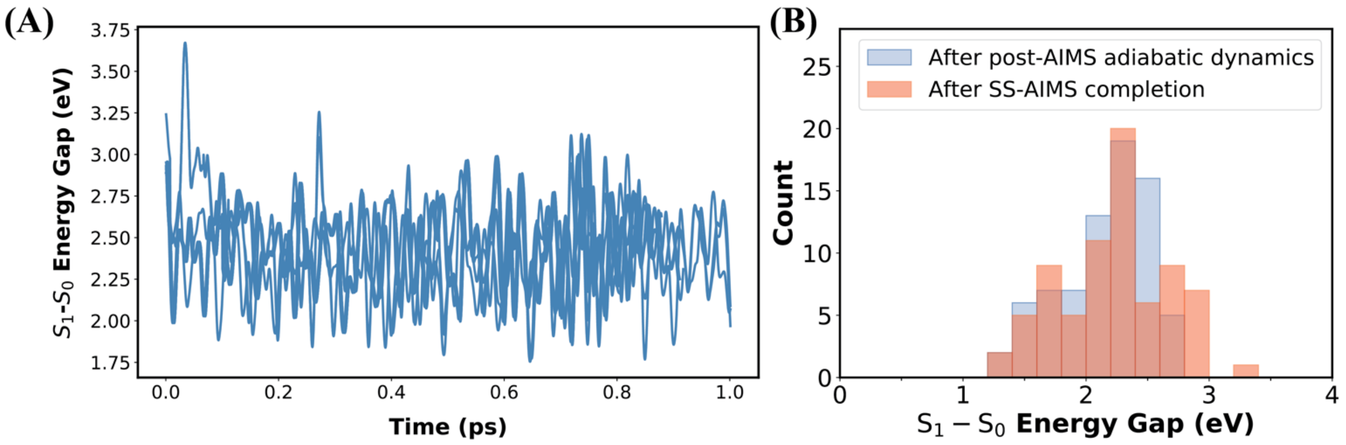

Figure S4. (A) Evolution of the S_1-S_0 energy gap along the 1 ps S_1-state adiabatic dynamics restarting from eight different S_1-state TBFs in the SSAIMS simulations. They all started from S_0-S_1 energy gaps of ~ 3 eV, representing the high-energy LE minima. Within one picosecond simulation time, all trajectories have evolved into and got stabilized in the low-energy LE minima with a lower S_0-S_1 energy gap below 2.8 eV. (B) Distribution of the S_0-S_1 energy gap (in eV) of conformations after the SSAIMS simulation was completed (red) and after 1 ps adiabatic dynamics on the S_1 state following the SSAIMS simulation (blue). Each histogram comprises 75 TBFs/trajectories. The disappearance of frequency near and close to and above 3 eV in red compared to blue histograms corresponds to the evolution of S_1-state dynamics from the high-energy to low-energy LE minima.

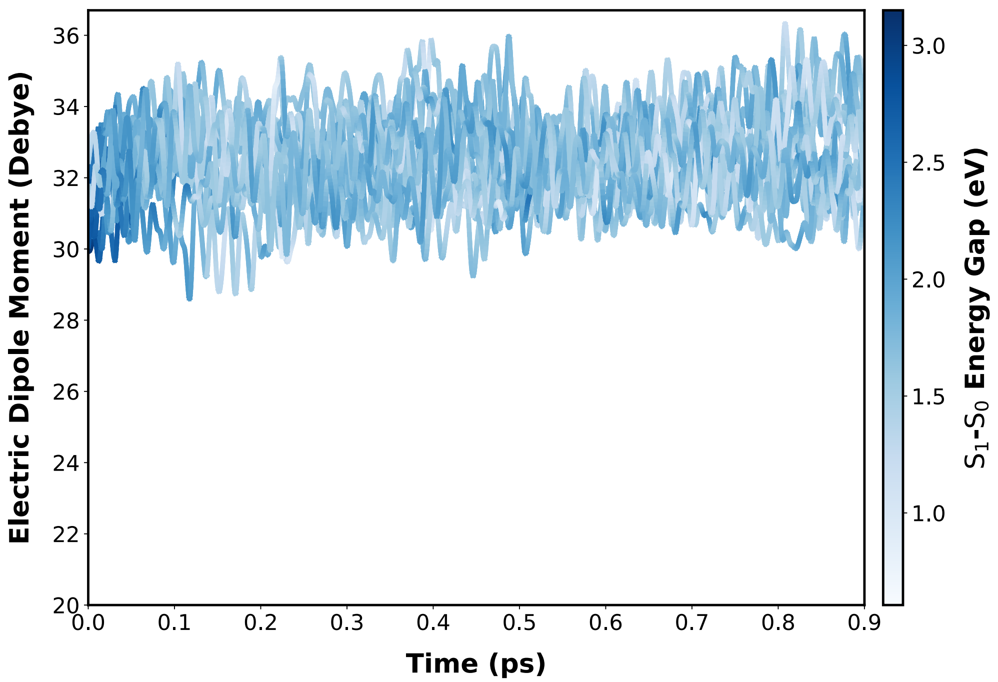

Figure S5. Time evolution of the S_1 dipole moment of 15 S_1 adiabatic trajectories starting from the optimized S_1-state CT minima and random velocities and propagated in the constant NVT ensemble. The shade of the color represents the S_1-S_0 energy gap. All trajectories are stabilized in the S_1-state CT minima visited after the $S_2 \rightarrow S_1$ decay. The S_1-S_0 energy gaps oscillate between 0.61 eV and 3.15 eV throughout the simulation. All on-the-fly electronic structure calculations were carried out at SA-4-CASSCF(6,6)/6-31G*/MM level of theory.

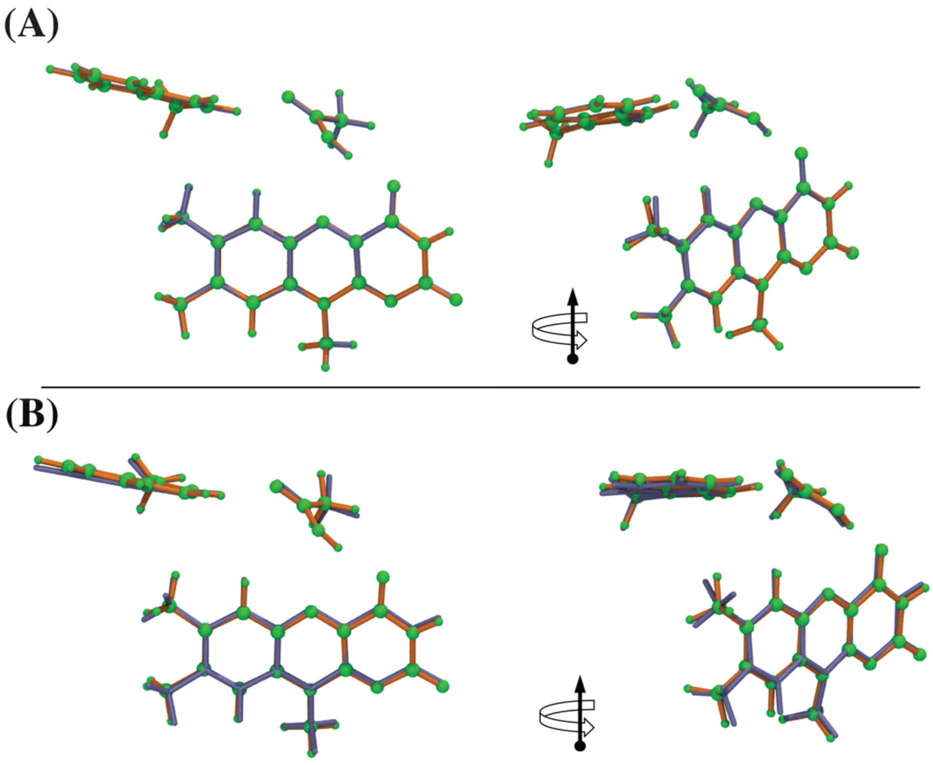

Figure S6. Structural comparison of the critical points along the S_1-state MEP (optimized with the SA-4-CASSCF(6,6)/6-31G*/MM method). (A) Structural transition from the low-energy LE minimum (LE_{low}) to the high-energy LE minimum (LE_{high}). (B) Structural transition from the high-energy LE minimum (LE_{high}) to the CT minima. In all structure comparisons, orange sticks represent the starting minimum, blue sticks represent the ending minimum, and green balls represent the image at the barrier top of MEP. Arrows indicate the viewing angle reorientation in each panel. The similarity in these geometries indicates minimal geometric displacement for the initial ET step in *At*CRY1.

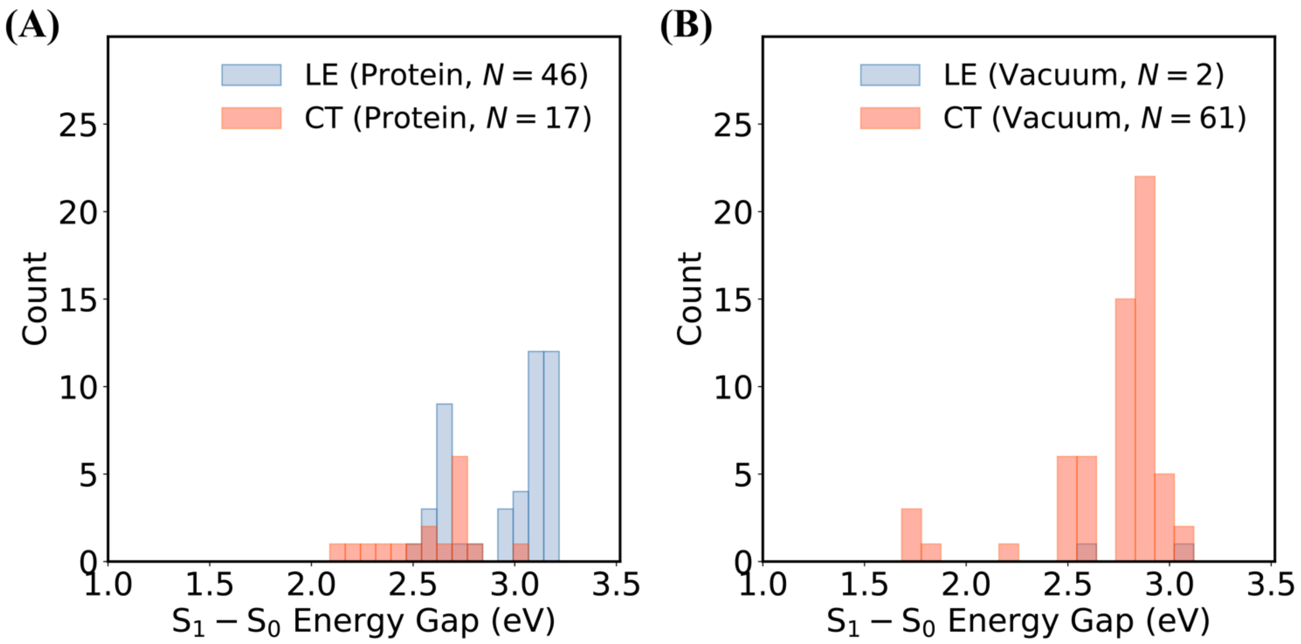

Figure S7. The distributions of the S_1-S_0 energy gap illustrating the characters of the S_1-states within (A) and without (B) the electrostatic environment of the protein. The single-point calculations were performed on the same 63 geometries of the FWD obtained by S_1-state QM/MM optimization in the protein, which were originally sampled from S_1-state adiabatic dynamics after SSAIMS simulation. The LE and CT characters were analyzed using Mulliken charges of the FAD and W400 residues and are colored in blue and red, respectively. The QM region was treated at the SA-4-CASSCF(6,6)/6-31G* level of theory in both the protein and the vacuum.

Benchmark calculations

Ground-state conformations in the Franck-Condon region

In our investigation of the photoinduced ET in *At*CRY1 using SA-CASSCF, we employed a 6-electron, 6-orbital (i.e., (6e,6o)) active space, with state averaging over the four lowest singlet states. This active space was selected to capture the key electronic processes governing the reaction mechanism, specifically the intramolecular π→π* local excitation (LE) within the FAD moiety and the intermolecular π→π* charge-transfer (CT) excitation from the nearest tryptophan residue (W400) to FAD. This active space was chosen in consideration of cost-accuracy trade-off for nonadiabatic and adiabatic dynamics simulations. The chosen active space effectively encompasses the essential orbitals involved in the formation of the radical pair [$FAD^{\bullet-}$ + $W400^{\bullet+}$]. Also, it is larger than the (4e, 3o) active space in the SA-CASSCF optimizations performed by Solov'yov et. al[4] (*J. Am. Chem. Soc.* **2012**, *134* (43), 18046-18052.) when describing the same photoinduced ET step in *At*CRY1.

To further benchmark the robustness of the SA-4-CASSCF(6,6) active space, we extracted 5 snapshots of the FWD complex from the protein environment sampled in the FC region by ground-state QM/MM MD simulations, and performed single-point energy calculations in the vacuum, and calculated its single-point energies of ground and excited states using different wavefunction-based methods, as summarized in **Table S2**. The benchmark methods include extended multi-state CASPT2 (XMS-CASPT2) with (6e,6o) and (8e,8o) active spaces, algebraic diagrammatic construction to second order (ADC(2)), and approximate coupled-cluster singles and doubles (CC2). All calculations were carried out in the vacuum using the 6-31G* basis set. The ADC(2) and CC2 calculations were performed using the TURBOMOLE

package (version 7.7). The SA-CASSCF and XMS-CASPT2 calculations were carried out with the OpenMolcas package.

The results in **Table S2** show that methods incorporating dynamic electron correlation exhibit trends consistent with SA-4-CASSCF(6,6). As expected, the excitation energies obtained with SA-CASSCF are blue-shifted relative to those computed with XMS-CASPT2, CC2 and ADC(2), which includes dynamic correlation. However, the nature of the S_1 and S_2 states (i.e., CT or LE) as well as their ordering predicted by SA-4-CASSCF(6,6) are consistent with most of the benchmark methods, validating one of our key conclusions that in the Franck-Condon region, the S_1 and S_2 states can adopt CT and LE characters, respectively.

Table S2. Benchmarking the SA-CASSCF method against higher-level wavefunction methods (XMS-CASPT2, ADC(2) and CC2) incorporating dynamic electron correlations. The excitation energies (ΔE, in eV), oscillator strengths (f), and the nature of the excited states are calculated for the FWD complex in the vacuum. The structures were sampled by ground-state QM/MM MD equilibration simulations with DFT(ωPBEh) as QM method. Two active spaces were tested for both SA-CASSCF and XMS-CASPT2, i.e., (6e,6o) and (8e,8o), with state averaging over four electronic states. Results obtained with the (8e,8o) active space are reported in **bold** font below the results of (6e,6o) in normal font. All calculations employed the 6-31G* basis set. The excitation energy (ΔE, in eV) and osccilator strengths (*f*), and the the nature of the excited states (LE or CT) are compared. The nature of the adiabatic excited states were assigned based on inspection of the molecular orbitals involved in electronic transition.

	**SA-CASSCF**			**XMS-CASPT2**			**ADC(2)**			**CC2**		
	ΔE	*f*	Nat.	ΔE	*f*	Nat.	ΔE	*f*	Nat.	ΔE	*f*	Nat.
**Geom 1**												
S_1	3.60	0.00	CT	3.07	0.00	CT	2.92	0.00	CT	3.03	0.00	CT
	**3.07**	**0.00**	**CT**	**3.41**	**0.00**	**CT**						
S_2	4.12	0.40	LE	3.46	0.58	LE	3.07	0.28	LE	3.20	0.27	LE
	**4.13**	**0.51**	**LE**	**3.48**	**0.39**	**LE**						
S_3	4.89	0.65	LE	4.35	0.17	LE	3.49	0.00	LE	3.55	0.00	LE
	**4.56**	**0.00**	**LE**	**4.55**	**0.00**	**LE**						
**Geom 2**												
S_1	3.55	0.00	CT	2.90	0.00	CT	2.75	0.00	CT	2.89	0.00	CT
	**3.51**	**0.00**	**CT**	**3.00**	**0.00**	**LE**						
S_2	4.05	0.26	LE	3.32	0.56	LE	2.81	0.24	LE	2.98	0.24	LE
	**4.05**	**0.25**	**LE**	**3.34**	**0.56**	**LE**						
S_3	4.73	0.76	LE	4.18	0.15	LE	3.41	0.01	LE	3.57	0.00	LE
	**4.73**	**0.76**	**LE**	**4.19**	**0.15**	**LE**						

**Geom 3**												
S_1	3.54	0.00	CT	3.01	0.00	CT	2.76	0.18	CT	2.90	0.18	CT
	**3.52**	**0.00**	**CT**	**3.08**	**0.00**	**CT**						
S_2	3.63	0.20	LE	3.27	0.35	LE	2.84	0.00	LE	2.98	0.00	LE
	**3.63**	**0.20**	**LE**	**3.28**	**0.35**	**LE**						
S_3	4.71	0.70	LE	4.27	0.29	LE	3.49	0.00	LE	3.54	0.00	LE
	**4.71**	**0.70**	**LE**	**4.28**	**0.30**	**LE**						
**Geom 4**												
S_1	3.74	0.00	CT	3.26	0.00	CT	2.97	0.27	LE	3.13	0.27	LE
	**3.68**	**0.00**	**CT**	**3.40**	**0.44**	**CT**						
S_2	3.96	0.27	LE	3.38	0.46	LE	3.09	0.00	CT	3.22	0.00	CT
	**3.96**	**0.26**	**LE**	**3.41**	**0.02**	**LE**						
S_3	4.90	0.73	LE	4.34	0.21	LE	3.57	0.00	LE	3.61	0.00	LE
	**4.84**	**0.66**	**LE**	**4.36**	**0.22**	**LE**						
**Geom 5**												
S_1	3.58	0.28	CT	2.84	0.44	CT	2.63	0.20	CT	2.78	0.20	CT
	**3.97**	**0.42**	**CT**	**2.96**	**0.30**	**CT**						
S_2	4.38	0.00	LE	3.18	0.00	LE	2.92	0.00	LE	3.05	0.00	LE
	**4.06**	**0.00**	**LE**	**3.27**	**0.00**	**LE**						
S_3	4.88	0.65	LE	4.09	0.19	LE	3.30	0.00	CT	3.39	0.00	CT
	**4.53**	**0.01**	**LE**	**3.42**	**0.01**	**LE**						

We performed additional QM/MM benchmark calculations comparing the excitation energy, oscillator strength and nature of the excited states for a new set of geometries in the FC region calculated at the XMS-CASPT2 and SA-CASSCF levels of theory, with both (6e,6o) and (8e, 8o) active spaces. The results are summarized in **Table S3**. Different from the data in **Table S2**, these calculations were performed in the QM/MM settings, and the QM region included the FWD complex.

As expected, including dynamic electron correlation (XMS-CASPT2) results in a redshift in the excitation energies compared to SA-CASSCF. However, it is important to note that the ordering of the excited states predicted by SA-CASSCF remains consistent upon XMS-CASPT2 energy correction. This state ordering is particularly robust in two representative cases: (1) the S_1 and S_2 state adopts LE and CT characters, respectively, and (2) the S_1 and S_2 state adopts CT and LE characters, respectively. These QM/MM

benchmarks further validate the reliability of the SA-CASSCF wavefunction for treating photoinduced ET in *At*CRY1.

Table S3. Benchmarking the SA-CASSCF/MM method against the XMS-CASPT2/MM method. The geometries were sampled from the ground-state QM/MM MD equilibration in the FC region. The excitation energy (ΔE, in eV) and oscillator strengths (f), and the nature of the excited states (LE or CT) are compared.

**Geometry/ State**	**SA-CASSCF(6,6)**			**XMS-CASPT2(6,6)**			**SA-CASSCF(8,8)**			**XMS-CASPT2(8,8)**		
	ΔE	*f*	Nat.	ΔE	*f*	Nat.	ΔE	*f*	Nat.	ΔE	*f*	Nat.
**Geom 1**												
S_1	2.97	0.00	LE	2.82	0.00	LE	3.22	0.00	LE	2.89	0.00	LE
S_2	4.66	1.13	CT	3.24	0.75	CT	4.26	0.94	CT	3.24	0.71	CT
S_3	5.76	0.00	LE	5.00	0.00	LE	5.85	0.00	LE	4.83	0.00	LE
**Geom 2**												
S_1	4.60	0.63	LE	3.20	0.48	LE	4.40	0.82	LE	3.15	0.46	LE
S_2	5.12	0.02	CT	3.49	0.00	CT	5.18	0.00	CT	3.57	0.00	CT
S_3	6.17	0.55	LE	4.36	0.23	LE	6.28	0.06	LE	4.39	0.16	LE
**Geom 3**												
S_1	3.74	0.00	CT	2.97	0.00	CT	3.66	0.00	CT	2.95	0.00	CT
S_2	3.88	0.31	LE	3.17	0.49	LE	3.88	0.31	LE	3.19	0.49	LE
S_3	4.79	0.67	LE	4.22	0.21	LE	4.79	0.68	LE	4.23	0.22	LE
**Geom 4**												
S_1	3.75	0.15	LE	3.35	0.19	LE	3.75	0.15	LE	3.36	0.19	LE
S_2	3.94	0.00	CT	3.49	0.00	CT	3.89	0.00	CT	3.55	0.00	CT
S_3	4.80	0.53	LE	4.56	0.32	LE	4.80	0.53	LE	4.57	0.32	LE
**Geom 5**												
S_1	3.73	0.18	LE	3.21	0.44	LE	3.73	0.18	LE	3.21	0.43	LE
S_2	4.06	0.00	CT	3.47	0.00	CT	3.97	0.00	CT	3.55	0.00	CT
S_3	4.74	0.78	LE	4.05	0.24	LE	4.74	0.78	LE	4.07	0.24	LE

To further validate our choices of the (6e,6o) and (8e,8o) active space, we evaluated the occupation numbers of both occupied and virtual natural orbitals after the SA-CASSCF/MM calculations for five distinct geometries in the FC region (same as those used in **Table S3**), as summarized in **Table S4**. In both SA-4-CASSCF(6e,6o) and SA-4-CASSCF(8e,8o) calculations, the occupancy numbers of natural orbitals of the S_1 and S_2 states quickly decay to near zero for orbitals above LOMO ($\boldsymbol{\pi}_1^*$), and quickly increase to near two below HOMO ($\boldsymbol{\pi}_4$). This analysis suggests that the dominant electronic excitations in the S_1 and S_2 states are localized near these two orbitals, and both active spaces are large enough to describe the

multireference electronic wavefunctions associated with them. Expanding the (6e, 6o) into the (8e, 8o) active spaces adds one π and one π^* orbital localized on the W400 residue (Figure S1).

Table S4. The occupation numbers of natural orbitals of S_1 and S_2 states obtained from SA-CASSCF/MM calculations with (6e, 6o) and (8e, 8o) active spaces. The analysis was performed at ground-state geometries equilibrated in the FC region within the protein environment.

**Geometry/State**	π_1	π_2	π_3	π_4	π_1^*	π_2^*	π_3^*	π_4^*
**Geom 1**								
S_1 (8e,8o)	1.97	1.95	1.95	1.01	1.00	0.05	0.04	0.03
S_2 (8e,8o)	2.00	1.94	1.92	1.01	0.98	0.06	0.05	0.04
S_1 (6e,6o)		1.95	1.95	1.01	0.99	0.05	0.04	
S_2 (6e,6o)		2.00	1.94	1.34	0.65	0.06	0.01	
**Geom2**								
S_1 (8e,8o)	1.98	1.94	1.90	1.26	0.73	0.08	0.06	0.06
S_2 (8e,8o)	1.95	1.93	1.92	1.12	0.88	0.08	0.07	0.06
S_1 (6e,6o)		2.00	1.88	1.30	0.71	0.08	0.04	
S_2 (6e,6o)		1.94	1.92	1.00	1.00	0.08	0.06	
**Geom 3**								
S_1 (8e,8o)	1.94	1.94	1.94	1.02	0.98	0.06	0.06	0.05
S_2 (8e,8o)	2.00	1.96	1.89	1.34	0.67	0.06	0.04	0.03
S_1 (6e,6o)		1.94	1.94	1.03	0.97	0.06	0.06	
S_2 (6e,6o)		2.00	1.89	1.34	0.67	0.06	0.03	
**Geom 4**								
S_1 (8e,8o)	2.00	1.95	1.90	1.24	0.75	0.08	0.05	0.04
S_2 (8e,8o)	1.94	1.94	1.93	1.00	1.00	0.07	0.06	0.06
S_1 (6e,6o)		2.00	1.90	1.24	0.75	0.08	0.04	
S_2 (6e,6o)		1.94	1.93	1.00	1.00	0.07	0.06	
**Geom 5**								
S_1 (8e,8o)	2.00	1.94	1.90	1.22	0.78	0.07	0.06	0.03
S_2 (8e,8o)	1.94	1.93	1.92	1.02	0.99	0.07	0.07	0.06
S_1 (6e,6o)		2.00	1.90	1.22	0.78	0.07	0.03	
S_2 (6e,6o)		1.94	1.93	1.01	0.99	0.07	0.06	

Excited-state conformations from Franck-Condon regions

We extended our benchmark analysis by XMS-CASPT2 single-point energy calculations at geometries located beyond the FC region. Single-point energy calculations were carried out at XMS-CASPT2/SA-4-CASSCF(8,8)/6-31G*/MM level of theory along the S_1-state minimum energy pathways (MEPs) from the low-energy LE minimum (LE_{low}) to the high-energy LE minimum (LE_{high}), as well as from LE_{high} to the CT minimum. Note that the three minima and two MEPs were optimized on the S_1 state at the SA-CASSCF(6,6)/6-31G*/MM level of theory. **Figures S8** and **S9** display the XMS-CASPT2/MM PES along the $LE_{low} \rightarrow LE_{high}$ and, $LE_{high} \rightarrow$ CT minimum pathways, respectively.

After the XMS-CASPT2 energy corrections, we observe S_1-state PESs similar to those predicted by the SA-CASSCF method. The MEP from LE_{low} to LE_{high} is endergonic by ~3.5 kcal/mol (**Figure S8**), compared to ~5 kcal/mol predicted at the SA-CASSCF(6,6) level of theory (**Figure 4A**). The MEP from LE_{high} to CT minimum is exergonic by ~7 kcal/mol as predicted by XMS-CASPT2 (**Figure S9**), compared to an exergonicity of ~4.8 kcal/mol predicted by SA-CASSCF. Both methods predict a nearly barrierless pathway from LE_{high} to CT minimum. It is noteworthy that because the $LE_{high} \rightarrow$ CT minimum pathway changes state character through the transition state, and different levels of theories predict slightly different structures of this transition state, the position along the NEB path where the wavefunction switches from the LE to the CT character differ between the two methods (**Figure 4B vs. Figure S9**). This discrepancy, however, does not affect the main conclusion that the $LE_{high} \rightarrow$ CT pathway is mostly barrierless and exergonic.

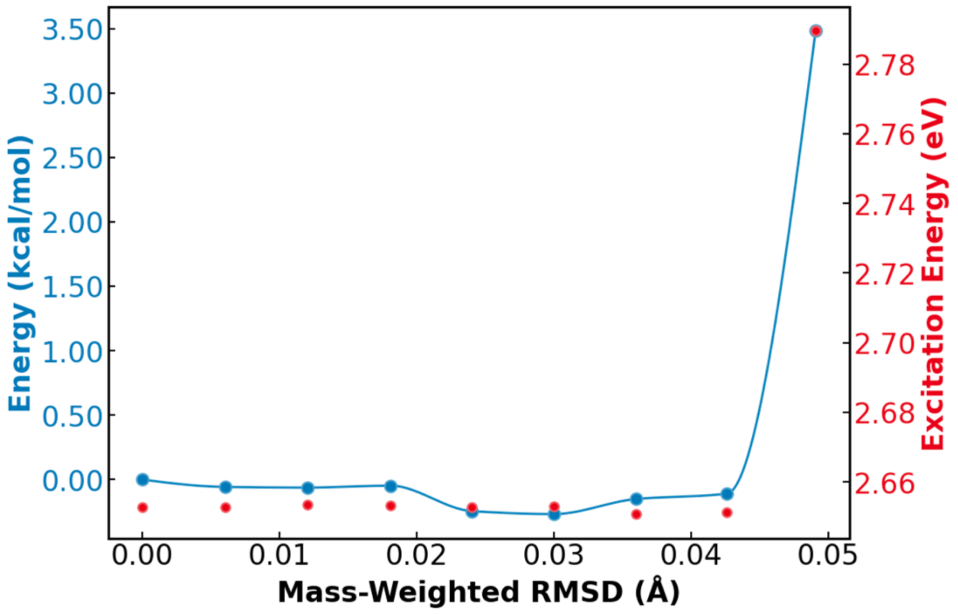

Figure S8. PES along the MEP on the S_1 surface from the low-energy LE minimum (left) and high-energy LE minimum (right). Blue curves and dots represent the energies, while red dots indicate the S_1–S_0 energy gaps. The MEP was optimized at the SA-4-CASSCF(6,6)/6-31G*/MM level, and energies were recalculated on the images along this MEP at the XMS-CASPT2/SA-4-CASSCF(8,8)/6-31G*/MM level of theory.

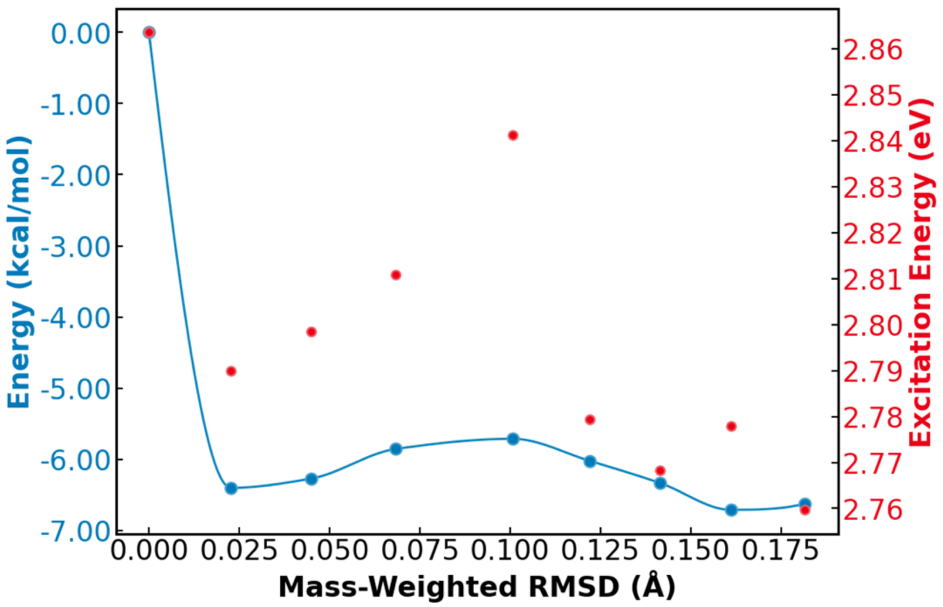

Figure S9. PES along the MEP on the S_1 surface from the high-energy LE minima (left) to the CT minima (right). Blue curves and dots represent the energies, while red dots indicate the S_1–S_0 energy gaps. The MEP was optimized at the SA-4-CASSCF(6,6)/6-31G*/MM level, and energies were recalculated on the images along this MEP at the XMS-CASPT2/SA-4-CASSCF(8,8)/6-31G*/MM level of theory.

In addition, single-point energy calculations were carried out at XMS-CASPT2/SA-4-CASSCF(8,8)/6-31G*/MM level of theory at the minimum energy conical intersections (MECI) between the S_2 and S_1 states (S_2/S_1 MECI), which were optimized at the SA-4-CASSCF(6,6)/6-31G*/MM level of theory (**Table S5**). The S_2 energy of the optimized FC points is higher than the energy at the S_2/S_1 MECI, confirming the conclusions from the SA-CASSCF calculations.

Table S5. XMS-CASPT2/MM energies at the S_2/S_1 MECIs and their relative energies with respect to the FC geometries. The MECIs were optimized at SA-4-CASSCF(6,6)/6-31G*/MM level of theory, and the XMS-CASPT2/MM single-point energies were calculated on top of the MECI structures. The XMS-CASPT2 calculations were performed using SA-4-CASSCF(6,6) and SA-4-CASSCF(8,8) reference wavefunctions and 6-31G* basis set. The results obtained from the (8,8) active space are highlighted in bold font, and (6,6) active space in normal font. For each geometry, the energy difference between the Franck-Condon point and the S_2/S_1 MECI (**ΔE(FC→MECI)**) was computed using the S_2 energy at the FC point and the average energy between S_1 and S_2 states at the MECI.

**Geometry/State**	**Franck-Condon**				**S_2/S_1 MECI**			**ΔE(FC→MECI) (eV)**
	**E (a.u.)**	**ΔE (eV)**	***f***	**Nat.**	**E (a.u.)**	**ΔE (eV)**	**Nat.**	
**Geom 1**								
S_0	-1660.58857				-1660.56986			
	**-1660.58683**				**-1660.57085**			
S_1	-1660.47494	3.09	0.00	CT	-1660.48508	2.31	CT	
	**-1660.46312**	**3.37**	**0.01**	**CT**	**-1660.48401**	**2.36**	**CT**	0.20
S_2	-1660.46986	3.23	0.50	LE	-1660.46931	2.74	LE	**0.39**
	**-1660.46270**	**3.38**	**0.17**	**LE**	**-1660.47003**	**2.74**	**LE**	
S_3	-1660.43857	4.08	0.21	LE	-1660.42453	3.95	LE	
	**-1660.44636**	**3.82**	**0.00**	**CT**	**-1660.42484**	**3.97**	**LE**	
**Geom 2**								
S_0	-1658.50964				-1658.49743			
	**-1658.51317**				**-1658.49469**			
S_1	-1658.39089	3.23	0.00	CT	-1658.40263	2.58	CT	
	**-1658.39555**	**3.20**	**0.20**	**LE**	**-1658.40063**	**2.56**	**CT**	0.26
S_2	-1658.38953	3.27	0.49	LE	-1658.39549	2.77	LE	**0.15**
	**-1658.38947**	**3.37**	**0.00**	**CT**	**-1658.38945**	**2.86**	**LE**	
S_3	-1658.35609	4.18	0.23	LE	-1658.34565	4.13	LE	
	**-1658.35540**	**4.29**	**0.33**	**LE**	**-1658.35408**	**3.83**	**LE**	
**Geom 3**								
S_0	-1657.75063				-1657.73393			
	**-1657.74925**				**-1657.73835**			
S_1	-1657.64063	2.99	0.00	CT	-1657.64294	2.48	LE	0.24
	**-1657.62987**	**3.25**	**0.00**	**CT**	**-1657.63328**	**2.86**	**LE**	**0.07**
S_2	-1657.62724	3.36	0.49	LE	-1657.62882	2.86	CT	

	**-1657.62138**	**3.48**	**0.22**	**LE**	**-1657.61498**	**3.36**	**CT**	
S_3	-1657.59347	4.28	0.23	LE	-1657.55276	4.93	CT	
	**-1657.61083**	**3.77**	**0.00**	**CT**	**-1657.58915**	**4.06**	**CT**	
**Geom 4**								
S_0	-1658.50983				-1658.49662			
	**-1658.51336**				**-1658.49390**			
S_1	-1658.39095	3.23	0.00	CT	-1658.40136	2.59	CT	
	**-1658.39577**	**3.20**	**0.20**	**LE**	**-1658.39936**	**2.57**	**CT**	0.22
S_2	-1658.38977	3.27	0.49	LE	-1658.39469	2.77	LE	**0.12**
	**-1658.38953**	**3.37**	**0.00**	**CT**	**-1658.38862**	**2.86**	**LE**	
S_3	-1658.35622	4.18	0.23	LE	-1658.34503	4.12	LE	
	**-1658.35556**	**4.29**	**0.33**	**LE**	**-1658.35343**	**3.82**	**LE**	
**Geom 5**								
S_0	-1658.51209				-1658.49785			
	**-1658.51308**				**-1658.49459**			
S_1	-1658.39477	3.19	0.20	LE	-1658.40031	2.65	CT	0.16
	**-1658.39550**	**3.20**	**0.20**	**LE**	**-1658.40055**	**2.56**	**CT**	**0.15**
S_2	-1658.39182	3.27	0.00	CT	-1658.39476	2.81	LE	
	**-1658.38945**	**3.36**	**0.00**	**CT**	**-1658.38937**	**2.86**	**LE**	
S_3	-1658.35489	4.28	0.33	LE	-1658.36215	3.69	CT	
	**-1658.35535**	**4.29**	**0.33**	**LE**	**-1658.35407**	**3.82**	**LE**	

Consistency between ground-state sampling and nonadiabatic dynamics

Since the FC region was sampled by ground-state DFT/MM MD simulations whereas the photodynamics were propagated by the SA-CASSCF method, it is important to evaluate if SA-CASSCF and the DFT (ωPBEh) methods predict similar molecular structures with comparable electronic properties of the excited states. To this end, we carried out constrained ground-state and S_1-state geometry optimizations of the FWD complex in the vacuum, starting from the experimental crystal structure (PDB ID: 1U3C). Given the inherent flexibility of the system, we optimized the geometries sequentially for each individual molecular component in the following order: (1) the FAD moiety, (2) the W400 and (3) the D396 residues. Subsequently, electronic excitation properties, such as excitation energy (ΔE), oscillator strength (f), dipole moment (μ), and the nature of the state were recalculated at SA-4-CASSCF(6,6) level of theory based on these optimized structures (**Table S6**). The geometric parameters (bonds, angles and dihedrals) of the optimized structures by different methods are also compared in **Table S7.** In our test, using different

methods to optimize the FC geometries on the ground state results in similar excitation energies and same state ordering, with minimal differences in the geometric properties.

Table S6. Electronic properties of the FWD complex in vacuum on the top of ground-state FC geometries optimized using SA-4-CASSCF(6,6) and DFT (ωPBEh). The electronic properties were computed at SA-4-CASSCF(6,6). All electronic structure calculations were performed with a 6-31G* atomic basis set in the vacuum.

State	SA-CASSCF//SA-CASSCF				SA-CASSCF//DFT(ωPBEh)			
	ΔE	f	μ	Nat.	ΔE	f	μ	Nat.
S_1	4.28	0.380	10.43	LE	4.07	0.341	10.79	LE
S_2	4.73	0.001	36.10	CT	4.24	0.002	34.08	CT
S_3	5.20	0.674	9.59	LE	5.01	0.674	9.60	LE

Table S7. Comparison of selected bond lengths, bond angles, and dihedral angles of the FWD complex between ground-state and S_1-state constrained-optimized geometries using the SA-4-CASSCF(6,6) and DFT(ωPBEh)/TD-DFT(ωPBEh) methods with the 6-31G* atomic basis set. All optimizations were performed in the vacuum. Bond lengths are reported in angstroms, while angles and dihedral are in degrees. The symbol Δ represents the absolute difference between the values obtained from the two computational approaches.

Geometric Property	S_0				S_1		
	SA-CASSCF	DFT	Δ (S_0)		SA-CASSCF	DFT	Δ (S_1)
**Bond**				**Bond**			
C-O	1.20	1.22	0.02	C-C	1.46	1.39	0.07
C-O	1.32	1.34	0.02	C-N	1.27	1.32	0.04
C-C	1.35	1.37	0.02	N-C	1.41	1.38	0.03
O-C	1.19	1.21	0.02	C-O	1.20	1.22	0.02
C-O	1.19	1.22	0.02	C-O	1.32	1.34	0.02
**Angle**				**Angle**			
C-N-C	120.08	118.21	1.87	C-C-C	117.07	119.80	2.73
C-C-N	124.51	125.51	1.00	C-C-C	123.54	121.67	1.88
N-C-N	120.11	119.18	0.93	O-C-N	122.31	121.00	1.30
C-C-O	125.83	126.60	0.78	O-C-N	120.70	121.97	1.27
C-C-N	119.24	118.49	0.75	C-C-C	116.04	117.11	1.07
**Dihedral**				**Dihedral**			
C-C-N-C	178.48	179.69	1.83	C-C-C-N	179.02	178.41	2.57
C-C-N-C	1.47	0.24	1.71	C-C-C-C	178.48	179.31	2.21
C-C-N-C	2.18	0.61	1.57	C-C-C-C	0.73	1.30	2.02
C-N-C-C	2.18	0.61	1.57	N-C-N-C	0.52	1.41	1.94
C-N-C-N	178.12	179.54	1.41	N-C-C-N	1.05	0.76	1.81

Effects of Basis set

The 6-31G* basis set was chosen based on prior studies to optimize the balance between computational efficiency and theoretical accuracy, especially for extensive dynamics simulations as performed in the current study. This basis set has been successfully employed in previous studies[4, 25] investigating flavin photochemistry, both in vacuum conditions and in complex biological environments. To benchmark the selection of this basis set, we performed SA-CASSCF and XMS-CASPT2 calculations using a larger basis set, ANO-L-VDZP, on the same geometries. The test result is summarized in **Table S8**. Most of the time, using a large basis set preserves the state ordering of the lowest LE and CT adiabatic states and decreases the excitation energy to the LE state. Importantly, with the larger basis set, at both SA-CASSCF and XMS-CASPT2 levels of theory, some geometries have the S_1 state with CT character and S_2 state with LE character, which can contribute to the nonadiabatic ET pathway.

Table S8. Benchmark of effects of basis set on the electronic properties. SA-CASSCF and XMS-CASPT2 calculations were performed on representative FC geometries sampled by the ground-state QM/MM MD equilibration. The multireference calculations were performed using different basis sets, 6-31G* and ANO-L-VDZP, with the latter being the larger one. The results of larger basis set is highlighted in bold font, and smaller basis set in normal font. The nature of the electronic nature was verified by the orbitals involved in the electronic transitions and dipole moments.

**Geometry/**	**SA-CASSCF(6,6)**			**XMS-CASPT2(6,6)**			**SA-CASSCF(8,8)**			**XMS-CASPT2(8,8)**		
**State**	**ΔE**	***f***	**Nat.**	**ΔE**	***f***	**Nat.**	**ΔE**	***f***	**Nat.**	**ΔE**	***f***	**Nat.**
**Geom 1**												
S_1	2.97	0.00	LE	2.82	0.00	LE	3.22	0.00	LE	2.89	0.00	LE
	**3.68**	**0.00**		**2.71**	**0.00**	**CT**	**3.71**	**0.00**	**CT**	**2.69**	**0.00**	**CT**
S_2	4.66	1.13	CT	3.24	0.75	CT	4.26	0.94	CT	3.24	0.71	CT
	**4.72**	**0.94**		**3.20**	**0.58**	**LE**	**4.19**	**0.30**	**LE**	**3.28**	**0.45**	**LE**
S_3	5.76	0.00	LE	5.00	0.00	LE	5.85	0.00	LE	4.83	0.00	LE
	**5.16**	**0.09**		**3.95**	**0.12**	**LE**	**4.20**	**0.50**	**LE**	**3.63**	**0.16**	**LE**
**Geom 2**												
S_1	4.60	0.63	LE	3.20	0.48	LE	4.40	0.82	LE	3.15	0.46	LE
	**3.84**	**0.35**	**LE**	**3.05**	**0.47**	**LE**	**3.66**	**0.12**	**LE**	**3.21**	**0.32**	**LE**
S_2	5.12	0.02	CT	3.49	0.00	CT	5.18	0.00	CT	3.57	0.00	CT
	**4.16**	**0.00**	**CT**	**3.31**	**0.00**	**CT**	**4.14**	**0.00**	**CT**	**3.30**	**0.00**	**CT**
S_3	6.17	0.55	LE	4.36	0.23	LE	6.28	0.06	LE	4.39	0.16	LE
	**4.86**	**0.65**	**LE**	**4.20**	**0.23**	**LE**	**4.43**	**0.73**	**LE**	**3.91**	**0.30**	**LE**
**Geom 3**												
S_1	3.74	0.00	CT	2.97	0.00	CT	3.66	0.00	CT	2.95	0.00	CT

	**3.72**	**0.01**	**CT**	**2.82**	**0.00**	**CT**	**3.32**	**0.08**	**LE**	**2.81**	**0.00**	**LE**
S_2	3.88	0.31	LE	3.17	0.49	LE	3.88	0.31	LE	3.19	0.49	LE
	**3.79**	**0.28**	**LE**	**3.03**	**0.49**	**LE**	**4.46**	**0.43**	**LE**	**3.12**	**0.42**	**LE**
S_3	4.79	0.67	LE	4.22	0.21	LE	4.79	0.68	LE	4.23	0.22	LE
	**4.71**	**0.72**	**LE**	**4.05**	**0.22**	**LE**	**4.49**	**0.32**	**LE**	**3.63**	**0.24**	**LE**
**Geom 4**												
S_1	3.75	0.15	LE	3.35	0.19	LE	3.75	0.15	LE	3.36	0.19	LE
	**3.63**	**0.14**	**LE**	**3.23**	**0.18**	**LE**	**3.61**	**0.07**	**LE**	**3.29**	**0.00**	**CT**
S_2	3.94	0.00	CT	3.49	0.00	CT	3.89	0.00	CT	3.55	0.00	CT
	**3.89**	**0.00**	**CT**	**3.35**	**0.00**	**CT**	**4.07**	**0.00**	**CT**	**3.46**	**0.17**	**LE**
S_3	4.80	0.53	LE	4.56	0.32	LE	4.80	0.53	LE	4.57	0.32	LE
	**4.67**	**0.56**	**LE**	**4.39**	**0.33**	**LE**	**4.61**	**0.68**	**LE**	**4.07**	**0.37**	**LE**
**Geom 5**												
S_1	3.73	0.18	LE	3.21	0.44	LE	3.73	0.18	LE	3.21	0.43	LE
	**3.21**	**0.11**		**3.00**	**0.15**	**LE**	**3.12**	**0.20**	**LE**	**2.61**	**0.25**	**LE**
S_2	4.06	0.00	CT	3.47	0.00	CT	3.97	0.00	CT	3.55	0.00	CT
	**3.83**	**0.00**		**3.36**	**0.00**	**CT**	**3.88**	**0.01**	**CT**	**3.39**	**0.00**	**CT**
S_3	4.74	0.78	LE	4.05	0.24	LE	4.74	0.78	LE	4.07	0.24	LE
	**4.22**	**0.49**		**4.09**	**0.28**	**LE**	**4.11**	**0.44**	**LE**	**3.87**	**0.26**	**LE**

Characterization of the S_2/S_1 MECI

To characterize the S_2/S_1 MECI, the dipole moment (in Debye) was evaluated over a circular path on the branching plane of the MECI. The path is centered at the MECI between the S_1 and S_2 states. The plane was spanned by the difference in the gradients of the two states **g** and the nonadiabatic coupling vector **h between them**, given by the following equations:

$$\mathbf{g} = \frac{1}{2}\left(\frac{\partial E_2}{\partial \mathbf{R}} - \frac{\partial E_1}{\partial \mathbf{R}}\right) \qquad \text{Eq. S1}$$

$$\mathbf{h} = \left\langle \phi_2 \middle| \frac{\partial H}{\partial \mathbf{R}} \middle| \phi_1 \right\rangle \qquad \text{Eq. S2}$$

The vectors **g** and **h** were then orthonormalized to expand the branching plane. Geometries along this circular path on this plane were then generated according to:

$$\boldsymbol{x}_p = \boldsymbol{x}_o + R \cdot (cos\,\theta \cdot \mathbf{g} + sin\,\theta \cdot \mathbf{h}), \quad \text{Eq. S3}$$

where $\boldsymbol{x}_o$ represents the full Cartesian coordinates of the MECI, R is the displacement radius (0.01 Å), and θ is the polar angle in the branching plane relative to the **g** vector and changes from 0 to 360 degrees. All single-point calculations along the circular paths were performed at the SA-4-CASSCF(6,6)/6-31G*/MM level of theory. The initial guess of the CASSCF wavefunction for each point was obtained from an adjacent geometry with a lower θ value.

Figure S10 and S11 summarizes the results of such MECI characterization. It is evident that as the θ value is scanned from 0 to 360 degrees, the S_1-state dipole moment changes from lower to higher values and then back to lower values (**Figure S10**). This process corresponds to the S_1 wavefunction's character changing from LE to CT, and finally to LE along the circular path on the branching plane. This result confirms that the S_2/S_1 MECI arises from the intersection between two diabatic electronic states, one with LE character and the other with CT character.

The sudden increase/decrease of dipole moment near ~0 and ~180 degrees (**Figure S10**) can be explained by the two diabatic surfaces (with LE and CT characters, respectively) crossing each other along the x axis. In the branching plane, the interface of the two PESs can be better described as a pseudo-one-dimensional curve following the **h** vector (x axis) rather than a single zero-dimensional point at the origin, as normally expected for geometries belonging to a typical CI seam space. In other words, the CI seam space here has pseudo 3N-7 degrees of freedom, although formally its total degrees of freedom is still 3N-8. This phenomenon arises from the fact that geometric displacements of the nonadiabatic coupling vector (**h**) only weakly lift the energy degeneracy at the CI geometry, whereas the perpendicular vector (dominated by the **g** vector) introduces a stronger lift of the degeneracy. The sudden change in the electronic wavefunction's character at 0 and 180 degrees is not due to the instability of the SA-CASSCF

active space. The active space stability is confirmed by the continuity of the S_1 and S_2 adiabatic states' energies along the circular path on the branching plane around the MECI (**Figure S11**). Such a phenomenon is expected for typical ET reactions, considering the well-established success of the Marcus theory. The Marcus theory usually employs the 1D PESs of two intersecting diabatic states projected to one solvent reaction coordinate. The success of this 1D depiction of diabatic PESs implies that in the CI branching plane, the two 2D diabatic PESs mostly intersect to generate a pseudo 1D curve, along which the energy degeneracy is only weakly lifted and the diabatic coupling and adiabatic energy gap between the two states change slowly. Here, the **h** vector plays such a role. The geometry displacement along the perpendicular vector on the branching plane, dominated by the **g** vector, is the major driver for the electron transfer event, which is analogous to the reaction coordinate typically used in the Marcus theory.

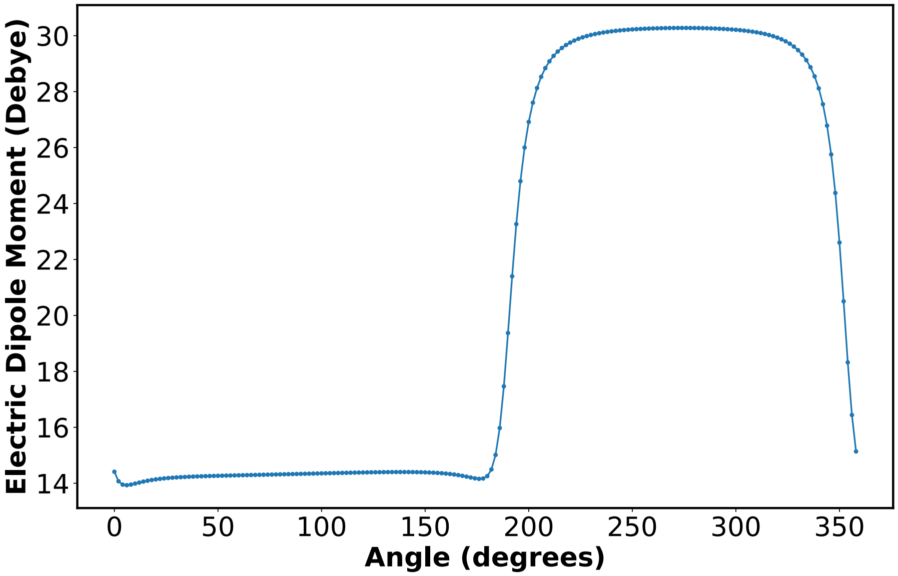

Figure S10. S_1-state dipole moment along the circular path on the branching plane of S_2/S_1 MECI. The branching plane is expanded by the orthonormalized **g** and **h** vectors. The S_2/S_1 MECI is located at the origin of this plane and the center of the circular path around it. The MECI was optimized at the SA-4-CASSCF(6,6)/6-31G*/MM level of theory, and all single-point calculations were performed at the same level of theory. The circular path has a radial distance of 0.01Å from the MECI, and polar angles were sampled in intervals of 2 degrees along this path.

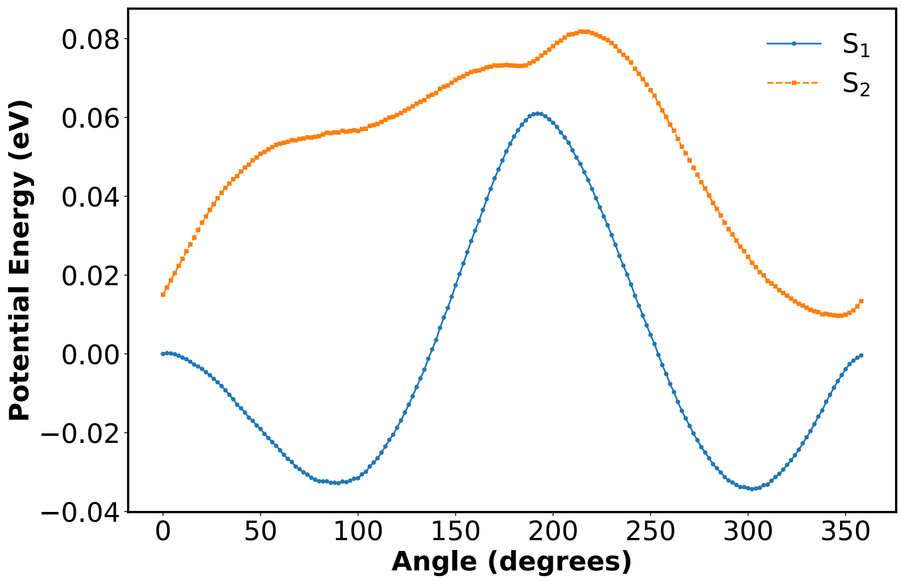

Figure S11. Potential energies of the S_1 and S2 states along the circular path on the branching plane of a S_2/S_1 MECI. The branching plane is expanded by the orthonormalized **g** and **h** vectors. The S_2/S_1 MECI is located at the origin of this plane and the center of the circular path around it. The MECI was optimized at the SA-4-CASSCF(6,6)/6-31G*/MM level of theory, and all single-point calculations were performed at the same level of theory. The circular path has a radial distance of 0.01Å from the MECI, and polar angles were sampled in intervals of 2 degrees along this path.

References

(1) Brautigam, C. A.; Smith, B. S.; Ma, Z.; Palnitkar, M.; Tomchick, D. R.; Machius, M.; Deisenhofer, J. Structure of the photolyase-like domain of cryptochrome 1 from Arabidopsis thaliana. *Proc. Natl. Acad. Sci. U. S. A.* **2004**, *101* (33), 12142-12147. DOI: 10.1073/pnas.0404851101 (acccessed 2025/02/07).

(2) Webb, B.; Sali, A. Comparative Protein Structure Modeling Using MODELLER. *Curr. Protoc. Bioinform.* **2016**, *54* (1), 5.6.1-5.6.37, https://doi.org/10.1002/cpbi.3. DOI: https://doi.org/10.1002/cpbi.3 (acccessed 2021/08/26).

(3) Anandakrishnan, R.; Aguilar, B.; Onufriev, A. V. H++ 3.0: automating p K prediction and the preparation of biomolecular structures for atomistic molecular modeling and simulations. *Nucleic acids research* **2012**, *40* (W1), W537-W541.

(4) Solov'yov, I. A.; Domratcheva, T.; Moughal Shahi, A. R.; Schulten, K. Decrypting Cryptochrome: Revealing the Molecular Identity of the Photoactivation Reaction. *J. Am. Chem. Soc.* **2012**, *134* (43), 18046-18052. DOI: 10.1021/ja3074819.

(5) Cailliez, F.; Müller, P.; Gallois, M.; de la Lande, A. ATP Binding and Aspartate Protonation Enhance Photoinduced Electron Transfer in Plant Cryptochrome. *J. Am. Chem. Soc.* **2014**, *136* (37), 12974-12986. DOI: 10.1021/ja506084f.

(6) Maier, J. A.; Martinez, C.; Kasavajhala, K.; Wickstrom, L.; Hauser, K. E.; Simmerling, C. ff14SB: Improving the Accuracy of Protein Side Chain and Backbone Parameters from ff99SB. *J Chem Theory Comput* **2015**, *11* (8), 3696-3713. DOI: 10.1021/acs.jctc.5b00255 From NLM Medline.

(7) Wu, Y.; Tepper, H. L.; Voth, G. A. Flexible simple point-charge water model with improved liquid-state properties. *J Chem Phys* **2006**, *124* (2), 024503. DOI: 10.1063/1.2136877 From NLM Medline.

(8) Wang, J.; Wolf, R. M.; Caldwell, J. W.; Kollman, P. A.; Case, D. A. Development and testing of a general amber force field. *J. Comput. Chem.* **2004**, *25* (9), 1157-1174. DOI: 10.1002/jcc.20035. Wang, J.; Wang, W.; Kollman, P. A.; Case, D. A. Automatic atom type and bond type perception in molecular mechanical calculations. *J. Mol. Graph. Model.* **2006**, *25* (2), 247-260. DOI: https://doi.org/10.1016/j.jmgm.2005.12.005.
(9) Case, D. A.; Aktulga, H. M.; Belfon, K.; Cerutti, D. S.; Cisneros, G. A.; Cruzeiro, V. W. D.; Forouzesh, N.; Giese, T. J.; Götz, A. W.; Gohlke, H.; et al. AmberTools. *Journal of Chemical Information and Modeling* **2023**, *63* (20), 6183-6191. DOI: 10.1021/acs.jcim.3c01153.
(10) Darden, T.; York, D.; Pedersen, L. Particle mesh Ewald: An N·log(N) method for Ewald sums in large systems. *J. Chem. Phys.* **1993**, *98* (12), 10089-10092. DOI: 10.1063/1.464397 (acccessed 2022/02/13).
(11) Case, D. A.; Belfon, K.; Ben-Shalom, I. Y.; Brozell, S. R.; Cerutti, D. S.; Cheatham, T. E., III; Cruzeiro, V. W. D.; Darden, T. A.; Duke, R. E.; Giambasu, G.; et al. *AMBER 2020*; University of California, San Francisco, 2020.
(12) Ufimtsev, I. S.; Martinez, T. J. Quantum Chemistry on Graphical Processing Units. 3. Analytical Energy Gradients, Geometry Optimization, and First Principles Molecular Dynamics. *J. Chem. Theory Comput.* **2009**, *5* (10), 2619-2628. DOI: 10.1021/ct9003004. Titov, A. V.; Ufimtsev, I. S.; Luehr, N.; Martinez, T. J. Generating Efficient Quantum Chemistry Codes for Novel Architectures. *Journal of Chemical Theory and Computation* **2013**, *9* (1), 213-221. DOI: 10.1021/ct300321a. Seritan, S.; Bannwarth, C.; Fales, B. S.; Hohenstein, E. G.; Kokkila-Schumacher, S. I. L.; Luehr, N.; Snyder, J. W., Jr.; Song, C.; Titov, A. V.; Ufimtsev, I. S.; et al. TeraChem: Accelerating electronic structure and ab initio molecular dynamics with graphical processing units. *The Journal of Chemical Physics* **2020**, *152* (22), 224110. DOI: 10.1063/5.0007615 (acccessed 2/7/2025). Seritan, S.; Bannwarth, C.; Fales, B. S.; Hohenstein, E. G.; Isborn, C. M.; Kokkila-Schumacher, S. I. L.; Li, X.; Liu, F.; Luehr, N.; Snyder Jr, J. W.; et al. TeraChem: A graphical processing unit-accelerated electronic structure package for large-scale ab initio molecular dynamics. *WIREs Computational Molecular Science* **2021**, *11* (2), e1494. DOI: https://doi.org/10.1002/wcms.1494 (acccessed 2025/02/07).
(13) Liang, R.; Yu, J. K.; Meisner, J.; Liu, F.; Martinez, T. J. Electrostatic Control of Photoisomerization in Channelrhodopsin 2. *J. Am. Chem. Soc.* **2021**, *143* (14), 5425-5437. DOI: 10.1021/jacs.1c00058.
(14) Nielsen, C.; Nørby, M. S.; Kongsted, J.; Solov'yov, I. A. Absorption Spectra of FAD Embedded in Cryptochromes. *The Journal of Physical Chemistry Letters* **2018**, *9* (13), 3618-3623. DOI: 10.1021/acs.jpclett.8b01528.
(15) Maseras, F.; Morokuma, K. Imomm - a New Integrated Ab-Initio Plus Molecular Mechanics Geometry Optimization Scheme of Equilibrium Structures and Transition-States. *J. Comput. Chem.* **1995**, *16* (9), 1170-1179. DOI: 10.1002/Jcc.540160911.
(16) Henderson, T. M.; Izmaylov, A. F.; Scalmani, G.; Scuseria, G. E. Can short-range hybrids describe long-range-dependent properties? *The Journal of Chemical Physics* **2009**, *131* (4), 044108. DOI: 10.1063/1.3185673 (acccessed 2/8/2025). Rohrdanz, M. A.; Martins, K. M.; Herbert, J. M. A long-range-corrected density functional that performs well for both ground-state properties and time-dependent density functional theory excitation energies, including charge-transfer excited states. *The Journal of Chemical Physics* **2009**, *130* (5), 054112. DOI: 10.1063/1.3073302 (acccessed 2/8/2025).
(17) Hariharan, P. C.; Pople, J. A. The influence of polarization functions on molecular orbital hydrogenation energies. *Theoretica chimica acta* **1973**, *28* (3), 213-222. DOI: 10.1007/BF00533485. Ditchfield, R.; Hehre, W. J.; Pople, J. A. Self‐Consistent Molecular‐Orbital Methods. IX. An Extended Gaussian‐Type Basis for Molecular‐Orbital Studies of Organic Molecules. *The Journal of Chemical Physics* **2003**, *54* (2), 724-728. DOI: 10.1063/1.1674902 (acccessed 6/28/2023).

(18) Eastman, P.; Friedrichs, M. S.; Chodera, J. D.; Radmer, R. J.; Bruns, C. M.; Ku, J. P.; Beauchamp, K. A.; Lane, T. J.; Wang, L.-P.; Shukla, D.; et al. OpenMM 4: A Reusable, Extensible, Hardware Independent Library for High Performance Molecular Simulation. *J. Chem. Theory Comput.* **2013**, *9* (1), 461-469. DOI: 10.1021/ct300857j.
(19) Kabir, M. P.; Ghosh, P.; Gozem, S. Electronic structure methods for simulating flavin's spectroscopy and photophysics: comparison of multi-reference, TD-DFT, and single-reference wave function methods. *The Journal of Physical Chemistry B* **2024**, *128* (31), 7545-7557.
(20) Fdez. Galván, I.; Vacher, M.; Alavi, A.; Angeli, C.; Aquilante, F.; Autschbach, J.; Bao, J. J.; Bokarev, S. I.; Bogdanov, N. A.; Carlson, R. K.; et al. OpenMolcas: From Source Code to Insight. *Journal of Chemical Theory and Computation* **2019**, *15* (11), 5925-5964. DOI: 10.1021/acs.jctc.9b00532.
(21) Rackers, J. A.; Wang, Z.; Lu, C.; Laury, M. L.; Lagardère, L.; Schnieders, M. J.; Piquemal, J.-P.; Ren, P.; Ponder, J. W. Tinker 8: Software Tools for Molecular Design. *Journal of Chemical Theory and Computation* **2018**, *14* (10), 5273-5289. DOI: 10.1021/acs.jctc.8b00529.
(22) Curchod, B. F. E.; Glover, W. J.; Martínez, T. J. SSAIMS—Stochastic-Selection Ab Initio Multiple Spawning for Efficient Nonadiabatic Molecular Dynamics. *J. Phys. Chem. A* **2020**, *124* (30), 6133-6143. DOI: 10.1021/acs.jpca.0c04113.
(23) Ben-Nun, M.; Quenneville, J.; Martínez, T. J. Ab Initio Multiple Spawning: Photochemistry from First Principles Quantum Molecular Dynamics. *J. Phys. Chem. A* **2000**, *104* (22), 5161-5175. DOI: 10.1021/jp994174i. Ben-Nun, M.; Martínez, T. J. Ab Initio Quantum Molecular Dynamics. In *Advances in Chemical Physics*, John Wiley & Sons, Inc., 2002; pp 439-512. Curchod, B. F. E.; Martínez, T. J. Ab Initio Nonadiabatic Quantum Molecular Dynamics. *Chem. Rev.* **2018**, *118* (7), 3305-3336. DOI: 10.1021/acs.chemrev.7b00423.
(24) Henkelman, G.; Uberuaga, B. P.; Jónsson, H. A climbing image nudged elastic band method for finding saddle points and minimum energy paths. *J. Chem. Phys.* **2000**, *113* (22), 9901-9904. DOI: 10.1063/1.1329672 (acccessed 2020/10/15).
(25) Kar, R. K.; Miller, A.-F.; Mroginski, M.-A. Understanding flavin electronic structure and spectra. *WIREs Computational Molecular Science* **2022**, *12* (2), e1541. DOI: https://doi.org/10.1002/wcms.1541 (acccessed 2025/04/14). Frederiksen, A.; Gerhards, L.; Reinholdt, P.; Kongsted, J.; Solov'yov, I. A. Importance of Polarizable Embedding for Absorption Spectrum Calculations of Arabidopsis thaliana Cryptochrome 1. *J. Phys. Chem. B* **2024**, *128* (26), 6283-6290. DOI: 10.1021/acs.jpcb.4c02168.
