# Supplementary material for: Decrypting the Nonadiabatic Photoinduced Electron Transfer Mechanism in Light-Sensing Cryptochrome
Source: ACS Cent Sci. 2025 May 30;11(7):1071–82. doi: 10.1021/acscentsci.5c00376 (PMC12291142; doi:10.1021/acscentsci.5c00376)
Supplement: Supplementary file 2 [file oc5c00376_si_002.pdf]

oc-2025-00376z.R1

Name: Peer Review Information for "Decrypting the Non-Adiabatic Photoinduced Electron Transfer Mechanism in Light-Sensing Cryptochrome"

First Round of Reviewer Comments

Reviewer: 1

Comments to the Author

The manuscript by Costa and Liang reports on a detailed study of the phototriggered electron transfer in the cryptochrome, based on various theoretical approaches ranging from non-adiabatic to adiabatic dynamics within a QM/MM framework and a multiconfigurational method for the electronic structure. The authors clearly describe the system and its important active center, and how they built their QM/MM model with a QM region containing the FAD (flavin adenine dinucleotide) and two other residues (tryptophan 400, W400, and aspartic acid 396, D396). The choice of this QM region is dictated by the process studied in this work: the electron transfer from the nearby W400 to the photoexcited FAD. The authors used non-adiabatic dynamics to understand the importance of multiple electronic states for this electron-transfer process, and adiabatic dynamics to actually observe its mechanism (within the complex environment of the protein, treated at the MM level).

This work displays an impressive use of various computational techniques to study a phototriggered electron transfer in a protein environment. I would recommend publication of a revised version of this work after the authors address the following comments and questions.

My central concern with this manuscript is the lack of details about the benchmarking strategy for the simulations presented. I have a series of questions related to this concern:

I could not find a clear justification in the main text or the SI about the active space chosen for both XMS-CASPT2 and SA-CASSCF. The authors only comment in the SI that "Our choice of a medium-sized active space was designed to maintain consistency with the non-adiabatic dynamics and excited-state dynamics simulations, which were performed with the SA(4)-CASSCF(6,6)/6-31G/MM method (see below).", but this statement is not convincing in itself knowing that an active space providing accurate results with XMS-CASPT2 may not be adequate for SA-CASSCF (see doi.org/10.1021/jp907111u and its SI, for example). The authors should bring a stronger validation for the quality of their active space. The authors may want to treat their QM region in isolation to compare excitation energies with single-reference methods like CC2 or higher.

*The authors used SA-CASSCF for their dynamics and benchmarked it with XMS-CASPT2 for the excitation energies. However, I could not find any specific information where the authors would compare XMS-CASPT2 and SA-CASSCF for a set of characteristic geometries, presenting the vertical excitation energies, oscillator strengths and electronic characters of the states considered between the two methods for each structure (not just a comparison over an average of geometries). This analysis would identify any state ordering issue.

*The authors should provide a benchmark of SA-CASSCF (with XMS-CASPT2) away from ground-state structures, for example using the pathways depicted in Figure 4. They mention on page 11 that "The S2 -> S1 decay is mediated by the S2/S1 CI seam. The minimal energy conical intersection (MECI) of the S2 and S1 states is ~0.2-0.6 eV lower energy than the S2 state energy on the S0-state optimized FC points, based on the five ICs we tested. The RMSD between the FC and S2/S1 MECI for the FWD complex is small, in the range of 0.06-0.1 Å": did they optimize the S2/S1 MECIs from these five ICs? If yes, this could offer another opportunity to benchmark SA-CASSCF against XMS-CASPT2.

*How stable was the active space selected along the dynamics? The authors do not mention their threshold for total energy conservation.

*The authors appear to use a different version of ab initio multiple spawning including stochastic selections (SS-AIMS). The authors should provide more information about this

technique in the computational details of the main text and its limitations with respect to the original ab initio multiple spawning (in place of a benchmark for the method), as some types of photochemistry may not be as accurately described (see doi.org/10.1063/5.0045572), for the reader to be aware of the level of theory employed for the dynamics. Did the authors use the overlap or the energy version of SS-AIMS? The SI is unclear about that, as the authors mention 'overlap between every pair of TBFs' but give a unit to the threshold employed (an overlap would not have a unit).

*How did the authors calculate the Hamiltonian matrix elements between TBFs in their SS-AIMS simulations? Did they use a saddle-point approximation or the bra-ket average Taylor expansion introduced in doi.org/10.1063/1.4891530?

*The authors started their excited-state dynamics using initial coordinates and velocities coming from a ground-state QM/MM dynamics, which I believe is done with DFT (i.e., no re-equilibration of the QM/MM ground-state dynamics with SA-CASSCF after the QM/MM DFT dynamics). As the excited-state dynamics are done with SA-CASSCF, can this sudden change of electronic-structure method induce artifacts? How different are the geometries provided by DFT and SA-CASSCF? Given the lack of dynamic correlation for the latter, one could expect bond lengths to be slightly different – could that affect the electronic-state orderings?

*The authors should comment on the impact of ZPE (and its associated broader distribution for geometrical parameters) on the calculation of their absorption spectra, given that their sampling was conducted at 300K (see doi.org/10.1002/qua.25049).

*On page 9, the authors mentioned that the blue shift they observed for the absorption wavelength is similar to that observed with TD-DFT and wB97X-D functional. However, the long-range corrected functional is expected to provide a blue shift for valence transitions.

*The authors mention that they start the adiabatic dynamics from the TBF centroids at the end of the SS-AIMS dynamics. Do they mean the center of each TBF, or the centroid between TBFs?

Reviewer: 2

Comments to the Author

This is a well executed and well written contribution to ACS Central Science that deserves to be published after some revision. The central results is the computational demonstration for a fully non-adiabatic photo-induced intermolecular electron transfer occurring in the protein environment of the reference cryptochrome from Arabidopsis Thaliana. In fact, it is shown that the electron transfer (ET) process from an internal tryptophan residue (W400) to a photo-excited flavin chromophore (FAD) occurs via decay along the S2/S1 intersection space, ultimately yielding a radical-cation (W400 radical cation) radical-anion (FAD radical anion) pair. This result is at variance with the generally accepted mechanism that pointed to an S1 adiabatic electron transfer.

While producing the revised version of the manuscript the authors should deal with the following points:

1) General Comment I. It is possible that the author have overlooked an important aspect of their work. In fact, in my view, the results point to a system designed to suppress the adiabatic ET on S1 that is only performed via a photochemical ET process starting in S2. So the S2 should be a "way" to selectively populate the CT region in S1 leading to a fine control. This could be linked to two factors: i) percentage of S0 to S2 excitations and ii) suppression of S1 thermal effects: i.e. it is possible that the S2 path is designed to ovoid the thermal/vibrational effects affecting an S1 adiabatic mechanism. In other words, avoiding to populate the CT state in S1 thermally would provide a noisy ET dynamics that is avoided, almost by definition, starting on S2. This is reminiscent of the suppression of the thermal noise (most notably on S0) documented in visual pigments.

2) General Comment II. The reader is not informed about the the experimental study of the investigated ET process. Is there any study clearly supporting an adiabatic ET in S1? Why the experimental observations would be still consistent with a non-adiabatic production of the CT state?

3) Page 6, line 56. The ET process must lead to a FAD radical anion (not cation) and a Tryptophan radical cation (not anion). So it should be [FAD. - and W400 . +]. Correct throughout the manuscript.

4) Figure 2. The S3 curve (black) is missing.

5) Page 8. IC needs to be defined here and not in page 11. Notice that IC can be confused with Internal Conversion.

6) Page 9. The criteria for selecting a 6,6 active space must be explicitly discussed, as this is strongly approximated CAS space for FAD. A comment about the basis set should also be given as the 6-31G* basis is relatively simple. It is also necessary to mention which level of state averaging has been used in the CASSCF calculations.

7) Page 9. It is not completely clear how the authors distinguish the electronic character of the S1 state minima. Are they using the charge distribution and dipole moments?

8) Page 12. The acronym TBF has to be defined.

9) Figure 4. Is the total estimated barrier for the thermally driven transformation of LE to CT in S1 (i.e. adiabatically) ca. 6 kcal mol? How does this barrier, that would certainly slow down the thermal adiabatic process, compete in speed with the second ET occurring from a different tryptophan to W400 .+ ?

10) Page 15. It is important to characterize the S2/S1 conical intersection to plot the dipole moment along a circle centered on the MECI intersection. The author should see a continuous change from lower to higher dipole moments.

11) Page 15. Define NEB.

12) Figure 4. What are the differences between CASSCF and XMS-CASPT2 in terms of energy profiles? At which level the adiabatic barrier is computed? How does the dynamic electron correlation affect the barrier? Why not to do single point corrections? This has to be discussed in the main text.

13) Page 18. Is it then concluded that the system that upon excitation reach S1 never reach the CT minimum?

14) Figure 5. The figure shows a surface touching (i.e. a degeneracy between two smooth potential energy surfaces. In reality conical intersections are real crossing and the point of crossing is a cusp on both S1 and S2. Please correct.

15) I am not convinced that the real motivation for a protein environment stopping the adiabatic LE->CT process is connected to the need for making the next ET step more efficient or even possible. While this remains a possibility based on the qualitative model presented in the manuscript, the author should also consider other hypotheses (see also above).

Author's Response to Peer Review Comments:

Texas Tech University
Department of Chemistry and Biochemistry
1204 Boston Avenue
Lubbock, Texas 79409-1061

Ruibin Liang
Assistant Professor
806-834-7183
rliang@ttu.edu

Apr 16th, 2025

Senior Editor
ACS Central Science

Dear Editor,

We thank the reviewers for the comments. We have addressed the comments as detailed below. The reviewer's comments are shown in green and modified texts in the revised manuscript are shown in blue. The page numbers mentioned in our response refer to the track-change version of the manuscript uploaded as a supplementary document for reviewers. Table and Figure numbers refere to the revised version of the main text and SI. Reference numbers refer to this response letter.

Reviewer 1

The manuscript by Costa and Liang reports on a detailed study of the phototriggered electron transfer in the cryptochrome, based on various theoretical approaches ranging from non-adiabatic to adiabatic dynamics within a QM/MM framework and a multiconfigurational method for the electronic structure. The authors clearly describe the system and its important active center, and how they built their QM/MM model with a QM region containing the FAD (flavin adenine

dinucleotide) and two other residues (tryptophan 400, W400, and aspartic acid 396, D396). The choice of this QM region is dictated by the process studied in this work: the electron transfer from the nearby W400 to the photoexcited FAD. The authors used non-adiabatic dynamics to

Texas Tech University
Department of Chemistry and Biochemistry
1204 Boston Avenue
Lubbock, Texas 79409-1061

Ruibin Liang
Assistant Professor
806-834-7183
rliang@ttu.edu

understand the importance of multiple electronic states for this electron-transfer process, and adiabatic dynamics to actually observe its mechanism (within the complex environment of the protein, treated at the MM level).

This work displays an impressive use of various computational techniques to study a phototriggered electron transfer in a protein environment. I would recommend publication of a revised version of this work after the authors address the following comments and questions.

My central concern with this manuscript is the lack of details about the benchmarking strategy for the simulations presented. I have a series of questions related to this concern:

We thank the reviewer for recognizing the significance and quality of our work and raising the concerns of benchmarking strategy.

Specific comments:

1) I could not find a clear justification in the main text or the SI about the active space chosen for both XMS-CASPT2 and SA-CASSCF. The authors only comment in the SI that "Our choice of a medium-sized active space was designed to maintain consistency with the non-adiabatic dynamics and excited-state dynamics simulations, which were performed with the SA(4)-CASSCF(6,6)/6-31G*/MM method (see below).", but this statement is not convincing in itself knowing that an active space providing accurate results with XMS-CASPT2 may not be adequate for SA-CASSCF (see doi.org/10.1021/jp907111u and its SI, for example). The authors should bring a stronger validation for the quality of their active space. The authors may want to treat their QM region in isolation to compare excitation energies with single-reference methods like CC2 or higher.

Response:

We thank the reviewer for raising this important point. In our investigation of the photoinduced ET in *At*CRY1 using SA-CASSCF, we employed a 6-electron, 6-orbital (i.e., (6e,6o)) active space, with state averaging over four lowest singlet states. This active space was selected

Texas Tech University
Department of Chemistry and Biochemistry
1204 Boston Avenue
Lubbock, Texas 79409-1061

Ruibin Liang
Assistant Professor
806-834-7183
rliang@ttu.edu

to capture the key electronic processes governing the reaction mechanism, specifically the intramolecular π→π* local excitation (LE) within the FAD moiety and the intermolecular π→π* charge-transfer (CT) excitation from the nearest tryptophan residue (W400) to FAD. This active space was chosen in consideration of cost-accuracy trade-off for non-adiabatic and adiabatic dynamics simulations. The chosen active space effectively encompasses the essential orbitals involved in the formation of the radical pair [$FAD^{\bullet-}$ + $W400^{\bullet+}$]. Also, it is larger than the (4e, 3o) active space in the SA-CASSCF optimizations performed by Solov'yov et. al[1] (*J. Am. Chem. Soc.* **2012**, *134* (43), 18046-18052.) when describing the same photoinduced ET step in *At*CRY1.

To further benchmark the robustness of the SA-4-CASSCF(6,6) active space, we extracted 5 snapshots of the FDW complex from the protein environment sampled in the FC region by ground-state QM/MM MD simulations, and performed single-point energy calculations in the vacuum, and calculated its single-point energies of ground and excited states using different wavefunction-based methods, as summarized in **Table S2**. The benchmark methods include extended multi-state CASPT2 (XMS-CASPT2) with (6e,6o) and (8e,8o) active spaces, algebraic diagrammatic construction to second order (ADC(2)), and approximate coupled-cluster singles and doubles (CC2). All calculations were carried out in the vacuum using the 6-31G* basis set. The ADC(2) and CC2 calculations were performed using the TURBOMOLE package (version 7.7). The SA-CASSCF and XMS-CASPT2 calculations were carried out with the OpenMolcas package.

The results in **Table S2** show that methods incorporating dynamic electron correlation exhibit trends consistent with SA-4-CASSCF(6,6). As expected, the excitation energies obtained with SA-CASSCF are blue-shifted relative to those computed with XMS-CASPT2, CC2 and ADC(2), which includes dynamic correlation. However, the nature of the S_1 and S_2 states (i.e., CT or LE) as well as their ordering predicted by SA-4-CASSCF(6,6) are consistent with most of the benchmark methods, validating one of our key conclusions that in the Franck-Condon region, the S_1 and S_2 states can adopt CT and LE characters, respectively.

Texas Tech University
Department of Chemistry and Biochemistry
1204 Boston Avenue
Lubbock, Texas 79409-1061

Ruibin Liang
Assistant Professor
806-834-7183
rliang@ttu.edu

Table S2. Benchmarking the SA-CASSCF method against higher-level wavefunction methods (XMS-CASPT2, ADC(2) and CC2), incorporating dynamic electron correlations. The excitation energies (ΔE, in eV), oscillator strengths (f), and the nature of the excited states are calculated for the FDW complex in the vacuum. The structures were sampled by ground-state QM/MM MD equilibration simulations with DFT(ωPBEh) as QM method. Two active spaces were tested for both SA-CASSCF and XMS-CASPT2, i.e., (6e,6o) and (8e,8o), with state averaging over four electronic states. Results obtained with the (8e,8o) active space are reported in **bold** font below the results of (6e,6o) in normal font. All calculations employed the 6-31G* basis set. The characters of the adiabatic excited states were assigned based on inspection of the molecular orbitals involved in electronic transition.

**Geometry/ State**	**SA-CASSCF**			**XMS-CASPT2**			**ADC(2)**			**CC2**		
	ΔE	*f*	Nat.	ΔE	*f*	Nat.	ΔE	*f*	Nat.	ΔE	*f*	Nat.
**Geom 1**												
S_1	3.60	0.00	CT	3.07	0.00	CT	2.92	0.00	CT	3.03	0.00	CT
	**3.07**	**0.00**	**CT**	**3.41**	**0.00**	**CT**						
S_2	4.12	0.40	LE	3.46	0.58	LE	3.07	0.28	LE	3.20	0.27	LE
	**4.13**	**0.51**	**LE**	**3.48**	**0.39**	**LE**						
S_3	4.89	0.65	LE	4.35	0.17	LE	3.49	0.00	LE	3.55	0.00	LE
	**4.56**	**0.00**	**LE**	**4.55**	**0.00**	**LE**						
**Geom 2**												
S_1	3.55	0.00	CT	2.90	0.00	CT	2.75	0.00	CT	2.89	0.00	CT
	**3.51**	**0.00**	**CT**	**3.00**	**0.00**	**LE**						
S_2	4.05	0.26	LE	3.32	0.56	LE	2.81	0.24	LE	2.98	0.24	LE
	**4.05**	**0.25**	**LE**	**3.34**	**0.56**	**LE**						
S_3	4.73	0.76	LE	4.18	0.15	LE	3.41	0.01	LE	3.57	0.00	LE
	**4.73**	**0.76**	**LE**	**4.19**	**0.15**	**LE**						
**Geom 3**												
S_1	3.54	0.00	CT	3.01	0.00	CT	2.76	0.18	CT	2.90	0.18	CT
	**3.52**	**0.00**	**CT**	**3.08**	**0.00**	**CT**						
S_2	3.63	0.20	LE	3.27	0.35	LE	2.84	0.00	LE	2.98	0.00	LE
	**3.63**	**0.20**	**LE**	**3.28**	**0.35**	**LE**						
S_3	4.71	0.70	LE	4.27	0.29	LE	3.49	0.00	LE	3.54	0.00	LE
	**4.71**	**0.70**	**LE**	**4.28**	**0.30**	**LE**						
**Geom 4**												
S_1	3.74	0.00	CT	3.26	0.00	CT	2.97	0.27	LE	3.13	0.27	LE
	**3.68**	**0.00**	**CT**	**3.40**	**0.44**	**CT**						
S_2	3.96	0.27	LE	3.38	0.46	LE	3.09	0.00	CT	3.22	0.00	CT
	**3.96**	**0.26**	**LE**	**3.41**	**0.02**	**LE**						
S_3	4.90	0.73	LE	4.34	0.21	LE	3.57	0.00	LE	3.61	0.00	LE
	**4.84**	**0.66**	**LE**	**4.36**	**0.22**	**LE**						
**Geom 5**												
S_1	3.58	0.28	CT	2.84	0.44	CT	2.63	0.20	CT	2.78	0.20	CT
	**3.97**	**0.42**	**CT**	**2.96**	**0.30**	**CT**						
S_2	4.38	0.00	LE	3.18	0.00	LE	2.92	0.00	LE	3.05	0.00	LE

Texas Tech University
Department of Chemistry and Biochemistry
1204 Boston Avenue
Lubbock, Texas 79409-1061

Ruibin Liang
Assistant Professor
806-834-7183
rliang@ttu.edu

	**4.06**	**0.00**	**LE**	**3.27**	**0.00**	**LE**						
S_3	4.88	0.65	LE	4.09	0.19	LE	3.30	0.00	CT	3.39	0.00	CT
	**4.53**	**0.01**	**LE**	**3.42**	**0.01**	**LE**						

Revisions:

The above discussion and data are added to the SI pages S20-S23.

2) The authors used SA-CASSCF for their dynamics and benchmarked it with XMS-CASPT2 for the excitation energies. However, I could not find any specific information where the authors would compare XMS-CASPT2 and SA-CASSCF for a set of characteristic geometries, presenting the vertical excitation energies, oscillator strengths and electronic characters of the states considered between the two methods for each structure (not just a comparison over an average of geometries). This analysis would identify any state ordering issue.

Response:

We performed additional QM/MM benchmark calculations comparing the excitation energy, oscillator strength and nature of the excited states for a new set of geometries in the FC region calculated at the XMS-CASPT2 and SA-CASSCF levels of theory, with both (6e,6o) and (8e, 8o) active spaces. The results are summarized in **Table S3**. Different from the data in **Table S2**, these calculations were performed in the QM/MM settings, and the QM region included the FWD complex.

As expected, including dynamic electron correlation (XMS-CASPT2) results in a redshift in the excitation energies compared to SA-CASSCF. However, it is important to note that the ordering of the excited states predicted by SA-CASSCF remains consistent upon XMS-CASPT2 energy correction. This state ordering is particularly robust in two representative cases: (1) the S_1 and S_2 state adopts LE and CT characters, respectively, and (2) the S_1 and S_2 state adopts CT and LE characters, respectively. These QM/MM benchmarks further validate the reliability of the SA-CASSCF wavefunction for treating photoinduced ET in *At*CRY1.

Texas Tech University
Department of Chemistry and Biochemistry
1204 Boston Avenue
Lubbock, Texas 79409-1061

Ruibin Liang
Assistant Professor
806-834-7183
rliang@ttu.edu

Table S3. Benchmarking the SA-CASSCF/MM method against the XMS-CASPT2/MM method. The geometries were sampled from the ground-state QM/MM MD equilibration in the FC region. The excitation energy (ΔE, in eV) and oscillator strengths (*f*), and the nature of the excited states (LE or CT) are compared.

Geometry/ State	SA-CASSCF(6,6)			XMS-CASPT2(6,6)			SA-CASSCF(8,8)			XMS-CASPT2(8,8)		
	ΔE	*f*	Nat.	ΔE	*f*	Nat.	ΔE	*f*	Nat.	ΔE	*f*	Nat.
**Geom 1**												
S_1	2.97	0.00	LE	2.82	0.00	LE	3.22	0.00	LE	2.89	0.00	LE
S_2	4.66	1.13	CT	3.24	0.75	CT	4.26	0.94	CT	3.24	0.71	CT
S_3	5.76	0.00	LE	5.00	0.00	LE	5.85	0.00	LE	4.83	0.00	LE
**Geom 2**												
S_1	4.60	0.63	LE	3.20	0.48	LE	4.40	0.82	LE	3.15	0.46	LE
S_2	5.12	0.02	CT	3.49	0.00	CT	5.18	0.00	CT	3.57	0.00	CT
S_3	6.17	0.55	LE	4.36	0.23	LE	6.28	0.06	LE	4.39	0.16	LE
**Geom 3**												
S_1	3.74	0.00	CT	2.97	0.00	CT	3.66	0.00	CT	2.95	0.00	CT
S_2	3.88	0.31	LE	3.17	0.49	LE	3.88	0.31	LE	3.19	0.49	LE
S_3	4.79	0.67	LE	4.22	0.21	LE	4.79	0.68	LE	4.23	0.22	LE
**Geom 4**												
S_1	3.75	0.15	LE	3.35	0.19	LE	3.75	0.15	LE	3.36	0.19	LE
S_2	3.94	0.00	CT	3.49	0.00	CT	3.89	0.00	CT	3.55	0.00	CT
S_3	4.80	0.53	LE	4.56	0.32	LE	4.80	0.53	LE	4.57	0.32	LE
**Geom 5**												
S_1	3.73	0.18	LE	3.21	0.44	LE	3.73	0.18	LE	3.21	0.43	LE
S_2	4.06	0.00	CT	3.47	0.00	CT	3.97	0.00	CT	3.55	0.00	CT
S_3	4.74	0.78	LE	4.05	0.24	LE	4.74	0.78	LE	4.07	0.24	LE

Revisions:

The above discussion and data are added to the SI pages S20-S23.

3) The authors should provide a benchmark of SA-CASSCF (with XMS-CASPT2) away from ground-state structures, for example using the pathways depicted in Figure 4. They mention on page 11 that "The S2 -> S1 decay is mediated by the S2/S1 CI seam. The minimal energy conical intersection (MECI) of the S2 and S1 states is ~0.2-0.6 eV lower energy than the S2 state energy on the S0-state optimized FC points, based on the five ICs we tested. The RMSD between the FC and S2/S1 MECI for the FWD complex is small, in the range of 0.06-0.1 Å": did they optimize the S2/S1 MECIs from these five ICs? If yes, this could offer another opportunity to benchmark SA-CASSCF against XMS-CASPT2.

Texas Tech University
Department of Chemistry and Biochemistry
1204 Boston Avenue
Lubbock, Texas 79409-1061

Ruibin Liang
Assistant Professor
806-834-7183
rliang@ttu.edu

Response:

We extended our benchmark analysis by XMS-CASPT2 single-point energy calculations at geometries located beyond the FC region. Single-point energy calculations were carried out at XMS-CASPT2/SA-4-CASSCF(8,8)/6-31G*/MM level of theory along the S_1-state minimum energy pathways (MEPs) from the low-energy LE minimum (LE_{low}) to the high-energy LE minimum (LE_{high}), as well as from LE_{high} to the CT minimum. Note that the three minima and two MEPs were optimized on the S_1 state at the SA-CASSCF(6,6)/6-31G*/MM level of theory. **Figures S8** and **S9** display the XMS-CASPT2/MM PES along the LE_{low} → LE_{high} and, LE_{high} → CT minimum pathways, respectively.

After the XMS-CASPT2 energy corrections, we observe S_1-state PESs similar to those predicted by the SA-CASSCF method. The MEP from LE_{low} to LE_{high} is endergonic by ~3.5 kcal/mol (**Figure S8**), compared to ~5 kcal/mol predicted at the SA-CASSCF(6,6) level of theory (**Figure 4A**). The MEP from LE_{high} to CT minimum is exergonic by ~7 kcal/mol as predicted by XMS-CASPT2 (**Figure S9**), compared to an exergonicity of ~4.8 kcal/mol predicted by SA-CASSCF. Both methods predict a nearly barrierless pathway from LE_{high} to CT minimum. It is noteworthy that because the LE_{high} → CT minimum pathway changes state character through the transition state, and different levels of theories predict slightly different structures of this transition state, the position along the NEB path where the wavefunction switches from the LE to the CT character differ between the two methods (**Figure 4B vs. Figure S9**). This discrepancy, however, does not affect the main conclusion that the LE_{high} → CT pathway is mostly barrierless and exergonic.

Texas Tech University
Department of Chemistry and Biochemistry
1204 Boston Avenue
Lubbock, Texas 79409-1061

Ruibin Liang
Assistant Professor
806-834-7183
rliang@ttu.edu

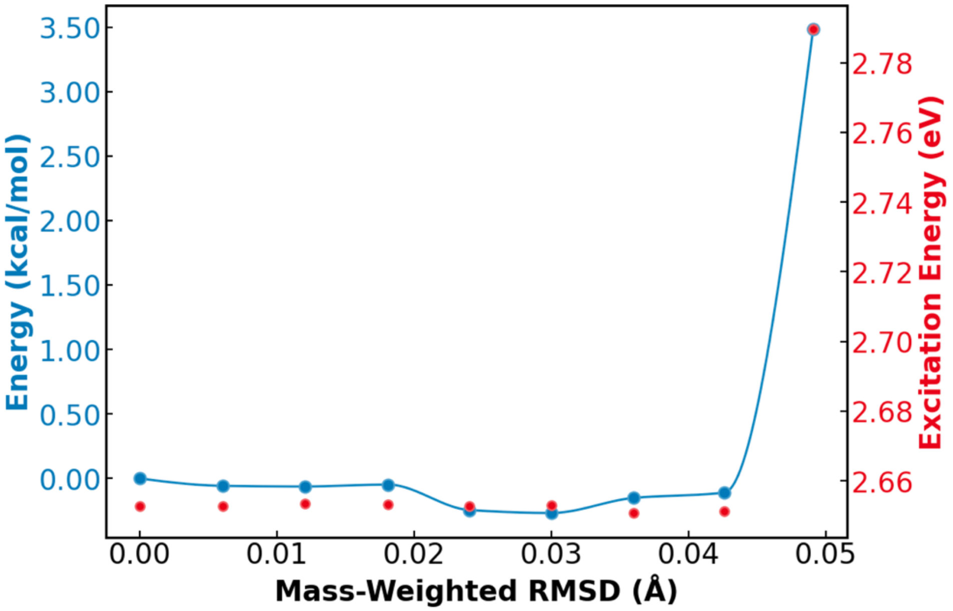

Figure S8. PES along the MEP on the S_1 surface from the low-energy LE minimum (left) and high-energy LE minimum (right). Blue curves and dots represent the energies, while red dots indicate the S_1–S_0 energy gaps. The MEP was optimized at the SA-4-CASSCF(6,6)/6-31G*/MM level, and energies were recalculated on the images along this MEP at the XMS-CASPT2/SA-4-CASSCF(8,8)/6-31G*/MM level of theory.

Texas Tech University
Department of Chemistry and Biochemistry
1204 Boston Avenue
Lubbock, Texas 79409-1061

Ruibin Liang
Assistant Professor
806-834-7183
rliang@ttu.edu

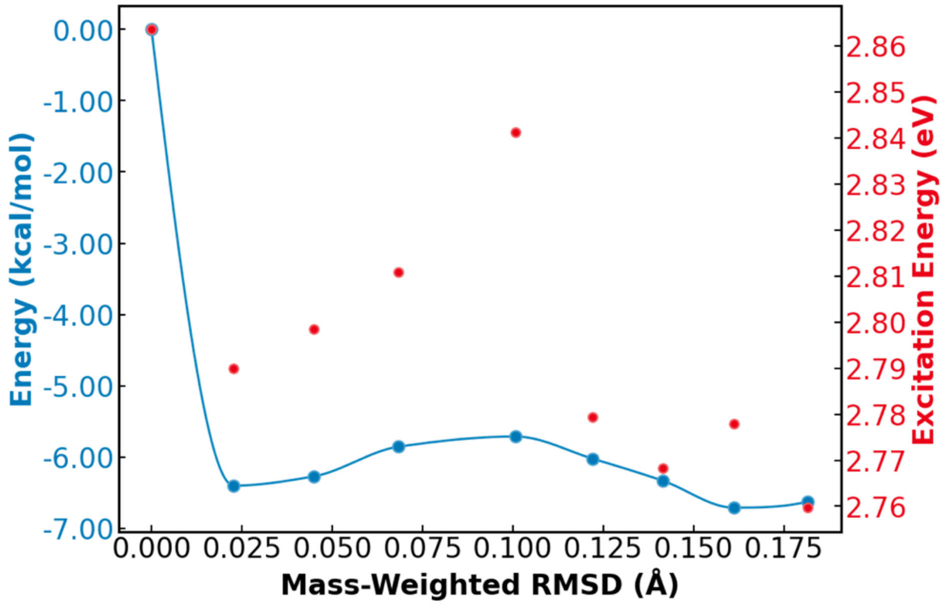

Figure S9. PES along the MEP on the S_1 surface from the high-energy LE minima (left) to the CT minima (right). Blue curves and dots represent the energies, while red dots indicate the S_1–S_0 energy gaps. The MEP was optimized at the SA-4-CASSCF(6,6)/6-31G*/MM level, and energies were recalculated on the images along this MEP at the XMS-CASPT2/SA-4-CASSCF(8,8)/6-31G*/MM level of theory.

In addition, single-point energy calculations were carried out at XMS-CASPT2/SA-4-CASSCF(8,8)/6-31G*/MM level of theory at the minimum energy conical intersections (MECI) between the S_2 and S_1 states (S_2/S_1 MECI), which were optimized at the SA-CASSCF(6,6)/6-31G*/MM level of theory (**Table S4**). The S_2 energy of the optimized FC points is higher than the energy at the S_2/S_1 MECI, confirming the conclusions from the SA-CASSCF calculations.

Table S4. XMS-CASPT2/MM energies at the S_2/S_1 MECIs and their relative energies with respect to the FC geometries. The MECIs were optimized at SA-4-CASSCF(6,6)/6-31G*/MM level of theory, and the XMS-CASPT2/MM single-point energies were calculated on top of the MECI structures. The XMS-CASPT2 calculations were performed using SA-4-CASSCF(6,6) and SA-4-CASSCF(8,8) reference wavefunctions and 6-31G* basis set. The results obtained from the (8,8) active space are highlighted in bold font, and (6,6) active space in normal font. For each geometry, the energy difference between the Franck-Condon point and the S_2/S_1 MECI (**ΔE(FC→MECI)**)

Texas Tech University
Department of Chemistry and Biochemistry
1204 Boston Avenue
Lubbock, Texas 79409-1061

Ruibin Liang
Assistant Professor
806-834-7183
rliang@ttu.edu

was computed using the S_2 energy at the FC point and the average energy between S_1 and S_2 states at the MECI.

**Geometry/State**	**Franck-Condon**				**S_2/S_1 MECI**			**ΔE(FC→MECI) (eV)**
	**E (a.u.)**	**ΔE (eV)**	***f***	**Nat.**	**E (a.u.)**	**ΔE (eV)**	**Nat.**	
**Geom 1**								
S_0	-1660.58857				-1660.56986			
	**-1660.58683**				**-1660.57085**			
S_1	-1660.47494	3.09	0.00	CT	-1660.48508	2.31	CT	
	**-1660.46312**	**3.37**	**0.01**	**CT**	**-1660.48401**	**2.36**	**CT**	
S_2	-1660.46986	3.23	0.50	LE	-1660.46931	2.74	LE	0.20
	**-1660.46270**	**3.38**	**0.17**	**LE**	**-1660.47003**	**2.74**	**LE**	**0.39**
S_3	-1660.43857	4.08	0.21	LE	-1660.42453	3.95	LE	
	**-1660.44636**	**3.82**	**0.00**	**CT**	**-1660.42484**	**3.97**	**LE**	
**Geom 2**								
S_0	-1658.50964				-1658.49743			
	**-1658.51317**				**-1658.49469**			
S_1	-1658.39089	3.23	0.00	CT	-1658.40263	2.58	CT	
	**-1658.39555**	**3.20**	**0.20**	**LE**	**-1658.40063**	**2.56**	**CT**	
S_2	-1658.38953	3.27	0.49	LE	-1658.39549	2.77	LE	0.26
	**-1658.38947**	**3.37**	**0.00**	**CT**	**-1658.38945**	**2.86**	**LE**	**0.15**
S_3	-1658.35609	4.18	0.23	LE	-1658.34565	4.13	LE	
	**-1658.35540**	**4.29**	**0.33**	**LE**	**-1658.35408**	**3.83**	**LE**	
**Geom 3**								
S_0	-1657.75063				-1657.73393			
	**-1657.74925**				**-1657.73835**			
S_1	-1657.64063	2.99	0.00	CT	-1657.64294	2.48	LE	
	**-1657.62987**	**3.25**	**0.00**	**CT**	**-1657.63328**	**2.86**	**LE**	
S_2	-1657.62724	3.36	0.49	LE	-1657.62882	2.86	CT	0.24
	**-1657.62138**	**3.48**	**0.22**	**LE**	**-1657.61498**	**3.36**	**CT**	**0.07**
S_3	-1657.59347	4.28	0.23	LE	-1657.55276	4.93	CT	
	**-1657.61083**	**3.77**	**0.00**	**CT**	**-1657.58915**	**4.06**	**CT**	
**Geom 4**								
S_0	-1658.50983				-1658.49662			
	**-1658.51336**				**-1658.49390**			
S_1	-1658.39095	3.23	0.00	CT	-1658.40136	2.59	CT	
	**-1658.39577**	**3.20**	**0.20**	**LE**	**-1658.39936**	**2.57**	**CT**	
S_2	-1658.38977	3.27	0.49	LE	-1658.39469	2.77	LE	0.22
	**-1658.38953**	**3.37**	**0.00**	**CT**	**-1658.38862**	**2.86**	**LE**	**0.12**
S_3	-1658.35622	4.18	0.23	LE	-1658.34503	4.12	LE	
	**-1658.35556**	**4.29**	**0.33**	**LE**	**-1658.35343**	**3.82**	**LE**	
**Geom 5**								
S_0	-1658.51209				-1658.49785			
	**-1658.51308**				**-1658.49459**			
S_1	-1658.39477	3.19	0.20	LE	-1658.40031	2.65	CT	
	**-1658.39550**	**3.20**	**0.20**	**LE**	**-1658.40055**	**2.56**	**CT**	
S_2	-1658.39182	3.27	0.00	CT	-1658.39476	2.81	LE	0.16
	**-1658.38945**	**3.36**	**0.00**	**CT**	**-1658.38937**	**2.86**	**LE**	**0.15**
S_3	-1658.35489	4.28	0.33	LE	-1658.36215	3.69	CT	
	**-1658.35535**	**4.29**	**0.33**	**LE**	**-1658.35407**	**3.82**	**LE**	

Texas Tech University
Department of Chemistry and Biochemistry
1204 Boston Avenue
Lubbock, Texas 79409-1061

Ruibin Liang
Assistant Professor
806-834-7183
rliang@ttu.edu

Revisions:

The above discussion and data are added to the SI pages S23-S26.

4) How stable was the active space selected along the dynamics? The authors do not mention their threshold for total energy conservation.

Response:

We thank the reviewer for the question aimed at improving the reproducibility of our study. In the SSAIMS simulations, the threshold of total energy conservation was set to 0.006 a.u.. 12 out of our 75 SSAIMS runs encountered energy conversation issues, possibly due to active space instability, which are mostly encountered after $S_2 \rightarrow S_1$ decay. In our population analysis in Figure 3A, we extracted population data from each run until either it ended normally or was terminated halfway. In the latter case, the data is extrapolated based on the last time point. Based on our MECI benchmark results (Table S4), we anticipate the overall conclusion that the non-adiabatic ET dynamics finishes in an ultrafast timescale within 30 fs do not change.

Revision:

The following is added to the SI on page S9:

The threshold of total energy conservation was set to 0.006 a.u.

5) The authors appear to use a different version of ab initio multiple spawning including stochastic selections (SS-AIMS). The authors should provide more information about this technique in the computational details of the main text and its limitations with respect to the original ab initio multiple spawning (in place of a benchmark for the method), as some types of photochemistry may not be as accurately described (see doi.org/10.1063/5.0045572), for the reader to be aware of the level of theory employed for the dynamics. Did the authors use the overlap or the energy version

Texas Tech University
Department of Chemistry and Biochemistry
1204 Boston Avenue
Lubbock, Texas 79409-1061

Ruibin Liang
Assistant Professor
806-834-7183
rliang@ttu.edu

of SS-AIMS? The SI is unclear about that, as the authors mention 'overlap between every pair of TBFs' but give a unit to the threshold employed (an overlap would not have a unit).

Response:

We thank the reviewer for pointing out the unit inconsistency and for emphasizing the importance of providing methodological details to ensure reproducibility.

In this study, we employed the Stochastic-Selection Ab Initio Multiple Spawning method, specifically the Overlap-based variant (OSSAIMS), in which stochastic TBF selection events are triggered based on the absolute value of the overlap between TBFs. The overlap threshold is indeed unitless and was set to a value of 0.2. The acronym SS-AIMS has been replaced with OSSAIMS throughout the manuscript and SI to reflect the specific implementation adopted in our work.

Additionally, the following sentence in the SI was revised to eliminate the previously noted inconsistency: **"The two groups were considered to be completely decoupled when the overlap between every pair of TBFs, each selected from one group"**

A detailed description of the SS-AIMS methodology, including its implementation and differences from the original AIMS algorithm, has been incorporated into the Computational Details section of the revised manuscript.

Revisions:

The following text is added to the main text in the Summary of Computational Method, pages 28-29:

Stochastic-Selection Ab Initio Multiple Spawning (SSAIMS)[2, 3] is a variant of the original deterministic Ab Initio Multiple Spawning (AIMS) algorithm. It introduces a stochastic selection procedure to reduce the number of TBFs being simultaneously propagated and, consequently, significantly saves computational costs. At each time step, the method evaluates the coupling

Texas Tech University
Department of Chemistry and Biochemistry
1204 Boston Avenue
Lubbock, Texas 79409-1061

Ruibin Liang
Assistant Professor
806-834-7183
rliang@ttu.edu

between two TBFs and identifies groups that have become effectively decoupled based on a predefined decoupling threshold. A stochastic procedure is then used to select, based on the total population of each uncoupled group, one of the uncoupled groups to continue the propagation while the others are terminated. This strategy controls the growth in the number of TBFs while preserving the essential dynamical details described by the reference AIMS simulations[2, 3]. Benchmark studies[2, 3] have shown that defining the decoupling threshold based on TBF overlaps (referred to as OSSAIMS) best agrees with the deterministic reference AIMS dynamics for the smallest average number of TBFs, so this implementation was employed here. Due to stochastic selections of TBFs, multiple independent SSAIMS simulations need to be launched from each IC using different random seeds such that the results statistically converge to the reference AIMS simulations. We performed 5 independent runs for each IC.

6) How did the authors calculate the Hamiltonian matrix elements between TBFs in their SS-AIMS simulations? Did they use a saddle-point approximation or the bra-ket average Taylor expansion introduced in doi.org/10.1063/1.4891530?

Response:

The saddle point approximation was employed to calculate the Hamiltonian matrix elements.

Revisions:

The above discussion is added to the SI, page S9.

7) The authors started their excited-state dynamics using initial coordinates and velocities coming from a ground-state QM/MM dynamics, which I believe is done with DFT (i.e., no re-equilibration of the QM/MM ground-state dynamics with SA-CASSCF after the QM/MM DFT dynamics). As the excited-state dynamics are done with SA-CASSCF, can this sudden change of electronic-structure method induce artifacts? How different are the geometries provided by DFT and SA-

Texas Tech University
Department of Chemistry and Biochemistry
1204 Boston Avenue
Lubbock, Texas 79409-1061

Ruibin Liang
Assistant Professor
806-834-7183
rliang@ttu.edu

CASSCF? Given the lack of dynamic correlation for the latter, one could expect bond lengths to be slightly different – could that affect the electronic-state orderings?

Response:

Since the FC region was sampled by ground-state DFT/MM MD simulations whereas the photodynamics were propagated by the SA-CASSCF method, it is important to evaluate if SA-CASSCF and the DFT (ωPBEh) methods predict similar molecular structures with comparable electronic properties of the excited states. To this end, we carried out constrained ground-state and S_1-state geometry optimizations of the FWD complex in the vacuum, starting from the experimental crystal structure (PDB ID: 1U3C). Given the inherent flexibility of the system, we optimized the geometries sequentially for each individual molecular component in the following order: (1) the FAD moiety, (2) the W400 and (3) the D396 residues. Subsequently, electronic excitation properties, such as excitation energy (ΔE), oscillator strength (f), dipole moment (μ), and the nature of the state were recalculated at SA-4-CASSCF(6,6) level of theory based on these optimized structures (**Table S5**). The geometric parameters (bonds, angles and dihedrals) of the optimized by different methods are also compared in **Table S6.** In our test, using different methods to optimize the FC geometries on the ground state results in similar excitation energies and same state ordering, with minimal differences in the geometric properties.

Table S5. Electronic properties of the FWD complex in vacuum on the top of ground-state FC geometries optimized using SA-4-CASSCF(6,6) and DFT (ωPBEh). The electronic properties were computed at SA-4-CASSCF(6,6). All electronic structure calculations were performed with a 6-31G* atomic basis set in the vacuum.

	SA-CASSCF//SA-CASSCF				SA-CASSCF//DFT(ωPBEh)			
	ΔE	*f*	μ	Nat.	ΔE	*f*	μ	Nat.
S_1	4.28	0.380	10.43	LE	4.07	0.341	10.79	LE
S_2	4.73	0.001	36.10	CT	4.24	0.002	34.08	CT
S_3	5.20	0.674	9.59	LE	5.01	0.674	9.60	LE

Table S6. Comparison of selected bond lengths, bond angles, and dihedral angles of the FDW complex between ground-state and S_1-state constrained-optimized geometries using the SA-4-

Texas Tech University
Department of Chemistry and Biochemistry
1204 Boston Avenue
Lubbock, Texas 79409-1061

Ruibin Liang
Assistant Professor
806-834-7183
rliang@ttu.edu

CASSCF(6,6) and DFT(ωPBEh)/TD-DFT(ωPBEh) methods with the 6-31G* atomic basis set. All optimizations were performed in the vacuum. Bond lengths are reported in angstroms, while angles and dihedral are in degrees. The symbol Δ represents the absolute difference between the values obtained from the two computational approaches.

Geometric Property	S_0				S_1		
	SA-CASSCF	DFT	Δ (S_0)		SA-CASSCF	DFT	Δ (S_1)
**Bond**				**Bond**			
C-O	1.20	1.22	0.02	C-C	1.46	1.39	0.07
C-O	1.32	1.34	0.02	C-N	1.27	1.32	0.04
C-C	1.35	1.37	0.02	N-C	1.41	1.38	0.03
O-C	1.19	1.21	0.02	C-O	1.20	1.22	0.02
C-O	1.19	1.22	0.02	C-O	1.32	1.34	0.02
**Angle**				**Angle**			
C-N-C	120.08	118.21	1.87	C-C-C	117.07	119.80	2.73
C-C-N	124.51	125.51	1.00	C-C-C	123.54	121.67	1.88
N-C-N	120.11	119.18	0.93	O-C-N	122.31	121.00	1.30
C-C-O	125.83	126.60	0.78	O-C-N	120.70	121.97	1.27
C-C-N	119.24	118.49	0.75	C-C-C	116.04	117.11	1.07
**Dihedral**				**Dihedral**			
C-C-N-C	178.48	179.69	1.83	C-C-C-N	179.02	178.41	2.57
C-C-N-C	1.47	0.24	1.71	C-C-C-C	178.48	179.31	2.21
C-C-N-C	2.18	0.61	1.57	C-C-C-C	0.73	1.30	2.02
C-N-C-C	2.18	0.61	1.57	N-C-N-C	0.52	1.41	1.94
C-N-C-N	178.12	179.54	1.41	N-C-C-N	1.05	0.76	1.81

Revisions:

The above discussion and data are added to the SI, pages S27-S28.

8) The authors should comment on the impact of ZPE (and its associated broader distribution for geometrical parameters) on the calculation of their absorption spectra, given that their sampling was conducted at 300K (see doi.org/10.1002/qua.25049).

Response and Revision:

The following is added to the main text, pages 10-11.

Previous work by Barbatti et.al.[4] has reported that incorporation of zero point energy broadens the distribution in the geometries and excitation energy in the absorption spectra

Texas Tech University
Department of Chemistry and Biochemistry
1204 Boston Avenue
Lubbock, Texas 79409-1061

Ruibin Liang
Assistant Professor
806-834-7183
rliang@ttu.edu

compared to sampling according to the Boltzmann distribution at 300 K. We expect the same effect of including ZPE on the absorption spectra in our simulation system. Moreover, since the state ordering is sensitive to the geometries in the FC region, we expect that including ZPE will at least maintain, if not increase, the sampling probability of the ICs with S_1 and S_2 states adopting CT and LE characters, respectively.

9) On page 9, the authors mentioned that the blue shift they observed for the absorption wavelength is similar to that observed with TD-DFT and wB97X-D functional. However, the long-range corrected functional is expected to provide a blue shift for valence transitions.

Response:

We share the reviewer's understanding regarding the known limitations of long-range corrected functionals. In our case, the blue shift observed in the multiconfigurational XMS-CASPT2 calculations may be attributed to the use of a moderately sized active space, CAS(6,6), in combination with the 6-31G* basis set. Due to the relatively large number of electronic structure calculations and the size of the QM region (58 atoms), employing a larger basis set was computationally prohibitive for all 300 structures. However, we anticipate that using a more extended basis set and employing polarizable embedding in the QM/MM calculations[5] would likely lead to a red shift in the excitation energies.

Revision:

The following is added in the main text, page 9:

We anticipate that using a more extended basis set and employing polarizable embedding in the XMS-CASPT2/MM calculations[5] would likely lead to a red shift in the excitation energies in better agreement with the experiment. The effects of enlarging the basis set on the XMS-CASPT2 results are benchmarked in **Table S7**.

Texas Tech University
Department of Chemistry and Biochemistry
1204 Boston Avenue
Lubbock, Texas 79409-1061

Ruibin Liang
Assistant Professor
806-834-7183
rliang@ttu.edu

10) The authors mention that they start the adiabatic dynamics from the TBF centroids at the end of the SS-AIMS dynamics. Do they mean the center of each TBF, or the centroid between TBFs?

We thank the reviewer for this important question. In our simulations, the adiabatic dynamics were initiated from the centroid of each TBF at the end of the OSSAIMS propagation, rather than the centroids of pairs of TBFs. This approach ensures a consistent restart from the SS-AIMS simulation nonadiabatic to the adiabatic regime.

Revisions:

The following is added to the main text, page 29.

Following the SSAIMS simulations, 75 S_1-state adiabatic dynamics simulations were launched, each restarting from the coordinates and velocities of an individual S_1 TBF's centroid that survived at the end of the SSAIMS simulations. The S_1-state trajectories were propagated in the constant NVE ensemble to ensure the continuation of the dynamics from SSAIMS simulations.

Texas Tech University
Department of Chemistry and Biochemistry
1204 Boston Avenue
Lubbock, Texas 79409-1061

Ruibin Liang
Assistant Professor
806-834-7183
rliang@ttu.edu

Reviewer 2

This is a well executed and well written contribution to ACS Central Science that deserves to be published after some revision. The central results is the computational demonstration for a fully non-adiabatic photo-induced intermolecular electron transfer occurring in the protein environment of the reference cryptochrome from Arabidopsis Thaliana. In fact, it is shown that the electron transfer (ET) process from an internal tryptophan residue (W400) to a photo-excited flavin chromophore (FAD) occurs via decay along the S2/S1 intersection space, ultimately yielding a radical-cation (W400 radical cation) radical-anion (FAD radical anion) pair. This result is at variance with the generally accepted mechanism that pointed to an S1 adiabatic electron transfer.

While producing the revised version of the manuscript the authors should deal with the following points:

We thank the reviewer for recognizing the significance and quality of our work.

General Comment I:

It is possible that the author have overlooked an important aspect of their work. In fact, in my view, the results point to a system designed to suppress the adiabatic ET on S1 that is only performed via a photochemical ET process starting in S2. So the S2 should be a "way" to selectively populate the CT region in S1 leading to a fine control. This could be linked to two factors: i) percentage of S0 to S2 excitations and ii) suppression of S1 thermal effects: i.e. it is possible that the S2 path is designed to ovoid the thermal/vibrational effects affecting an S1 adiabatic mechanism. In other words, avoiding to populate the CT state in S1 thermally would provide a noisy ET dynamics that is avoided, almost by definition, starting on S2. This is reminiscent of the suppression of the thermal noise (most notably on S0) documented in visual pigments.

Texas Tech University
Department of Chemistry and Biochemistry
1204 Boston Avenue
Lubbock, Texas 79409-1061

Ruibin Liang
Assistant Professor
806-834-7183
rliang@ttu.edu

Response:

We thank the reviewer for suggesting this interesting perspective on our results. We agree with the reviewer that the non-adiabatic ET dynamics can reduce the thermal noise caused by the S1-state adiabatic barrier crossing, which is analogous to rhodopsins. However, we note the following differences between cryptochrome and rhodopsins. The thermal noise in rhodopsin is inherently low because its ground-state cis-to-trans isomerization has a high activation barrier for thermal isomerization. Its photoisomerization is highly efficient. So when light is present, nearly every cis-to-trans isomerization event is due to photon absorption rather than random thermal events. The net effect is a system with a very high signal-to-noise ratio, not because photoisomerization actively suppresses thermal noises, but because the probability of unwanted thermal events is intrinsically minimized[6]. This design is a key factor in the sensitivity of the vertebrate visual system and is well supported by experimental studies on rod photoreceptors[6-9], which showed that dark noise, although present, is low compared to light-induced responses. In contrast, in the cryptochrome, the adiabatic ET event on the S_1-state is still induced by photoexcitation. The S1-state ET dynamics play an important role since a significant portion of the ICs have the S1 state as LE and the S2 state as CT characters (**Figure 2**). With energy corrections from a higher-level theory, the S1-state barrier is slightly reduced (see our XMS-CASPT2(8,8) benchmark results in **Figure S1**) compared to SA-CASSCF, which can enhance the rate of the adiabatic ET. Consistently, experiments have measured the initial rate of photoinduced ET in cryptochromes and the closely related photolyases to be on the subpicosecond to picosecond timescales[10-12]. In this regard, the ultrafast non-adiabatic ET facilitates the charge separation but does not totally suppress the thermal noise.

Revisions:

The following discussion is added to the main text, page 26.

Texas Tech University
Department of Chemistry and Biochemistry
1204 Boston Avenue
Lubbock, Texas 79409-1061

Ruibin Liang
Assistant Professor
806-834-7183
rliang@ttu.edu

Interestingly, our results indicate that the ultrafast, non-adiabatic ET in cryptochrome can reduce the thermal noise associated with an S_1-state adiabatic barrier crossing. Analogous to rhodopsins, where the high activation barrier for thermal *cis*-to-*trans* isomerization inherently minimizes dark noise and ensures that photoisomerization dominates[6-9], the cryptochrome can leverage the higher-lying LE states and reach the CT minima on the S_1 state through the non-adiabatic pathway, which is faster than through the adiabatic pathway. However, unlike rhodopsin where thermal noise is nearly eliminated, the S1 adiabatic ET in cryptochrome is still non-negligible due to the significant population of conformations being photoexcited to the S_1 state with LE character (**Figure 2**). Also, our benchmarks (**Figures S8 & S9**) show that energy corrections via incorporating dynamic correlations at XMS-CASPT2 level of theory slightly lower the adiabatic S_1-state barrier predicted by the SA-CASSCF approach, which, together with experimental observations of subpicosecond to picosecond ET rates[10-13] of cryptochromes and photolyases, supports a model where ultrafast non-adiabatic ET facilitates charge separation while not entirely suppressing thermal effects.

General Comment II:

The reader is not informed about the experimental study of the investigated ET process. Is there any study clearly supporting an adiabatic ET in S1? Why the experimental observations would be still consistent with a non-adiabatic production of the CT state?

Response and Revision:

The following is added to the main text, page 24:

A previous experimental study by Immeln et al.[11] on *At*CRY1 report an initial photoinduced ET time constant of approximately 0.4 ps, and similar rates (0.5-0.8 ps) have been observed in photolyases[12, 13] and robin cryptochrome 4[10]. Our simulations predict that the non-adiabatic ET event occurs within ~10 fs (**Figure 3A**), which is significantly faster than these experimental time

Texas Tech University
Department of Chemistry and Biochemistry
1204 Boston Avenue
Lubbock, Texas 79409-1061

Ruibin Liang
Assistant Professor
806-834-7183
rliang@ttu.edu

constants. In contrast, our XMS-CASPT2 calculations indicate an S_1-state barrier of about 3.5 kcal/mol for the adiabatic ET process (**Figures S8** & **Figure S9**), which, after accounting for nuclear quantum effects such as tunneling and zero-point energy, aligns well with the experimentally observed timescales. Importantly, transient absorption spectroscopic measurements typically resolve dynamics above ~25 fs, and the ultrafast non-adiabatic ET event is beyond this resolution limit. Moreover, as shown in **Figure 2B**, due to conformational fluctuations in the Franck-Condon region, the excitation energies for $S_0 \rightarrow S_2$ transitions (associated with non-adiabatic ET) overlap with those for $S_0 \rightarrow S_1$ transitions (leading to adiabatic ET), making it difficult to disentangle the contributions of each pathway at the ~445 nm excitation wavelength. Therefore, our findings support that the time constants measured experimentally correspond primarily to the adiabatic ET on the S_1 state, while the rapid non-adiabatic ET remains consistent with, yet unresolved by, current spectroscopic methods.

Specific comments:

1) Page 6, line 56. The ET process must lead to a FAD radical anion (not cation) and a Tryptophan radical cation (not anion). So it should be [FAD. - and W400 . +]. Correct throughout the manuscript.

Response:

We thank the reviewer for pointing out the error in the representation of the radical pair. They are corrected.

Revisions:

Texas Tech University
Department of Chemistry and Biochemistry
1204 Boston Avenue
Lubbock, Texas 79409-1061

Ruibin Liang
Assistant Professor
806-834-7183
rliang@ttu.edu

The radical pair notation is updated in the main text from [$FAD^{\bullet+}$-$W400^{\bullet-}$]. To [$FAD^{\bullet-}$-$W400^{\bullet+}$].

2) Figure 2. The S3 curve (black) is missing.

Response:

We thank the reviewer for the observation. In Figure 2, the color black is used to represent the experimental absorption spectrum. To improve clarity and avoid any potential misinterpretation regarding the excited electronic states, we revised the figure caption accordingly.

Revisions:

- The caption of Figure 2 is updated in the main text:

Figure 2. Absorption spectra and excited-state order of the FWD complex in the *At*CRY1 calculated at the XMS-CASPT2//SA-4-CASSCF(6,6)/6-31G*/MM level of theory and compared with experiment. (A) Comparison between calculated spectrum including all excitation from to the lowest-lying excites states ($S_0 \rightarrow S_1$-S_3) averaged over 300 initial conditions (ICs) sampled on the ground state in the FC region (red curve) with experimental absorption spectrum[24] (black curve) (B) Comparison between the spectrum derived from a subset of 104 ICs (approximately 35%) whose S_2 state has LE character and $S_0 \rightarrow S_1$ transition has higher oscillator strength than $S_0 \rightarrow S_1$ (blue curve) and all ICs (red curve). (C) Energy gap distribution between the lowest-lying singlet adiabatic excited states with the CT and LE characters in *At*CRY1, i.e., $\Delta E = E_{CT}^{lowest} - E_{LE}^{lowest}$. The energy gaps were calculated for the 300 ICs in the FC region using the XMS-CASPT2//SA-4-CASSCF(6,6)/6-31G*/MM method. Energy gaps approaching zero correspond to ICs near the conical intersections between the LE and CT adiabatic states, which is critical for mediating non-adiabatic transitions between them. Negative energy gaps indicate the possibility of photoexcitation to bright LE adiabatic states higher than the CT states, potentially inducing non-adiabatic ET events.

3) Page 8. IC needs to be defined here and not in page 11. Notice that IC can be confused with Internal Conversion.

Texas Tech University
Department of Chemistry and Biochemistry
1204 Boston Avenue
Lubbock, Texas 79409-1061

Ruibin Liang
Assistant Professor
806-834-7183
rliang@ttu.edu

Response:

We thank the reviewer for the observation. The definition of the initial conditions (ICs) is now included at their first occurrence in the manuscript to improve clarity.

Revisions:

The following text has been added to the caption of Figure 2 at the first occurrence of the acronym: initial conditions (ICs)

4) Page 9. The criteria for selecting a 6,6 active space must be explicitly discussed, as this is strongly approximated CAS space for FAD. A comment about the basis set should also be given as the 6-31G* basis is relatively simple. It is also necessary to mention which level of state averaging has been used in the CASSCF calculations.

Response:

We thank the reviewer for the question aimed at improving the reproducibility of our study. Our approach follows prior studies that employed the XMS-CASPT2//SA-CASSCF methodology with principal-orbital active spaces to describe analogous photoinduced charge-separation processes in cryptochromes under a static framework.

In our work, we used a medium-sized active space comprising six electrons in six orbitals, CAS(6,6). This active space was chosen to capture the key electronic features of the mechanism, specifically the intramolecular $\pi \rightarrow \pi^*$ excitation within the flavin adenine dinucleotide (FAD) chromophore and the intermolecular $\pi \rightarrow \pi^*$ electron transfer from the nearest tryptophan residue (W400) to FAD. This choice offers a practical balance between computational cost and accuracy, while including the essential orbitals involved in the formation of the radical pair [$FAD^{\bullet -}$ + $W400^{\bullet +}$].

Texas Tech University
Department of Chemistry and Biochemistry
1204 Boston Avenue
Lubbock, Texas 79409-1061

Ruibin Liang
Assistant Professor
806-834-7183
rliang@ttu.edu

To support the robustness of this selection, we have included benchmark calculations using a different active space CAS(8,8), and two single-reference methods: ADC(2) and CC2. The results are listed in **Table S2** and **Table S3. Please also see our answers to Reviewer 1's comments 1-2 and our revisions therein)** There is a general agreement between the 6,6 active space and 8,8 active space. The state averaging in the CASSCF calculations is over the four lowest singlet states: S_0-S_3.

Revisions:

The following text is added to the SI, together with the **Table S7** on pages S28-S29:

The 6-31G* basis set was chosen based on prior studies to optimize the balance between computational efficiency and theoretical accuracy, especially for extensive dynamics simulations as performed in the current study. This basis set has been successfully employed in previous studies[1, 5, 14] investigating flavin photochemistry, both in vacuum conditions and in complex biological environments. To benchmark the selection of this basis set, we performed SA-CASSCF and XMS-CASPT2 calculations using a larger basis set, ANO-L-VDZP, on the same geometries. The test result is summarized in **Table S7**. Most of the time, using a large basis set preserves the state ordering of the lowest LE and CT adiabatic states and decreases the excitation energy to the LE state. Importantly, with the larger basis set, at both SA-CASSCF and XMS-CASPT2 levels of theory, some geometries have the S_1 state with CT character and S_2 state with LE character, which can contribute to the non-adiabatic ET pathway.

Table S7. Benchmark of effects of basis set on the electronic properties. SA-CASSCF and XMS-CASPT2 calculations were performed on representative FC geometries sampled by the ground-state QM/MM MD equilibration. The multireference calculations were performed using different basis sets, 6-31G* and ANO-L-VDZP, with the latter being the larger one. The results of larger basis set is highlighted in bold font, and smaller basis set in normal font. The nature of electronic nature was verified by the orbitals involved in the electronic transitions and dipole moments.

**Geometry**	**SA-CASSCF(6,6)**			**XMS-CASPT2(6,6)**			**SA-CASSCF(8,8)**			**XMS-CASPT2(8,8)**		
**State**	**ΔE**	***f***	**Nat.**	**ΔE**	***f***	**Nat.**	**ΔE**	***f***	**Nat.**	**ΔE**	***f***	**Nat.**
**Geom 1**												

Texas Tech University
Department of Chemistry and Biochemistry
1204 Boston Avenue
Lubbock, Texas 79409-1061

Ruibin Liang
Assistant Professor
806-834-7183
rliang@ttu.edu

S_1	2.97	0.00	LE	2.82	0.00	LE	3.22	0.00	LE	2.89	0.00	LE
	**2.71**	**0.00**	**CT**	**2.71**	**0.00**	**CT**	**3.71**	**0.00**	**CT**	**2.69**	**0.00**	**CT**
S_2	4.66	1.13	CT	3.24	0.75	CT	4.26	0.94	CT	3.24	0.71	CT
	**3.20**	**0.58**	**LE**	**3.20**	**0.58**	**LE**	**4.19**	**0.30**	**LE**	**3.28**	**0.45**	**LE**
S_3	5.76	0.00	LE	5.00	0.00	LE	5.85	0.00	LE	4.83	0.00	LE
	**3.95**	**0.12**	**LE**	**3.95**	**0.12**	**LE**	**4.20**	**0.50**	**LE**	**3.63**	**0.16**	**LE**
**Geom 2**												
S_1	4.60	0.63	LE	3.20	0.48	LE	4.40	0.82	LE	3.15	0.46	LE
	**3.84**	**0.35**	**LE**	**3.05**	**0.47**	**LE**	**3.66**	**0.12**	**LE**	**3.21**	**0.32**	**LE**
S_2	5.12	0.02	CT	3.49	0.00	CT	5.18	0.00	CT	3.57	0.00	CT
	**4.16**	**0.00**	**CT**	**3.31**	**0.00**	**CT**	**4.14**	**0.00**	**CT**	**3.30**	**0.00**	**CT**
S_3	6.17	0.55	LE	4.36	0.23	LE	6.28	0.06	LE	4.39	0.16	LE
	**4.86**	**0.65**	**LE**	**4.20**	**0.23**	**LE**	**4.43**	**0.73**	**LE**	**3.91**	**0.30**	**LE**
**Geom 3**												
S_1	3.74	0.00	CT	2.97	0.00	CT	3.66	0.00	CT	2.95	0.00	CT
	**3.72**	**0.01**	**CT**	**2.82**	**0.00**	**CT**	**3.32**	**0.08**	**LE**	**2.81**	**0.00**	**CT**
S_2	3.88	0.31	LE	3.17	0.49	LE	3.88	0.31	LE	3.19	0.49	LE
	**3.79**	**0.28**	**LE**	**3.03**	**0.49**	**LE**	**4.46**	**0.43**	**LE**	**3.12**	**0.42**	**LE**
S_3	4.79	0.67	LE	4.22	0.21	LE	4.79	0.68	LE	4.23	0.22	LE
	**4.71**	**0.72**	**LE**	**4.05**	**0.22**	**LE**	**4.49**	**0.32**	**LE**	**3.63**	**0.24**	**LE**
**Geom 4**												
S_1	3.75	0.15	LE	3.35	0.19	LE	3.75	0.15	LE	3.36	0.19	LE
	**3.63**	**0.14**	**LE**	**3.23**	**0.18**	**LE**	**3.61**	**0.07**	**LE**	**3.29**	**0.00**	**CT**
S_2	3.94	0.00	CT	3.49	0.00	CT	3.89	0.00	CT	3.55	0.00	CT
	**3.89**	**0.00**	**CT**	**3.35**	**0.00**	**CT**	**4.07**	**0.00**	**CT**	**3.46**	**0.17**	**LE**
S_3	4.80	0.53	LE	4.56	0.32	LE	4.80	0.53	LE	4.57	0.32	LE
	**4.67**	**0.56**	**LE**	**4.39**	**0.33**	**LE**	**4.61**	**0.68**	**LE**	**4.07**	**0.37**	**LE**
**Geom 5**												
S_1	3.73	0.18	LE	3.21	0.44	LE	3.73	0.18	LE	3.21	0.43	LE
	**3.21**	**0.11**	**LE**	**3.00**	**0.15**	**LE**	**3.12**	**0.20**	**LE**	**2.61**	**0.25**	**LE**
S_2	4.06	0.00	CT	3.47	0.00	CT	3.97	0.00	CT	3.55	0.00	CT
	**3.83**	**0.00**	**CT**	**3.36**	**0.00**	**CT**	**3.88**	**0.01**	**CT**	**3.39**	**0.00**	**CT**
S_3	4.74	0.78	LE	4.05	0.24	LE	4.74	0.78	LE	4.07	0.24	LE
	**4.22**	**0.49**	**LE**	**4.09**	**0.28**	**LE**	**4.11**	**0.44**	**LE**	**3.87**	**0.26**	**LE**

5) Page 9. It is not completely clear how the authors distinguish the electronic character of the S1 state minima. Are they using the charge distribution and dipole moments?

Response:

Yes, we used dipole moments and the total S_1-state Mulliken charges on the FAD moiety and

Texas Tech University
Department of Chemistry and Biochemistry
1204 Boston Avenue
Lubbock, Texas 79409-1061

Ruibin Liang
Assistant Professor
806-834-7183
rliang@ttu.edu

W400 residue involved in the ET reaction to distinguish the electronic character of the S_1 state minima.

Revisions:

The following text is added to the main text, page 14:

The electronic characters of the S_1 state minima were assigned based on the dipole moments and the total S_1-state Mulliken charges on the FAD moiety and W400.

6) Page 12. The acronym TBF has to be defined.

Response:

We thank the reviewer for the observation. The definition of the trajectory basis functions (TBFs) is included at their first occurrence in the manuscript to improve clarity.

Revisions:

- The following text is added to page 12 in the main text and the Fig. 3 caption:

"To analyze the change in the character of excited-state electronic wavefunctions associated with the $S_2 \rightarrow S_1$ decay, we tracked the distribution of excited-state dipole moments for the ensemble of trajectory basis functions (TBFs) throughout the SSAIMS simulation."

Figure 3. (A) The time evolution of the populations of the S_1 and S_2 excited states in AtCRY1 following photoexcitation to the S_2 state with bright LE character, extracted from the SS-AIMS non-adiabatic dynamics simulations coupled with the SA-4-CASSCF(6,6)/6-31G*/MM method. The statistical uncertainties of each curve were computed using the bootstrapping analysis with 1000 samples. (B) The time evolution of the distribution of the excited-state dipole moment (in Debye) in the O-SS-AIMS non-adiabatic dynamics simulations. The dipole moments were analyzed from all trajectory basis functions (TBFs) during the SS-AIMS dynamics. The time-dependent distribution was generated by convolving the dipole moments using fixed-width 2D Gaussians with time-dependent amplitudes of the TBFs (SI method).

Texas Tech University
Department of Chemistry and Biochemistry
1204 Boston Avenue
Lubbock, Texas 79409-1061

Ruibin Liang
Assistant Professor
806-834-7183
rliang@ttu.edu

7) Figure 4. Is the total estimated barrier for the thermally driven transformation of LE to CT in S1 (i.e. adiabatically) ca. 6 kcal mol? How does this barrier, that would certainly slow down the thermal adiabatic process, compete in speed with the second ET occurring from a different tryptophan to W400•+ ?

Response and Revision:

The following text is added to the main text, page 16:

The total estimated barrier for the entire adiabatic transformation from the low-energy LE minima to the CT minima is ~6 kcal/mol. This barrier, after single-point energy corrections at XMS-CASPT2/SA-4-CASSCF(8,8) level of theory, is reduced to ~3.5 kcal/mol **(Figure S8 & S9)**. Considering nuclear quantum effects such as ZPE and tunneling, as well as further possible corrections to the PES by more accurately incorporating dynamic electron correlation, this adiabatic LE to CT barrier is consistent with an experimental rate of 0.4 ps[11], which is faster than the second ET steps from the W377 to the W300, which occurs in the range of 4-15 ps[11].

The following text is added to the main text, page 19:

These findings indicate that the initial photoinduced ET can occur adiabatically on the S_1 state following photoexcitation to the S_1 state, resulting in the formation of the radical pair. However, the S_1-state energy barrier may slow this process. We note that including dynamic correlation in the electronic structure method can reduce the adiabatic barrier, and increase the calculated rate of the adiabatic ET events that is consistent with the 0.4 ps time constant measured by the experiment[11]. However, the non-adiabatic ET provides an alternative ultrafast route to complement the adiabatic one, facilitating radical pair formation. This ultrafast non-adiabatic ET dynamics occurring within 10 fs is beyond the current time resolution of transient absorption spectroscopy. Additionally, thermal fluctuations on the ground state are essential for non-adiabatic ET events by means of changing the state order in the FC region.

Texas Tech University
Department of Chemistry and Biochemistry
1204 Boston Avenue
Lubbock, Texas 79409-1061

Ruibin Liang
Assistant Professor
806-834-7183
rliang@ttu.edu

8) Page 15. It is important to characterize the S2/S1 conical intersection to plot the dipole moment along a circle centered on the MECI intersection. The author should see a continuous change from lower to higher dipole moments.

Response:

We thank the reviewer for suggesting an improved characterization of the MECI geometry based on the electric dipole moment. The corresponding analysis is included in the Supplementary Information.

Revisions:

The following analysis of the electric dipole moment (in Debye) is added to the SI, pages S30-S31:

To characterize the S_2/S_1 MECI, the dipole moment (in Debye) was evaluated over a circular path on the branching plane of the MECI. The path is centered at the MECI between the S_1 and S_2 states. The plane was spanned by the difference in the gradients of the two states **g** and the nonadiabatic coupling vector **h between them**, given by the following equations:

$$\mathbf{g} = \frac{1}{2}\left(\frac{\partial E_2}{\partial \mathbf{R}} - \frac{\partial E_1}{\partial \mathbf{R}}\right) \quad \text{Eq. S1}$$

$$\mathbf{h} = \left\langle \phi_2 \middle| \frac{\partial H}{\partial \mathbf{R}} \middle| \phi_1 \right\rangle \quad \text{Eq. S2}$$

The vectors **g** and **h** were then orthonormalized to expand the branching plane. Geometries along this circular path on this plane were then generated according to:

$$x_p = x_o + \mathrm{R} \cdot (cos\,\theta \cdot \mathrm{g} + sin\,\theta \cdot \mathrm{h}), \quad \text{Eq. S3}$$

where x_o represents the full Cartesian coordinates of the MECI, R is the displacement radius (1 Å), and θ is the polar angle in the branching plane relative to the **g** vector and changes from 0 to

Texas Tech University
Department of Chemistry and Biochemistry
1204 Boston Avenue
Lubbock, Texas 79409-1061

Ruibin Liang
Assistant Professor
806-834-7183
rliang@ttu.edu

360 degrees. All single-point calculations along the circular paths were performed at the SA-4-CASSCF(6,6)/6-31G*/MM level of theory. The initial guess of the CASSCF wavefunction for each point was obtained from an adjacent geometry with a lower θ value.

Figure S10 summarizes the results of such MECI characterization. It is evident that the S_1-state dipole moment continuously changes from lower to higher values and then back to lower values. This process corresponds to the S_1 wavefunction's character changing from LE to CT, and finally to LE along the circular path on the branching plane. This result confirms that the S_2/S_1 MECI arises from the intersection between two diabatic electronic states, one with LE character and the other with CT character.

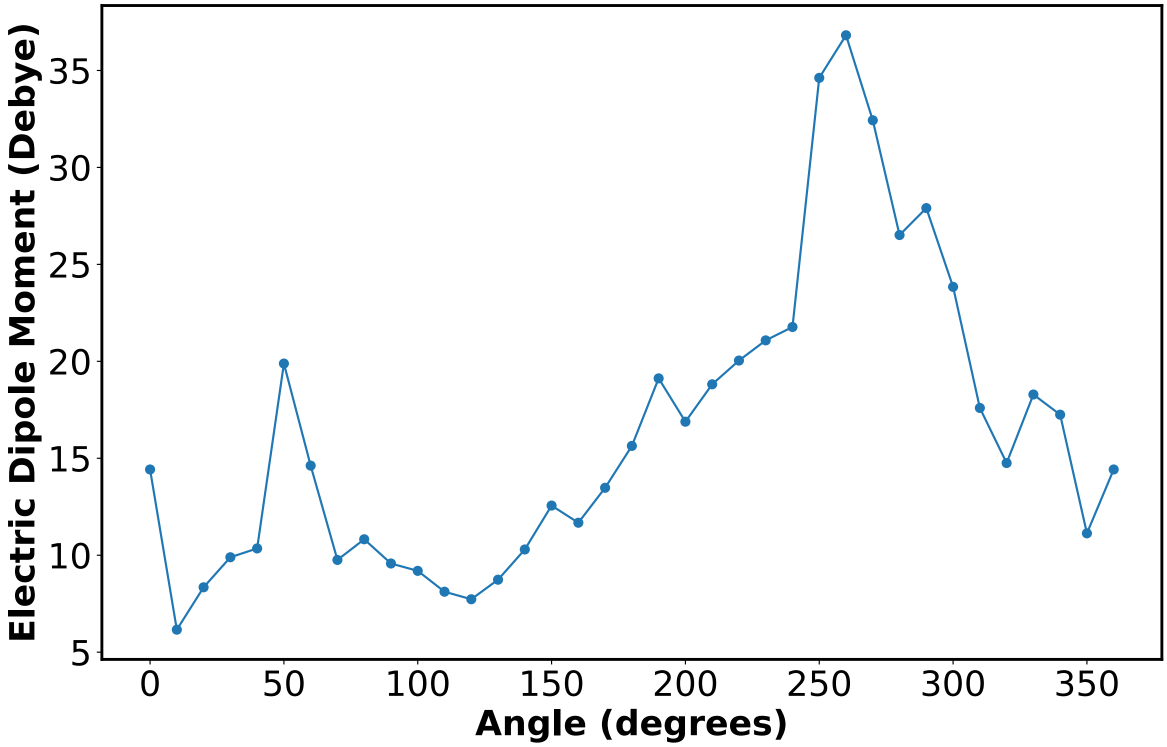

Figure S10. S_1-state dipole moment along the circular path on the branching plane of S_2/S_1 MECI. The branching plane is expanded by the orthonormalized **g** and **h** vectors. The S_2/S_1 MECI is

Texas Tech University
Department of Chemistry and Biochemistry
1204 Boston Avenue
Lubbock, Texas 79409-1061

Ruibin Liang
Assistant Professor
806-834-7183
rliang@ttu.edu

located at the origin of this plane and the center of the circular path around it. The MECI was optimized at the SA-4-CASSCF(6,6)/6-31G*/MM level of theory, and all single-point calculations were performed at the same level of theory. The circular path has a radial distance of 1Å from the MECI, and polar angles were sampled in intervals of 10 degrees along this path.

9) Page 15. Define NEB.

Response:

We thank the reviewer for the observation. The definition of the Nudged Elastic Band (NEB) method will be included at its first occurrence in the manuscript to improve clarity.

Revisions:

The following sentence has been updated in the main text, page 16:

"To estimate the magnitude of the barriers, the nudged elastic band (NEB) method was employed to optimize the minimum energy paths (MEPs) connecting the LE and CT minima on the S_1 state at the SA-4-CASSCF(6,6)/6-31G*/MM level of theory in the *At*CRY1 (see Methods)."

10) Figure 4. What are the differences between CASSCF and XMS-CASPT2 in terms of energy profiles? At which level the adiabatic barrier is computed? How does the dynamic electron correlation affect the barrier? Why not to do single point corrections? This has to be discussed in the main text.

Response:

Texas Tech University
Department of Chemistry and Biochemistry
1204 Boston Avenue
Lubbock, Texas 79409-1061

Ruibin Liang
Assistant Professor
806-834-7183
rliang@ttu.edu

The adiabatic barrier is calculated at the SA-CASSCF level of theory. We have performed additional XMS-CASPT2(8,8) single-point energy corrections on top of the S1 MEPs optimized by the SA-CASSCF level of theory. The results are summarized in **Figures S8 & S9**. Adding the dynamic electron correlation reduces the barrier to about 3.5 kcal/mol.

Revisions:

Please see our revision in response to comment 7.

11) Page 18. Is it then concluded that the system that upon excitation reach S1 never reach the CT minimum?

Response:

We apologize that we don't fully understand this question. On page 18, we didn't conclude that upon excitation, the system never reach the CT minimum. Our conclusion was that, the adiabatic ET on the S1 state is slowed down by the low energy LE minimum, and it will eventually reach the CT minimum.

Revisions:

We rephrased our statement in main text, page 19:

Taken together, these observations suggest that many S_1 state trajectories are temporarily stabilized in the low-energy LE minima, slowing down the access to the CT minima, in agreement with our above-mentioned MEP analysis.

12) Figure 5. The figure shows a surface touching (i.e. a degeneracy between two smooth potential energy surfaces. In reality conical intersections are real crossing and the point of crossing is a cusp on both S1 and S2. Please correct.

Response:

Texas Tech University
Department of Chemistry and Biochemistry
1204 Boston Avenue
Lubbock, Texas 79409-1061

Ruibin Liang
Assistant Professor
806-834-7183
rliang@ttu.edu

We are grateful to the reviewer for highlighting the correct topological features of the conical intersection. Accordingly, the surface topology has been adjusted to clearly represent the cusp at the excited-state crossing.

Revisions:

Figure 5E has been revised according to the reviewer's suggestion. The cusp character of the S_1 and S_2 surfaces is emphasized now.

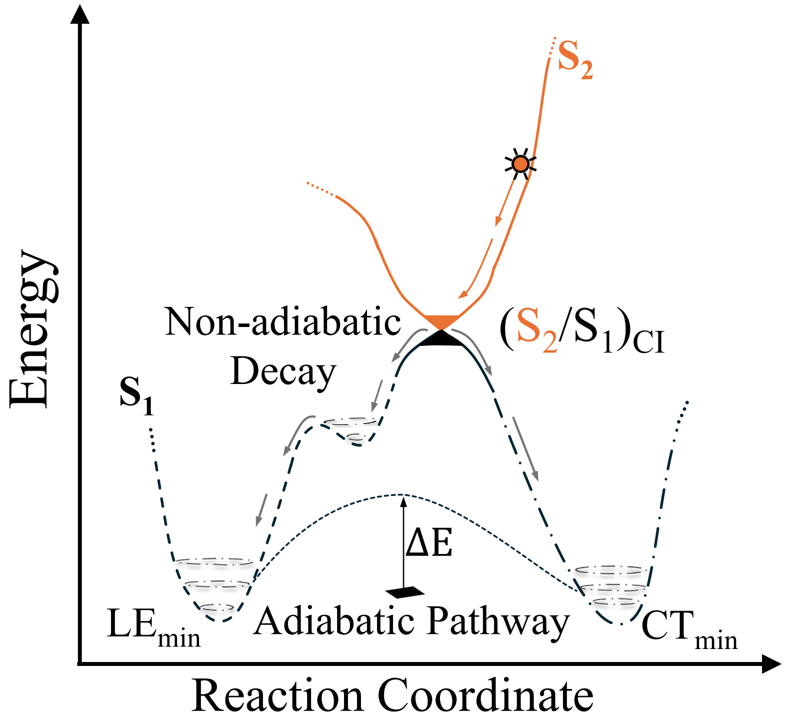

13) I am not convinced that the real motivation for a protein environment stopping the adiabatic LE->CT process is connected to the need for making the next ET step more efficient or even possible. While this remains a possibility based on the qualitative model presented in the manuscript, the author should also consider other hypotheses (see also above).

Texas Tech University
Department of Chemistry and Biochemistry
1204 Boston Avenue
Lubbock, Texas 79409-1061

Ruibin Liang
Assistant Professor
806-834-7183
rliang@ttu.edu

Responses and Revisions:

We thank the reviewer for this comment. The following is revised in the main text, pages 21-22:

Overall, our qualitative analysis based on single-point energy calculations and local geometry optimizations does not directly reveal how the protein environment facilitates the transition from the LE minimum to the CT minimum on the S_1 state. However, we reason that without the geometric constraints imposed by the protein, random fluctuations in the orientation and distance between the donor and acceptor can impede the ET. In this way, the protein environment plays a crucial role in the initial photoinduced ET step by limiting the conformational flexibility of the FAD-W400 pair. Additionally, the non-adiabatic ET pathway, which is sensitive to conformational sampling near the Franck-Condon region (see above), also benefits from these constraints. By restricting thermal fluctuations within a narrowly distributed structural ensemble, the protein effectively positions the structures of the FAD and W400 near the S_2/S_1 conical intersection seam, and also allows direct photoexcitation to the S_2 state with LE character to initiate the non-adiabatic ET dynamics.

The above analysis also raises an interesting question: how can the *At*CRY1 facilitate the propagation of the [$FAD^{\bullet-}$-$W400^{\bullet+}$] radical pair through the tryptophan triad? We reason that the constraints imposed by the protein environment may ensure the correct relative orientation between W400 and its neighboring W377 that maximizes their overlap in molecular orbitals, resulting in optimal diabatic coupling to facilitate the next step of the ET from W400 to W377. In **Fig. 5C&D,** we illustrate how the protein restricts and stabilizes orientations of the FWD complex with respect to the W377, compared to the optimized structures of FDW in the vacuum. Thus, we hypothesize that the protein environment can speed up the subsequent ET steps between the tryptophan residues. This hypothesis needs to be tested in future work using Marcus's theory in the vacuum and protein environments for subsequent ET steps.

pages 26:

Last but not least, the electrostatics environment created by the protein stabilize the LE minimum more than the CT minimum on the S_1 state, seemingly disfavoring the initial ET event. However, the arrangements of the side chains of the tryptophan residues and the FAD in the protein could ensure good overlap in the molecular orbitals between them, thus enabling thus enabling quick ET events to occur both non-adiabatically and adiabatically. Without the protein's steric constraints, large reorientation of the tryptophan residues and the FAD can make ET difficult. *Thus, the protein environment facilitates the kinetics of different ET steps by steric constraints to maximize the overall quantum efficiency.* This new interpretation of the role of protein on the ET in cryptochromes deepens our understanding of photoreactions in biomolecules.

Texas Tech University
Department of Chemistry and Biochemistry
1204 Boston Avenue
Lubbock, Texas 79409-1061

Ruibin Liang
Assistant Professor
806-834-7183
rliang@ttu.edu

The Abstract is revised to reflect this change:

Cryptochromes are blue light photoreceptors in organisms from plants to animals that are essential for circadian rhythms, phototropism, and magnetoreception. In light-sensing cryptochromes, the photoexcitation of the flavin adenine dinucleotide (FAD) cofactor triggers a cascade of electron transfer events via a tryptophan chain, eventually generating a radical pair crucial for signaling. Despite extensive studies, the initial photoinduced electron transfer (ET) from a neighboring tryptophan residue to FAD remains unclear due to the complexity of simulating all-atom dynamics in excited states, particularly regarding the roles of non-adiabatic pathways and protein environment on the reaction kinetics and quantum efficiency of the ET. To address this gap, we performed extensive non-adiabatic and adiabatic dynamics simulations with on-the-fly multireference *ab initio* electronic structure calculations of *Arabidopsis thaliana* cryptochrome 1 (*At*CRY1). Our results reveal a novel mechanism in which rapid non-radiative decay from higher-lying singlet states leads to charge separation, complementing the slower adiabatic ET on the S_1 state hindered by a newly identified low-energy S_1 local excitation minimum. Furthermore, the protein environment stabilizes tryptophan orientations, facilitating subsequent electron transfer steps. These insights significantly enhance our understanding of photoinduced ET in cryptochromes and the structure-function relationships in photoreceptors.

I hope this letter addresses the questions and comments from the reviewers, and that the manuscript is now suitable for publication. Please do not hesitate to contact me if there are any questions.

Sincerely,

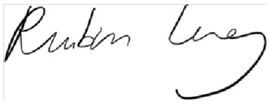

Ruibin Liang

Texas Tech University
Department of Chemistry and Biochemistry
1204 Boston Avenue
Lubbock, Texas 79409-1061

Ruibin Liang
Assistant Professor
806-834-7183
rliang@ttu.edu

References

(1) Solov'yov, I. A.; Domratcheva, T.; Moughal Shahi, A. R.; Schulten, K. Decrypting Cryptochrome: Revealing the Molecular Identity of the Photoactivation Reaction. *J. Am. Chem. Soc.* **2012**, *134* (43), 18046-18052. DOI: 10.1021/ja3074819.
(2) Ibele, L. M.; Lassmann, Y.; Martínez, T. J.; Curchod, B. F. E. Comparing (stochastic-selection) ab initio multiple spawning with trajectory surface hopping for the photodynamics of cyclopropanone, fulvene, and dithiane. *J. Chem. Phys.* **2021**, *154* (10), 104110. DOI: 10.1063/5.0045572.
(3) Curchod, B. F. E.; Glover, W. J.; Martínez, T. J. SSAIMS—Stochastic-Selection Ab Initio Multiple Spawning for Efficient Nonadiabatic Molecular Dynamics. *J. Phys. Chem. A* **2020**, *124* (30), 6133-6143. DOI: 10.1021/acs.jpca.0c04113.
(4) Barbatti, M.; Sen, K. Effects of different initial condition samplings on photodynamics and spectrum of pyrrole. *Int. J. Quantum Chem.* **2016**, *116* (10), 762-771. DOI: https://doi.org/10.1002/qua.25049.
(5) Frederiksen, A.; Gerhards, L.; Reinholdt, P.; Kongsted, J.; Solov'yov, I. A. Importance of Polarizable Embedding for Absorption Spectrum Calculations of Arabidopsis thaliana Cryptochrome 1. *J. Phys. Chem. B* **2024**, *128* (26), 6283-6290. DOI: 10.1021/acs.jpcb.4c02168.
(6) Yanagawa, M.; Kojima, K.; Yamashita, T.; Imamoto, Y.; Matsuyama, T.; Nakanishi, K.; Yamano, Y.; Wada, A.; Sako, Y.; Shichida, Y. Origin of the low thermal isomerization rate of rhodopsin chromophore. *Sci. Rep.* **2015**, *5* (1), 11081. DOI: 10.1038/srep11081.
(7) Birge, R. R.; Barlow, R. B. On the molecular origins of thermal noise in vertebrate and invertebrate photoreceptors. *Biophys. Chem.* **1995**, *55* (1), 115-126. DOI: https://doi.org/10.1016/0301-4622(94)00145-A.
(8) Yau, K.-W.; Matthews, G.; Baylor, D. Thermal activation of the visual transduction mechanism in retinal rods. *Nature* **1979**, *279* (5716).

Texas Tech University
Department of Chemistry and Biochemistry
1204 Boston Avenue
Lubbock, Texas 79409-1061

Ruibin Liang
Assistant Professor
806-834-7183
rliang@ttu.edu

(9) Barlow, R. B.; Birge, R. R.; Kaplan, E.; Tallent, J. R. On the molecular origin of photoreceptor noise. *Nature* **1993**, *366* (6450), 64-66. DOI: 10.1038/366064a0.
(10) Timmer, D.; Frederiksen, A.; Lünemann, D. C.; Thomas, A. R.; Xu, J.; Bartölke, R.; Schmidt, J.; Kubař, T.; De Sio, A.; Solov'yov, I. A.; Mouritsen, H.; Lienau, C. Tracking the Electron Transfer Cascade in European Robin Cryptochrome 4 Mutants. *J. Am. Chem. Soc.* **2023**, *145* (21), 11566-11578. DOI: 10.1021/jacs.3c00442.
(11) Immeln, D.; Weigel, A.; Kottke, T.; Pérez Lustres, J. L. Primary Events in the Blue Light Sensor Plant Cryptochrome: Intraprotein Electron and Proton Transfer Revealed by Femtosecond Spectroscopy. *J. Am. Chem. Soc.* **2012**, *134* (30), 12536-12546. DOI: 10.1021/ja302121z.
(12) Liu, Z.; Tan, C.; Guo, X.; Li, J.; Wang, L.; Sancar, A.; Zhong, D. Determining complete electron flow in the cofactor photoreduction of oxidized photolyase. *Proc. Natl. Acad. Sci. U. S. A.* **2013**, *110* (32), 12966-12971. DOI: 10.1073/pnas.1311073110.
(13) Martin, R.; Lacombat, F.; Espagne, A.; Dozova, N.; Plaza, P.; Yamamoto, J.; Müller, P.; Brettel, K.; de la Lande, A. Ultrafast flavin photoreduction in an oxidized animal (6-4) photolyase through an unconventional tryptophan tetrad. *Phys. Chem. Chem. Phys.* **2017**, *19* (36), 24493-24504, 10.1039/C7CP04555G. DOI: 10.1039/C7CP04555G.
(14) Kar, R. K.; Miller, A.-F.; Mroginski, M.-A. Understanding flavin electronic structure and spectra. *WIREs Computational Molecular Science* **2022**, *12* (2), e1541. DOI: https://doi.org/10.1002/wcms.1541.

oc-2025-00376z.R2

Name: Peer Review Information for "Decrypting the Non-Adiabatic Photoinduced Electron Transfer Mechanism in Light-Sensing Cryptochrome"

Second Round of Reviewer Comments

Reviewer: 1

Comments to the Author

I thank the authors for their efforts in addressing my concerns and questions. I particularly appreciate the additional calculations performed, which have reinforced this work and its conclusions. I would recommend publication of this work.

Reviewer: 2

Comments to the Author

On the basis of my reading of the author's replies (to both reviewers), I am now able to recommend acceptance after the authors have dealt with the following minor points (re-reviewing is not necessary).

1) The answer to my General Comment I regarding the possible role of the non-adiabatic ultrafast electron transfer mechanism is satisfactory. However, even if the non-adiabatic process decreases (does not eliminate) the thermal noise, the underlying mechanism remains the same seem in rhodopsin. In other words, the difference is quantitative (i.e. the much slower adiabatic process in rhodopsin) not qualitative. The included references on the thermal noise in rhodopsins are seminal but not up-to-date. I ask the author to consider citing more closely related computational studies such as Gozem, S. et al. The Molecular Mechanism of Dark Noise in Rod Photoreceptors. Science 337, 1225–1228

(2012), Luk, H. L. et al. Modulation of Thermal Noise and Spectral Sensitivity in Lake Baikal Cottoid Fish Rhodopsins. Sci. Rep. 6, 38425 (2016).

2) Reply to my General Comment II. From the author's reply, it seems that the ultrafast non-adiabatic process cannot be presently observed. Only the picosecond adiabatic S1 process can. Does this mean that the non-adiabatic process is a mere theoretical prediction to be capture with future experiments performed at an higher time resolution? It is not clear if the study suggests that the non adiabatic process will have an impact on the general quantum efficiency of the reaction and not only help to decrease the thermal noise?

3) Author's reply to point 4. Right, but in order to reproduce the author’s result one needs to know exactly how to choose the 6 or 8 orbitals among the numerous pi-orbitals of the chromophore. This may not be trivial. Maybe the authors should report in the Supporting information the plots of the selected orbitals for the 6,6 and 8,8 active space and/or report a selection criterium.

4) Figure S10. The figure is "qualitatively acceptable". However, I wonder why the dipole calculation show such lack of smoothness. This seems related to some instability (orbital convergence? orbital guess) in the region. A plot of the S1 and S2 energy profiles will definitely help to document or explain the issue. Furthermore it should be interesting to document which of the two branching plane vectors drives the dipole moment change.

Author's Response to Peer Review Comments:

Texas Tech University
Department of Chemistry and Biochemistry
1204 Boston Avenue
Lubbock, Texas 79409-1061

Ruibin Liang
Assistant Professor
806-834-7183
rliang@ttu.edu

May 12th, 2025

Senior Editor
ACS Central Science

Dear Editor,

We thank the reviewers for the comments. We have addressed the comments as detailed below. The reviewer's comments are shown in green and modified texts in the revised manuscript are shown in blue. The page numbers mentioned in our response refer to the track-change version of the manuscript uploaded as a supplementary document for reviewers. The table and figure numbers refer to the revised version of the main text and SI. Reference numbers refer to this response letter.

Reviewer 1

I thank the authors for their efforts in addressing my concerns and questions. I particularly appreciate the additional calculations performed, which have reinforced this work and its conclusions. I would recommend publication of this work.

We thank the reviewer for carefully evaluating our revised manuscript and supplemental materials, and for providing constructive suggestions regarding the benchmarking strategy, which have helped improve the reproducibility and transparency of our work.

Reviewer 2

Texas Tech University
Department of Chemistry and Biochemistry
1204 Boston Avenue
Lubbock, Texas 79409-1061

Ruibin Liang
Assistant Professor
806-834-7183
rliang@ttu.edu

On the basis of my reading of the author's replies (to both reviewers), I am now able to recommend acceptance after the authors have dealt with the following minor points (re-reviewing is not necessary).

We thank the reviewer for carefully evaluating our revised manuscript and providing constructive suggestions.

Specific comments:

1) The answer to my General Comment I regarding the possible role of the non-adiabatic ultrafast electron transfer mechanism is satisfactory. However, even if the non-adiabatic process decreases (does not eliminate) the thermal noise, the underlying mechanism remains the same seem in rhodopsin. In other words, the difference is quantitative (i.e. the much slower adiabatic process in rhodopsin) not qualitative. The included references on the thermal noise in rhodopsins are seminal but not up-to-date. I ask the author to consider citing more closely related computational studies such as Gozem, S. et al. The Molecular Mechanism of Dark Noise in Rod Photoreceptors. Science 337, 1225–1228 (2012), Luk, H. L. et al. Modulation of Thermal Noise and Spectral Sensitivity in Lake Baikal Cottoid Fish Rhodopsins. Sci. Rep. 6, 38425 (2016).

Response:

We thank the reviewer for the comment. The two studies mentioned by the reviewer are now cited.

Revisions:

The following is added to page 26.

Despite this quantitative difference, the mechanism underlying thermal noise suppression in *At*CRY1 remains qualitatively the same as the one observed in rhodopsins[1, 2].

Specific comments:

Texas Tech University
Department of Chemistry and Biochemistry
1204 Boston Avenue
Lubbock, Texas 79409-1061

Ruibin Liang
Assistant Professor
806-834-7183
rliang@ttu.edu

2) Reply to my General Comment II. From the author's reply, it seems that the ultrafast non-adiabatic process cannot be presently observed. Only the picosecond adiabatic S1 process can. Does this mean that the non-adiabatic process is a mere theoretical prediction to be capture with future experiments performed at an higher time resolution? It is not clear if the study suggests that the non adiabatic process will have an impact on the general quantum efficiency of the reaction and not only help to decrease the thermal noise?

Response:

We thank the reviewer for the comment. Our current work highlights the role of the novel non-adiabatic ET mechanism in facilitating the ET relative to the adiabatic mechanism. However, a statistically meaningful quantum yield comparison can only be obtained by observing many more ET events (perhaps on the order of hundreds) occurring through the adiabatic and non-adiabatic pathways than the scope of our simulation data. Thus, we cannot conclude definitely that the non-adiabatic ET process will increase or decrease the overall quantum yield compared to the adiabatic ET process.

3) Author's reply to point 4. Right, but in order to reproduce the author's result one needs to know exactly how to choose the 6 or 8 orbitals among the numerous pi-orbitals of the chromophore. This may not be trivial. Maybe the authors should report in the Supporting information the plots of the selected orbitals for the 6,6 and 8,8 active space and/or report a selection criterium.

Response and Revisions:

We add the following discussion and data to SI page S23-24.

To further validate our choices of the (6e,6o) and (8e,8o) active space, we evaluated the occupation numbers of both occupied and virtual natural orbitals after the SA-CASSCF calculations for five distinct geometries in the FC region, as summarized in **Table S4**. In both SA-4-CASSCF(6e,6o) and SA-4-CASSCF(8e,8o) calculations, the occupancy numbers of natural orbitals of the S_1 and

Texas Tech University
Department of Chemistry and Biochemistry
1204 Boston Avenue
Lubbock, Texas 79409-1061

Ruibin Liang
Assistant Professor
806-834-7183
rliang@ttu.edu

S_2 states quickly decay to near zero for orbitals above LOMO ($\boldsymbol{\pi_1^*}$), and quickly increase to near two below HOMO ($\boldsymbol{\pi_4}$). This analysis suggests that the dominant electronic excitations in the S_1 and S_2 states are localized near these two orbitals, and both active spaces are large enough to describe the multireference electronic wavefunctions associated with them. Expanding the (6e, 6o) into the (8e, 8o) active spaces adds one π and one π^* orbital localized on the W400 residue (Figure S1).

Table S4. The occupation numbers of natural orbitals of S_1 and S_2 states obtained from SA-CASSCF/MM calculations with (6e, 6o) and (8e, 8o) active spaces. The analysis was performed at ground-state geometries equilibrated in the FC region within the protein environment.

State	$\boldsymbol{\pi_1}$	$\boldsymbol{\pi_2}$	$\boldsymbol{\pi_3}$	$\boldsymbol{\pi_4}$	$\boldsymbol{\pi_1^*}$	$\boldsymbol{\pi_2^*}$	$\boldsymbol{\pi_3^*}$	$\boldsymbol{\pi_4^*}$
**Geom 1**								
S_1 (8e,8o)	1.97	1.95	1.95	1.01	1.00	0.05	0.04	0.03
S_2 (8e,8o)	2.00	1.94	1.92	1.01	0.98	0.06	0.05	0.04
S_1 (6e,6o)		1.95	1.95	1.01	0.99	0.05	0.04	
S_2 (6e,6o)		2.00	1.94	1.34	0.65	0.06	0.01	
**Geom2**								
S_1 (8e,8o)	1.98	1.94	1.90	1.26	0.73	0.08	0.06	0.06
S_2 (8e,8o)	1.95	1.93	1.92	1.12	0.88	0.08	0.07	0.06
S_1 (6e,6o)		2.00	1.88	1.30	0.71	0.08	0.04	
S_2 (6e,6o)		1.94	1.92	1.00	1.00	0.08	0.06	
**Geom 3**								
S_1 (8e,8o)	1.94	1.94	1.94	1.02	0.98	0.06	0.06	0.05
S_2 (8e,8o)	2.00	1.96	1.89	1.34	0.67	0.06	0.04	0.03
S_1 (6e,6o)		1.94	1.94	1.03	0.97	0.06	0.06	
S_2 (6e,6o)		2.00	1.89	1.34	0.67	0.06	0.03	
**Geom 4**								
S_1 (8e,8o)	2.00	1.95	1.90	1.24	0.75	0.08	0.05	0.04
S_2 (8e,8o)	1.94	1.94	1.93	1.00	1.00	0.07	0.06	0.06
S_1 (6e,6o)		2.00	1.90	1.24	0.75	0.08	0.04	
S_2 (6e,6o)		1.94	1.93	1.00	1.00	0.07	0.06	
**Geom 5**								

Texas Tech University
Department of Chemistry and Biochemistry
1204 Boston Avenue
Lubbock, Texas 79409-1061

Ruibin Liang
Assistant Professor
806-834-7183
rliang@ttu.edu

S_1 (8e,8o)	2.00	1.94	1.90	1.22	0.78	0.07	0.06	0.03
S_2 (8e,8o)	1.94	1.93	1.92	1.02	0.99	0.07	0.07	0.06
S_1 (6e,6o)		2.00	1.90	1.22	0.78	0.07	0.03	
S_2 (6e,6o)		1.94	1.93	1.01	0.99	0.07	0.06	

Figure S1 is modified in the SI on Page S13:

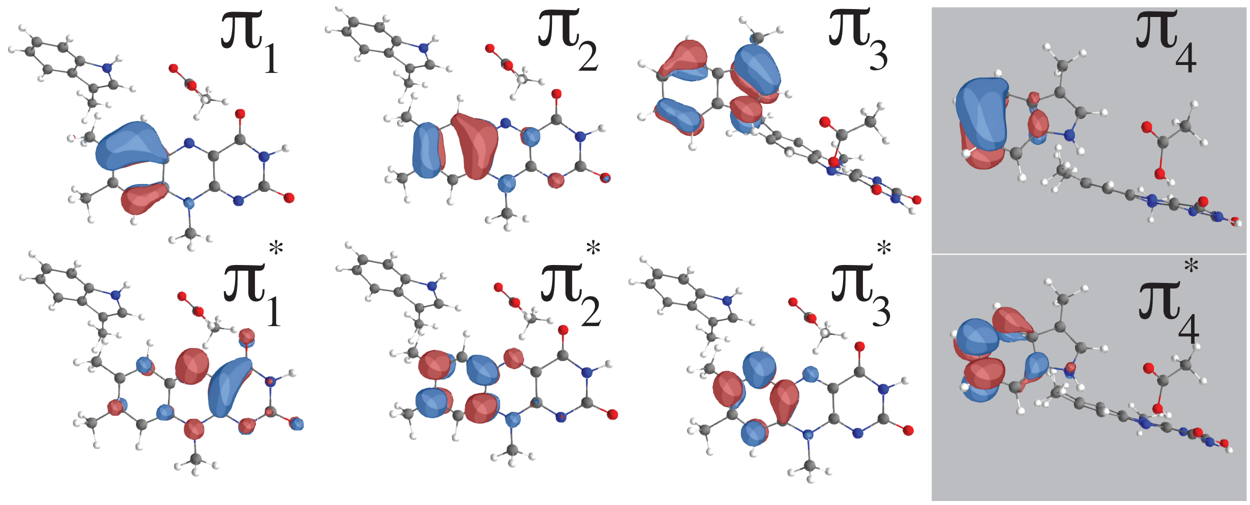

Figure S1. Qualitative representations of molecular orbitals in the active space of the SA-CASSCF(6e,6o) and SA-4-CASSCF(8e,8o) calculations. Orbitals with white backgrounds were included in both active spaces, and the additional two orbitals (π_4 and π_4^*) in gray backgrounds were included in the 8e,8o active space. The molecular structure was obtained from a ground-state QM/MM adiabatic simulation performed at the ωPBEh/6-31G*/MM level of theory. The ground-state (S_0) mainly comprises a closed-shell structure with about 90% weight. In this molecular configuration, the first singlet excited state (S_1) has a primary CT character involving a single-electron excitation from the π_3 orbital localized on the W400 residue to the π_1^* orbital on the FAD molecule ($\pi_3 \rightarrow \pi_1^*$).

4) Figure S10. The figure is "qualitatively acceptable". However, I wonder why the dipole calculation show such lack of smoothness. This seems related to some instability (orbital convergence? orbital guess) in the region. A plot of the S1 and S2 energy profiles will definitely

Texas Tech University
Department of Chemistry and Biochemistry
1204 Boston Avenue
Lubbock, Texas 79409-1061

Ruibin Liang
Assistant Professor
806-834-7183
rliang@ttu.edu

help to document or explain the issue. Furthermore it should be interesting to document which of the two branching plane vectors drives the dipole moment change.

Response

The original dipole moment figure (**Figure S10**) is replaced with a figure generated using shorter radius, increasing the smoothness of the curve. Regarding the sudden jump in the dipole moments at ~0 and ~180 degrees, and the following discussion is added to Page S32 of the SI.

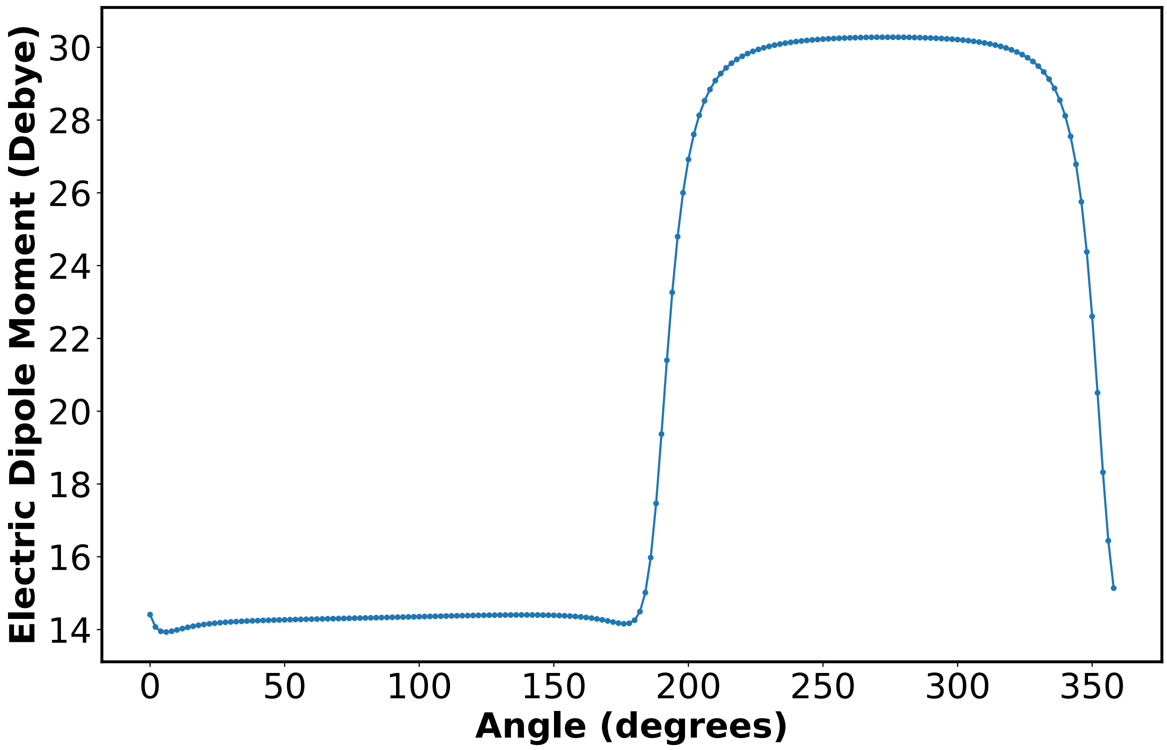

Figure S10. S1-state dipole moment along the circular path on the branching plane of S2/S1 MECI. The branching plane is expanded by the orthonormalized g and h vectors. The S2/S1 MECI is located at the origin of this plane and the center of the circular path around it. The MECI was optimized at the SA-4-CASSCF(6,6)/6-31G*/MM level of theory, and all single-point calculations were performed at the same level of theory. The circular path has a radial distance of 0.01Å from the MECI, and polar angles were sampled in intervals of 2 degrees along this path.

Texas Tech University
Department of Chemistry and Biochemistry
1204 Boston Avenue
Lubbock, Texas 79409-1061

Ruibin Liang
Assistant Professor
806-834-7183
rliang@ttu.edu

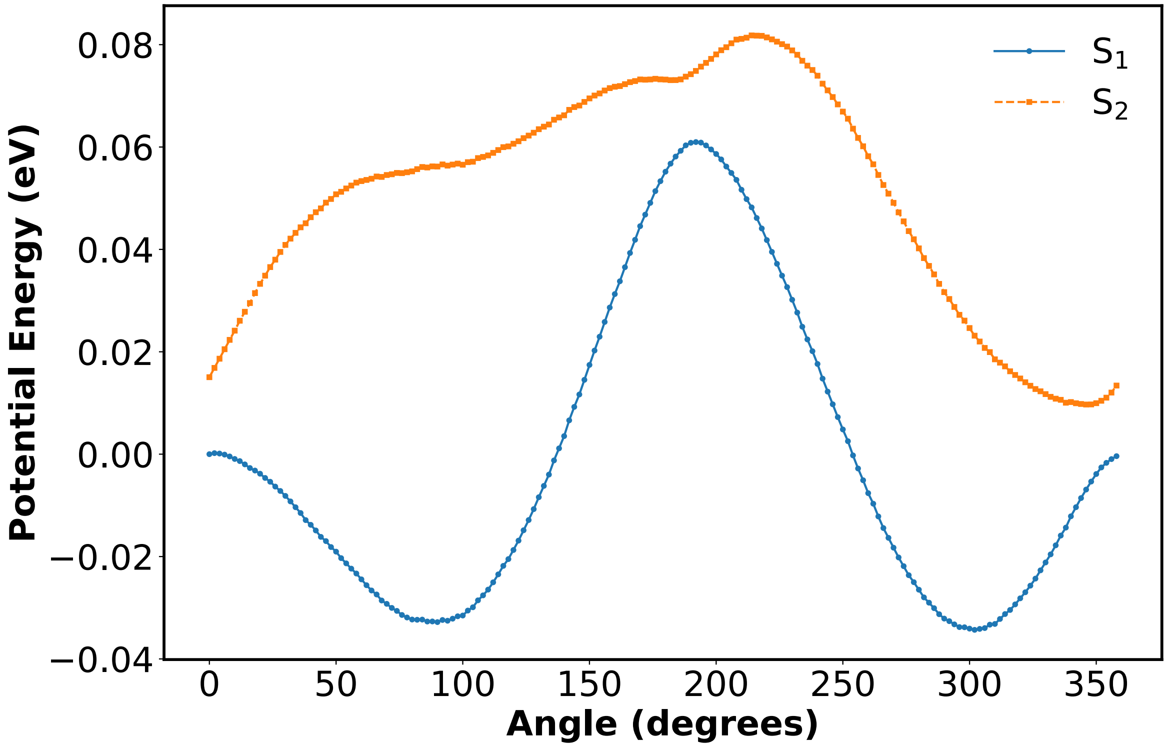

Figure S11. Potential energies of the S_1 and S2 states along the circular path on the branching plane of a S_2/S_1 MECI. The branching plane is expanded by the orthonormalized **g** and **h** vectors. The S_2/S_1 MECI is located at the origin of this plane and the center of the circular path around it. The MECI was optimized at the SA-4-CASSCF(6,6)/6-31G*/MM level of theory, and all single-point calculations were performed at the same level of theory. The circular path has a radial distance of 0.01Å from the MECI, and polar angles were sampled in intervals of 2 degrees along this path.

Revision:

The following is added to the SI page S32:

The sudden increase/decrease of dipole moment near ~0 and ~180 degrees (Figure S10) can be explained by the two diabatic surfaces (with LE and CT characters, respectively) crossing each other along the x axis. In the branching plane, the interface of the two PESs can be better described

Texas Tech University
Department of Chemistry and Biochemistry
1204 Boston Avenue
Lubbock, Texas 79409-1061

Ruibin Liang
Assistant Professor
806-834-7183
rliang@ttu.edu

as a pseudo one-dimensional curve following the **h** vector (x axis) rather than a single zero-dimensional point at the origin, as normally expected for geometries belonging to a typical CI seam space. In other words, the CI seam space here has pseudo 3N-7 degrees of freedom, although formally its total degrees of freedom is still 3N-8. This phenomenon arises from the fact that geometric displacements of the non-adiabatic coupling vector (**h**) only weakly lift the energy degeneracy at the CI geometry, whereas the perpendicular vector (dominated by the **g** vector) introduces a stronger lift of the degeneracy. The sudden change in the electronic wavefunction's character at 0 and 180 degrees is not due to the instability of the SA-CASSCF active space. The active space stability is confirmed by the continuity of the S_1 and S_2 adiabatic states' energies along the circular path on the branching plane around the MECI (Figure S11). Such a phenomenon is expected for typical ET reactions, considering the well-established success of the Marcus theory. The Marcus theory usually employs the 1D PESs of two intersecting diabatic states projected to one solvent reaction coordinate. The success of this 1D depiction of diabatic PESs implies that in the CI branching plane, the two 2D diabatic PESs mostly intersect to generate a pseudo 1D curve, along which the energy degeneracy is only weakly lifted and the diabatic coupling and adiabatic energy gap between the two states change slowly. Here, the **h** vector plays such a role. The geometry displacement along the perpendicular vector on the branching plane, dominated by the **g** vector, is the major driver for the electron transfer event, which is analogous to the reaction coordinate typically used in the Marcus theory.

I hope this letter addresses the questions and comments from the reviewers, and that the manuscript is now suitable for publication. Please do not hesitate to contact me if there are any questions.

Sincerely,

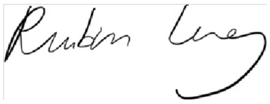

Ruibin Liang

Texas Tech University
Department of Chemistry and Biochemistry
1204 Boston Avenue
Lubbock, Texas 79409-1061

Ruibin Liang
Assistant Professor
806-834-7183
rliang@ttu.edu

References

(1) Gozem, S.; Schapiro, I.; Ferré, N.; Olivucci, M. The Molecular Mechanism of Thermal Noise in Rod Photoreceptors. *Science* **2012**, *337* (6099), 1225. DOI: 10.1126/science.1220461.
(2) Luk, H. L.; Bhattacharyya, N.; Montisci, F.; Morrow, J. M.; Melaccio, F.; Wada, A.; Sheves, M.; Fanelli, F.; Chang, B. S. W.; Olivucci, M. Modulation of thermal noise and spectral sensitivity in Lake Baikal cottoid fish rhodopsins. *Sci. Rep.* **2016**, *6* (1), 38425. DOI: 10.1038/srep38425.
